# Data related to the growth of σ-phase precipitates in CrMnFeCoNi high-entropy alloys: Temporal evolutions of precipitate dimensions and concentration profiles at interfaces

**DOI:** 10.1016/j.dib.2020.106449

**Published:** 2020-10-21

**Authors:** Guillaume Laplanche

**Affiliations:** Institute for Materials, Ruhr-University Bochum, Universitätsstr. 150, 44801 Bochum, Germany

**Keywords:** High-entropy alloys, CoCrFeMnNi, Allotriomorph, diffusion coefficients, TCP phase

## Abstract

A data compilation related to the growth kinetics of a topologically closed-packed (TCP) phase is reported. A high-entropy alloy (HEA) with a composition of Cr_26_Mn_20_Fe_20_Co_20_Ni_14_ in at.%, a mean grain size of 50 µm and initially single-phase face-centered cubic (FCC) was annealed at temperatures ranging from 600 °C to 1000 °C for times between 0.05 h and 1000 h. These heat treatments resulted in the formation of tetragonal σ precipitates that formed heterogeneously at different elements of the microstructure. The raw data of the present article include backscattered electron (BSE) micrographs where σ precipitates can be observed within grains, at grain boundaries, and triple points of the FCC matrix. From these images, the dimensions of the five largest precipitates observed within grains and those of the five largest allotriomorphs are provided for different times and temperatures in tables. As the σ precipitates are more enriched in Cr and depleted in Ni than the surrounding matrix, Cr-depleted (Ni-enriched) zones form in the FCC matrix next to the precipitates and widen at rates determined by diffusion. To document the evolution of the corresponding concentration profiles with time, electron dispersive X-ray spectroscopy (EDX) was used and the data are reported in *Excel files*. From these concentration profiles, the widths of the diffusion affected zones for Ni and Cr were systematically determined at different temperatures and times, apparent diffusion coefficients were deduced and all these data are provided in tables. The research data reported here have a fundamental value and document the growth kinetics of σ precipitates within grains and at grain boundaries. These data may help to establish a model able to predict how the precipitation kinetics of σ particles in FCC HEAs is affected by the alloy grain size and how the microstructure (volume fraction, size and distribution of σ precipitates) evolves with time and temperature. This approach may also be extended to austenitic steels and superalloys.

**Specifications Table**

**Table:** 

Subject	Materials Science
Specific subject area	High-entropy alloys (HEAs), Phase transformation, Diffusion, Growth of precipitates, Allotriomorphs
Type of data	Micrographs (scanning electron microscopy), Tables (microstructural parameters, characteristic diffusion lengths), Excel sheets (raw EDX concentration profiles across interfaces)
How data were acquired	SEM: JEOL JSM-7200F, Quanta FEI 650 ESEM
Data format	Raw (micrographs, EDX-concentration profiles), Analyzed (dimensions of σ precipitates located at grain boundaries of the FCC matrix and within FCC grains, mean spacings between allotriomorphs, radii of curvature at the rounded edges of the allotriomorphs, widths of diffusion affected zones for different elements, apparent diffusion coefficients)
Parameters for data collection	Microstructural investigations were carried out with an SEM of type Quanta FEI 650 ESEM using a working distance of 10 mm, an acceleration voltage of 20 kV, and a spot size of 6 µm. EDX concentration profiles were recorded using an SEM of type JEOL JSM-7200F with an acceleration voltage of 15 kV, a probe current of 14 nA, and a working distance of 10 mm.
Description of data collection	Metallographic samples were cut, ground, and electropolished.
Data source location	Institute for Materials, Ruhr-University Bochum, Universitätsstr. 150, 44801 Bochum, Germany
Data accessibility	Data are with the article (attached file)
Related research article	Laplanche, Guillaume, Growth kinetics of σ-phase precipitates and underlying diffusion processes in CrMnFeCoNi high-entropy alloys. Acta Materialia 199, 193-208. https://doi.org/10.1016/j.actamat.2020.08.023

## Value of the Data

• The fundamental research datasets reported here are related to the growth kinetics of σ precipitates in an initially single-phase FCC Cr_26_Mn_20_Fe_20_Co_20_Ni_14_ (in at.%) high-entropy alloy (HEA). These data may be useful for other researchers in the community of HEAs and compositionally complex alloys (CCAs). Moreover, they constitute benchmark data that could be used to refine existing models dealing with the precipitation kinetics of TCP phases in polycrystalline FCC alloys.
• The microstructural data compilation reported here (BSE micrographs, Tables reporting the temporal evolution of precipitate dimensions at different temperatures) may be used to improve existing algorithms allowing the automated analysis of microstructures.
• The temporal evolution of concentration profiles at interfaces (FCC/σ and FCC grain boundaries) may serve as a benchmark dataset for phase-field modelling.
• Our data may be used to improve the understanding of the precipitation kinetics of TCP phases in complex engineering alloys such as superalloys and austenitic steels, *i.e.*, they could help to understand how to tune their chemistry to impede their formation thermodynamically and/or kinetically.
• The apparent diffusion coefficients reported here could be useful for other researchers working in the field of diffusion and are interested in how these coefficients are affected by chemistry and dislocation density.

## 1 Data Description

Recently, high-entropy alloys (HEAs) allowed to study several fundamental questions, *e.g.*, how can one explain solid solution strengthening in these alloys [1]? Can unusual deformation mechanisms [2], [3], [4] and elastic properties [5,6] be observed in HEAs? Can HEAs be used for electrocatalysis [7]? How does diffusion proceed in these chemically complex alloys [8,9]? Are HEAs microstructurally stable [10,11]? Even though these outstanding questions have attracted attention, the related data are rarely reported in the literature [12], [13], [14], precluding their reuse by other researchers.

The data compilation of the present article includes microstructural and compositional data related to the precipitation of σ precipitates (a brittle intermetallic with a tetragonal structure; space group *P*4_2_/*mnm*) in an initially single-phase Cr_26_Mn_20_Fe_20_Co_20_Ni_14_ HEA (composition in at.%) with an average grain size of 50 µm. Anneals between 600 °C and 1000 °C for times from 0.05 h to 1000 h followed by air cooling were performed to study the growth kinetics of σ precipitates that formed within grains and at grain boundaries.

BSE micrographs of the alloy aged at different temperatures and times can be found in the folder “BSE_microstructures” of the supplementary *ZIP fil*e. In this folder, each subfolder contains four BSE images provided as *Tiff files* that were recorded with a magnification of 1000 at different locations on the specimen. The names of the subfolders show the heat treatments that were performed prior to imaging, *e.g.*, the name of the subfolder “0700C_0100h” indicates that the alloy was annealed at 700 °C for 100 h. This system of notations was also employed for the other folders contained in the *ZIP file*. The BSE micrographs were used to determine the size of the five largest precipitates, which were observed within grains. The temporal evolution of the mean radius of these precipitates at different temperatures is shown in Table 1 of the present article and these data are plotted in Fig. 6 of Ref. [15].

**Table 1:** Temporal evolution of the radius (in µm) of spherical particles observed within the grains of the FCC matrix of a Cr_26_Mn_20_Fe_20_Co_20_Ni_14_ alloy between 700°C and 1000°C. Numbers in parentheses are the standard deviations of the last digit.

***time* (h)**	**700°C**	**800°C**	**900°C**	**1000°C**
**0.05**	-	-	0.10(1)	0.28(8)
**0.1**	-	-	0.26(1)	0.23(2)
**1**	-	0.24(2)	0.41(6)	0.6(1)
**10**	-	0.35(7)	1.04(3)	1.0(1)
**100**	0.49(5)	1.9(2)	2.3(1)	1.5(3)
**500**	0.91(6)	1.9(1)	1.7(2)	1.8(1)
**1000**	1.03(9)	1.9(1)	3.1(2)	1.7(4)

The shape of the allotriomorphs forming at grain boundaries is approximated to that of thick disks with a mean half-length, *l*_GB_, along the grain boundary, an average half-thickness, *th*_GB_, perpendicular to the grain boundary, and a mean radius of curvature at their rounded edges, *r*_GB_, as illustrated in Fig. 1a of Ref. [15]. The temporal evolutions of *l*_GB_ and *th*_GB_ at different temperatures are shown in Tables 2 and 3, respectively, of the present article and are plotted in Fig. 7 of Ref. [15]. The allotriomorphs are either faceted or rounded at their edges. The evolution of the radii of curvature of faceted and rounded σ allotriomorphs at their edges are provided in Tables 4 and 5, respectively, and these data are represented in Fig. S4b (supplementary materials of Ref. [15]). Finally, the mean spacing between allotriomorphs along the grain boundary is given in Table 6 of the present paper and can be visualized in Fig. S4a of Ref. [15].

**Table 2:** Temporal evolution of the half-length, *l*_GB_ (in µm), of σ particles located at FCC grain boundaries of a Cr_26_Mn_20_Fe_20_Co_20_Ni_14_ alloy between 600 °C and 1000 °C. Numbers in parentheses are the standard deviations of the last digit.

***time* (h)**	**600 °C**	**700 °C**	**800 °C**	**900 °C**	**1000 °C**
**0.05**	-	-	0.4(2)	0.66(9)	0.56(6)
**0.1**	-	-	0.48(7)	0.95(9)	0.9(1)
**1**	-	0.50(9)	1.0(1)	1.7(2)	2.1(2)
**10**	-	1.09(9)	1.9(4)	2.2(2)	3.2(3)
**100**	1.0(2)	1.75(7)	3.2(1)	2.8(3)	4.3(3)
**500**	1.26(9)	2.8(2)	3.6(2)	3.9(3)	3.8(4)
**1000**	1.6(1)	2.8(3)	3.5(2)	3.4(4)	4.3(6)

**Table 3:** Temporal evolution of the half-thickness, *th*_GB_ (in µm), of σ allotriomorphs in a Cr_26_Mn_20_Fe_20_Co_20_Ni_14_ alloy between 600 °C and 1000 °C. Numbers in parentheses are the standard deviations of the last digit.

***time* (h)**	**600 °C**	**700 °C**	**800 °C**	**900 °C**	**1000 °C**
**0.05**	-	-	0.08(4)	0.25(3)	0.20(4)
**0.1**	-	0.057(3)	0.09(2)	0.27(4)	0.33(8)
**1**	-	0.15(3)	0.33(6)	0.7(1)	1.1(1)
**10**	-	0.54(6)	0.7(1)	1.5(2)	1.6(3)
**100**	0.34(5)	0.77(9)	1.5(3)	1.9(3)	2.2(3)
**500**	0.60(6)	1.1(2)	1.9(3)	2.3(3)	2.0(2)
**1000**	0.62(5)	1.3(2)	2.3(1)	2.1(2)	2.1(2)

**Table 4:** Temporal evolution of the radius of curvature of *faceted* σ allotriomorphs of a Cr_26_Mn_20_Fe_20_Co_20_Ni_14_ alloy between 600 °C and 1000 °C. Numbers in parentheses are the standard deviations of the last digit.

***time* (h)**	**600 °C**	**700 °C**	**800 °C**	**900 °C**	**1000 °C**
**0.05**	-	-	1(1)	1.0(4)	0.9(4)
**0.1**	-	-	1.3(8)	1.8(7)	1.5(8)
**1**	0.7(2)	0.9(5)	1.7(8)	2.5(9)	2.6(8)
**10**	0.7(3)	1.4(4)	3(2)	2.3(9)	4(2)
**100**	1.7(8)	2.4(5)	4(2)	3(1)	5(2)
**500**	1.6(4)	4(2)	4.3(9)	4.5(9)	5(2)
**1000**	2.5(6)	4(1)	3.8(6)	5(1)	5(2)

**Table 5:** Temporal evolution of the radius of curvature of *rounded* σ allotriomorphs in a Cr_26_Mn_20_Fe_20_Co_20_Ni_14_ alloy between 600 °C and 1000 °C. Numbers in parentheses are the standard deviations of the last digit.

***time* (h)**	**600 °C**	**700 °C**	**800 °C**	**900 °C**	**1000 °C**
**0.05**	-	-	-	0.17(5)	0.15(5)
**0.1**	-	-	0.05(1)	0.12(3)	0.15(6)
**1**	0.13(3)	0.10(2)	0.23(9)	0.19(5)	0.3(1)
**10**	0.13(3)	0.17(4)	0.3(2)	0.3(2)	0.28(9)
**100**	0.15(3)	0.23(9)	0.4(4)	0.3(1)	0.5(2)
**500**	0.15(3)	0.3(1)	0.4(3)	0.5(2)	0.5(5)
**1000**	0.16(6)	0.23(8)	0.5(3)	0.5(2)	0.6(2)

**Table 6:** Evolution with time of the mean spacing between σ allotriomorphs along grain boundaries in a Cr_26_Mn_20_Fe_20_Co_20_Ni_14_ HEA between 700 °C and 1000 °C. Numbers in parentheses are the standard deviations of the last digit.

***time* (h)**	**700 °C**	**800 °C**	**900 °C**	**1000 °C**
**0.1**	-	-	-	42(7)
**1**	32(8)	32(8)	13(2)	10.5(5)
**10**	11(2)	11(3)	7.1(5)	10.2(5)
**100**	9.2(8)	7.4(3)	7.2(4)	12.2(4)
**500**	7.9(7)	8(1)	7.8(7)	13(2)
**1000**	5.9(8)	6.1(4)	8.6(4)	17(4)

The concentration profiles, forming at interfaces and resulting from the precipitation of the σ phase, were recorded by EDX perpendicularly to interfaces (FCC/σ interfaces and FCC grain boundaries) after different anneals. The *Excel files* containing the corresponding concentration profiles obtained across FCC/σ interfaces and FCC grain boundaries can be found in the *ZIP file* under the “EDX_line_scan_FCC-sigma”- and “EDX_line_scan_GB”-folders, respectively. For example, the *Excel file* named “0700C_1000h_GB” was recorded at grain boundaries (GB) in an alloy annealed at 700 °C for 1000 h. This *Excel file* has five sheets labelled from “GB1” to “GB5” representing individual datasets recorded at different locations. In each *Excel sheet*, the distances (in µm) between the first point of the EDX line scan and the subsequent points are listed in column A. The corresponding local concentrations in Cr, Mn, Fe, Co, and Ni are provided in at.% in columns B to F, respectively. As another example, the *Excel file* “1000C_00.1h_FCC-sigma” comprises five EDX line scans (five sheets named from “FS1” to “FS5”, where “FS” stems for FCC/sigma) that were recorded at five distinct FCC/σ interfaces in an alloy annealed at 1000 °C for 0.1 h (6 min). One end of each EDX line scan was always located far away from σ particles and FCC grain boundaries. At this extremity, the measured FCC composition should reflect the instantaneous composition of the FCC matrix and the values obtained at different times and temperatures are given in Table 7 of the present manuscript and a plot of the instantaneous Cr-concentration in the FCC matrix can be found in Fig. 4 of the original research article [15].

**Table 7:** Evolution with time of the Cr-concentration ($x_{m}^{\infty }$in at.%) in the FCC matrix far away from σ particles. EDX, for which the absolute experimental error is ∼1.0 at.%, was used for the measurements.

***time* (h)**	**600 °C**	**700 °C**	**800 °C**	**900 °C**	**1000 °C**
**0.1**	26.0	26.0	26.0	26.0	25.8
**1**	26.0	26.0	26.0	25.6	25.7
**10**	26.0	26.0	25.9	25.5	25.2
**100**	26.0	25.9	25.6	22.4	24.4
**500**	26.0	25.8	23.3	21.9	24.4
**1000**	26.0	25.8	20.1	21.9	24.4

From the concentration profiles, the characteristic widths of diffusion affected zones that form at σ/FCC interfaces and FCC grain boundaries were determined using the methodology illustrated in Figs. 3a,b of Ref. [15]. The corresponding data are respectively reported in Tables 8 and 9 for Cr and Tables 10 and 11 for Ni of the present article and a representation of these data is provided in Fig. 5 of Ref. [15]. Note that the concentration gradients for Mn, Fe, and Co were too small to accurately follow their evolutions with time.

**Table 8:** Evolution of the widths (in µm) of diffusion affected zones, *W*_DAZ_, for Cr across σ/FCC with time and temperature. Numbers in parentheses are the standard deviations of the last digit.

***time* (h)**	**700 °C**	**800 °C**	**900 °C**	**1000 °C**
**0.1**	-	-	-	1.3(7)
**1**	-	-	1.2(7)	3.5(8)
**10**	-	1.0(7)	2.8(7)	8(3)
**100**	0.9(7)	2.1(7)	-	-
**500**	1.2(7)	3.9(9)	-	-
**1000**	2.5(9)	-	-	-

**Table 9:** Evolution of the widths (in µm) of diffusion affected zones, *W*_DAZ_, for Cr across FCC grain boundaries with time and temperature. Numbers in parentheses are the standard deviations of the last digit.

***time* (h)**	**600 °C**	**700 °C**	**800 °C**	**900 °C**	**1000 °C**
**0.1**	-	-	-	0.5(4)	0.9(4)
**1**	-	-	0.4(1)	1.2(5)	2.4(3)
**10**	-	-	1.0(2)	2.4(4)	9(3)
**100**	-	0.7(4)	1.7(4)	-	-
**500**	0.31(4)	1.4(4)	3.6(8)	-	-
**1000**	0.38(4)	2.0(4)	-	-	-

**Table 10:** Evolution of the widths (in µm) of diffusion affected zones, *W*_DAZ_, for Ni across σ/FCC with time and temperature. Numbers in parentheses are the standard deviations of the last digit.

***time* (h)**	**800 °C**	**900 °C**
**0.1**	-	-
**1**	-	0.8(4)
**10**	-	1.7(3)
**100**	1.0(2)	-
**500**	2.1(3)	-
**1000**	-	-

**Table 11:** Evolution of the widths (in µm) of diffusion affected zones, *W*_DAZ_, for Ni across FCC grain boundaries with time and temperature. Numbers in parentheses are the standard deviations of the last digit.

***time* (h)**	**600 °C**	**700 °C**	**800 °C**	**900 °C**	**1000 °C**
**0.1**	-	-	-	-	0.6(2)
**1**	-	-	-	0.8(4)	1.4(4)
**10**	-	-	-	1.5(3)	-
**100**	-	0.35(3)	1.02(7)	-	-
**500**	0.19(3)	0.7(1)	2.4(4)	-	-
**1000**	0.24(5)	0.95(9)	-	-	-

Apparent diffusion coefficients between 600 °C and 1000 °C were deduced from the widening kinetics of the diffusion affected zones provided in Tables 8–11 using *W*_DAZ_ ≈ 2 (*D t*)^1/2^ where *W*_DAZ_ is the width of the diffusion affected zone after annealing for a time *t* and *D* is the apparent diffusion coefficient. The values obtained for Cr and Ni are reported in Table 12 of the present data in Brief article and these data are plotted in Fig. 8a of Ref. [15]. Apparent diffusion coefficients, listed in Table 13, were obtained from the growth kinetics of σ particles observed within grains of the FCC matrix using Eq. (2) of Ref. [15] and the corresponding data are shown as blue data points in Fig. 8b of Ref. [15]. From the lengthening rate of σ allotriomorphs, apparent diffusion coefficients were calculated using Eqs. (5 - 6) of Ref. [15] and the data are shown in Table 14 of the present article and represented as red data points in Fig. 8b of Ref. [15].

**Table 12:** Apparent diffusion coefficients in m^2^/s determined from the evolution with time of concentration profiles of Ni and Cr at interfaces. Numbers in parentheses are the standard deviations of the last digit.

***Element***	**600 °C**	**700 °C**	**800 °C**	**900 °C**	**1000 °C**
***Ni***	4.3(2) × 10^−21^	7.2(4) × 10^−20^	7.2(1) × 10^−19^	1.8(2) × 10^−17^	1.6(2) × 10^−16^
***Cr***	10(2) × 10^−21^	2.8(7) × 10^−19^	2.4(5) × 10^−18^	5(1) × 10^−17^	4.7(9) × 10^−16^

**Table 13:** Apparent diffusion coefficients in m^2^/s obtained from the temporal evolutions of the radius of the five largest σ precipitates observed within grains of the FCC matrix. Numbers in parentheses are the standard deviations of the last digit.

**600 °C**	**700 °C**	**800 °C**	**900 °C**	**1000 °C**
-	2.6(3) × 10^−19^	4.3(7) × 10^−18^	4.9(3) × 10^−17^	3.0(4) × 10^−16^

**Table 14:** Apparent diffusion coefficients in m^2^/s obtained from the lengthening rates of σ allotriomorphs at different temperatures. Numbers in parentheses are the standard deviations of the last digit.

**600 °C**	**700 °C**	**800 °C**	**900 °C**	**1000 °C**
9.5(4) × 10^−21^	1.9(1) × 10^−19^	2.9(1) × 10^−18^	3.2(3) × 10^−17^	7.2(4) × 10^−16^

## 2 Experimental Design, Materials, and Methods

The Cr_26_Mn_20_Fe_20_Co_20_Ni_14_ (in at.%) HEA was solution annealed at 1080 °C for 15 min followed by air cooling resulting in a single-phase FCC alloy with a mean grain size of ∼50 µm. Small pieces (∼8 × 8 × 2 mm^3^) were subsequently annealed in evacuated quartz tubes at temperatures between 600 °C and 1000 °C for times up to 1000 h followed by air cooling. These pieces were then prepared metallographically and electropolished following the procedure reported in [10] and the microstructures were imaged with the backscattered electron (BSE) contrast in a scanning electron microscope (Quanta FEI 650 ESEM). Four BSE micrographs for each heat treatment were recorded using an acceleration voltage of 20 kV, a working distance of 10 mm, and a spot size of 6 µm (these micrographs can be found in the *ZIP file* attached to this paper). This allowed to follow the growth kinetics of σ precipitates observed within grains of the FCC matrix and those located at the grain boundaries. For each BSE micrograph, the radii of the five largest precipitates observed within grains were measured resulting in a total of 20 measurements (four BSE micrographs × five precipitates). Despite the fact that some of the σ precipitates observed within grains showed some facets, they were treated as spherical and their mean radii are provided in Table 1. The dimensions of the allotriomorphs were determined using a similar procedure. Their mean half-length (*l*_GB_) and average half-thickness (*th*_GB_) are reported in Tables 2 and 3, respectively. The radii of curvature of faceted and rounded σ allotriomorphs at their edges and the mean spacings between allotriomorphs along grain boundaries are given in Tables 4–6, respectively.

Concentration profiles resulting from σ-phase precipitation in an FCC matrix were recorded across interfaces (σ/FCC interfaces and FCC grain boundaries) after different heat treatments by energy-dispersive X-ray (EDX) spectroscopy using a detector of type Oxford X-Max 80 SDD in a JEOL JSM-7200F SEM at an acceleration voltage of 15 kV, a probe current of 14 nA, and a working distance of 10 mm. EDX point analyses were carried out at discrete points (5 s each) along test lines made at right angle to grain boundaries and σ/FCC interfaces using a step size in the range [0.04 - 0.2 µm] depending on the width of the diffusion affected zones in the vicinity of these boundaries. For each heat treatment, at least five EDX concentration profiles were recorded at FCC/σ interfaces as well as FCC grain boundaries and the corresponding data can be found in the *ZIP file* attached to this paper. The EDX experiments were performed such that at least one extremity of each concentration profile was located in the FCC phase as far as possible from σ particles and grain boundaries. Thus, the FCC composition reflecting the instantaneous solute concentration, $x_{m}^{\infty }$, in the FCC matrix far away from precipitates was measured and the corresponding $x_{m}^{\infty }$-values for Cr are provided in Table 7. For σ/FCC interfaces, the largest σ particles were systematically selected to record concentration profiles with large concentration gradients. The temporal evolution of the width of these concentration profiles at σ/FCC interfaces for different temperatures are provided in Tables 8and 10 for Cr and Ni, respectively. For the grain boundaries of the FCC matrix, the line scans were performed between two allotriomorphs as close to each other as possible to maximize solute depletion at the boundary, but further apart than the electron penetration depth such that the probed volumes do not contain any σ phase. The characteristic widths of these concentration profiles for Cr and Ni after different heat treatments are given in Tables 9 and 11, respectively.

Using the temporal evolution of the width of the diffusion affected zones (*W*_DAZ_) given in Tables 8–11 for different temperatures, apparent diffusion coefficients (*D*) for Cr and Ni were deduced using the following equation of Ref. [15]: *W*_DAZ_ ≈ 2 (*D t*)^1/2^ where *t* is the annealing time. These apparent diffusion coefficients for Cr and Ni are provided in Table 12 of the present data in Brief article. The growth kinetics of σ particles in combination with Eq. (2) of Ref. [15] were used to compute the apparent diffusion coefficients listed in Table 13. Finally, apparent diffusion coefficients were also deduced from the lengthening rate of σ allotriomorphs with Eqs. (5) and (6) of Ref. [15] and the data are shown in Table 14 of the present article.

## Declaration of Competing Interest

The author declares that he has no known competing financial interests or personal relationships, which have, or could be perceived to have, influenced the work reported in this article.

## Appendix Supplementary materials

Image, application 1
