# Supplementary figures and images for: Data related to the growth of σ-phase precipitates in CrMnFeCoNi high-entropy alloys: Temporal evolutions of precipitate dimensions and concentration profiles at interfaces
Source: Data Brief. 2020 Oct 21;33:106449. doi: 10.1016/j.dib.2020.106449 (PMC7607391; doi:10.1016/j.dib.2020.106449)

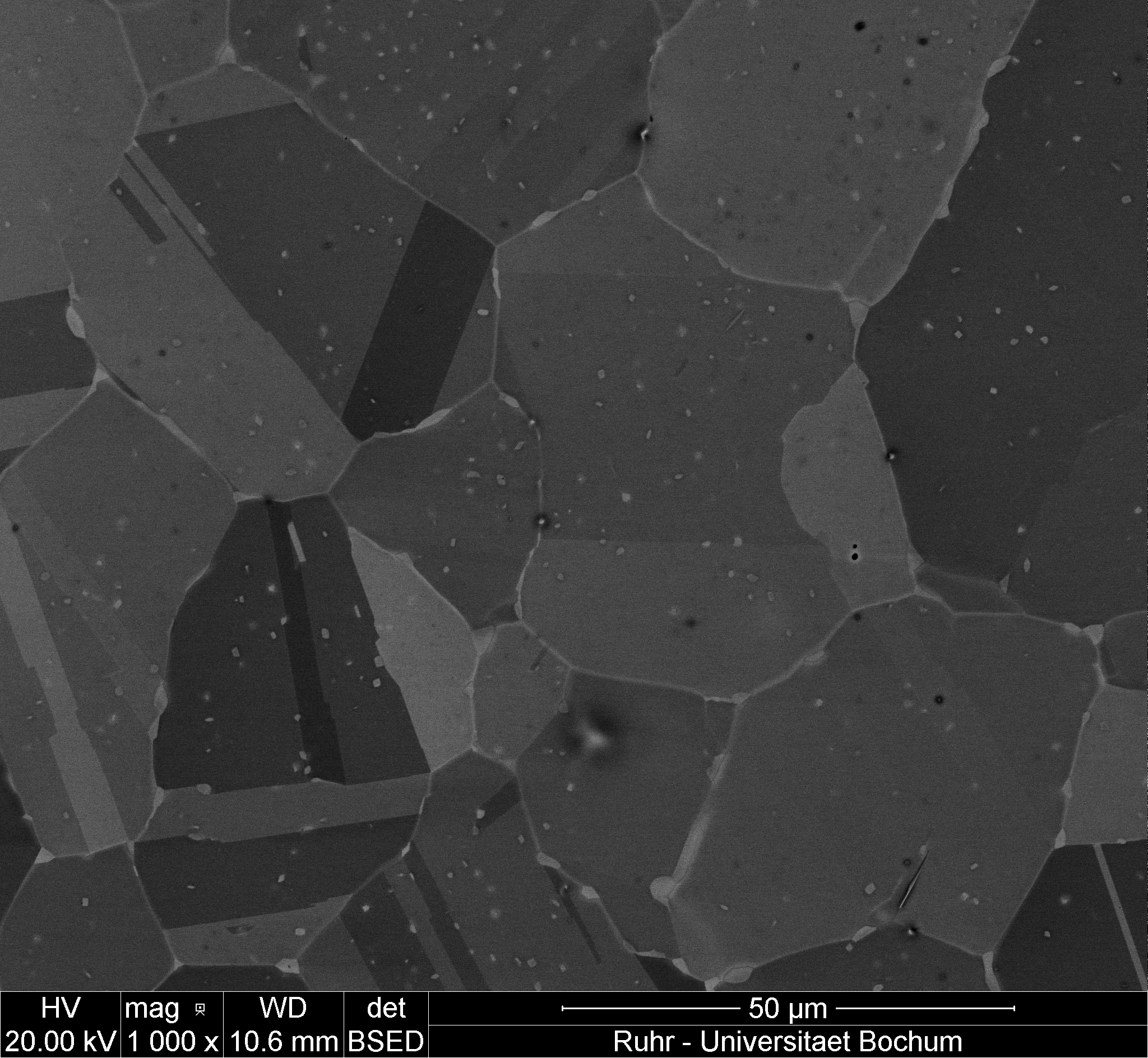

Supplement: Supplementary file 1 [file mmc1.zip › Upload_Data_in_Brief/BSE_microstructures/0700C_0100h/0700C_0100h_area1.tif]

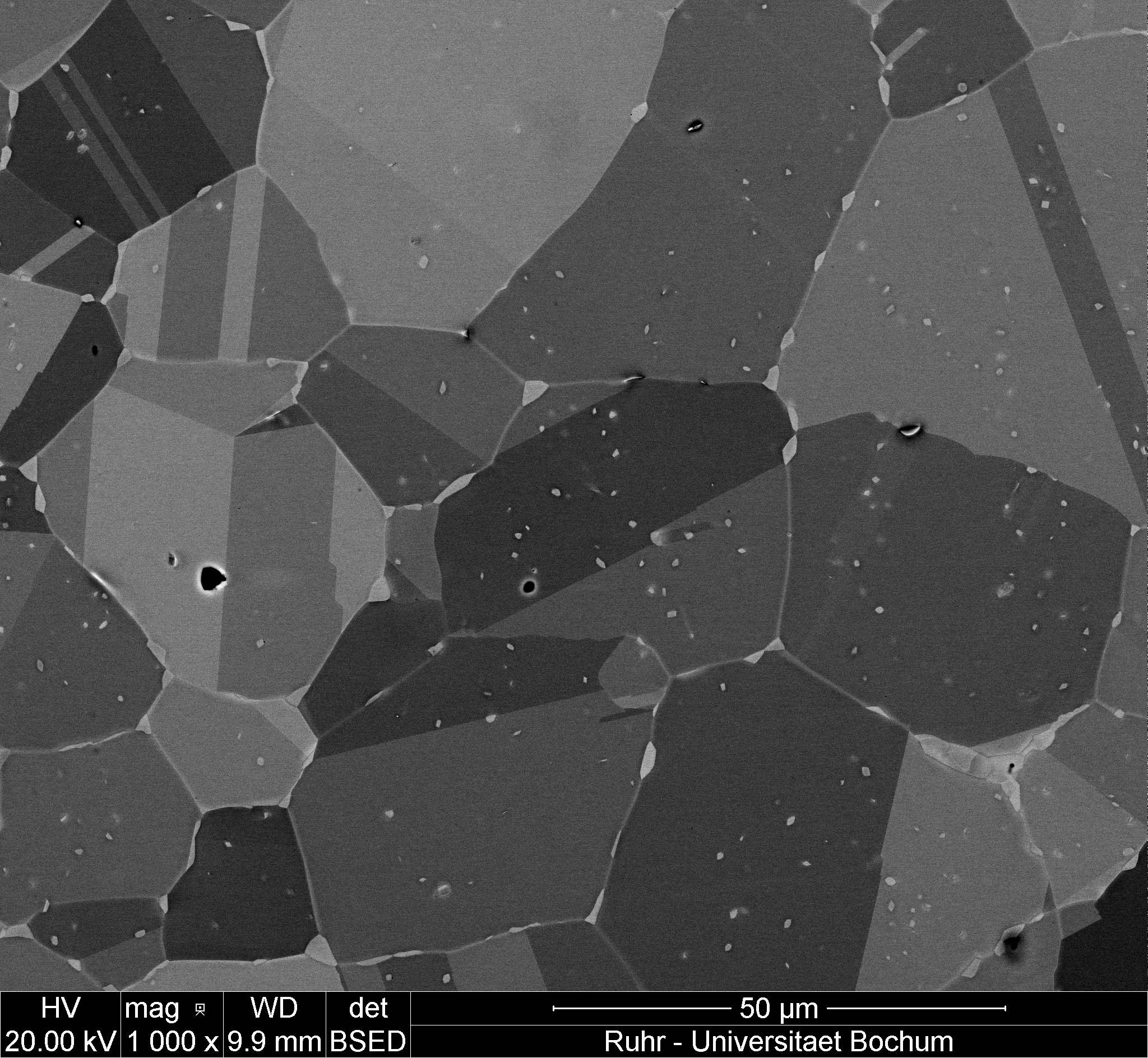

Supplement: Supplementary file 1 [file mmc1.zip › Upload_Data_in_Brief/BSE_microstructures/0700C_0100h/0700C_0100h_area5.tif]

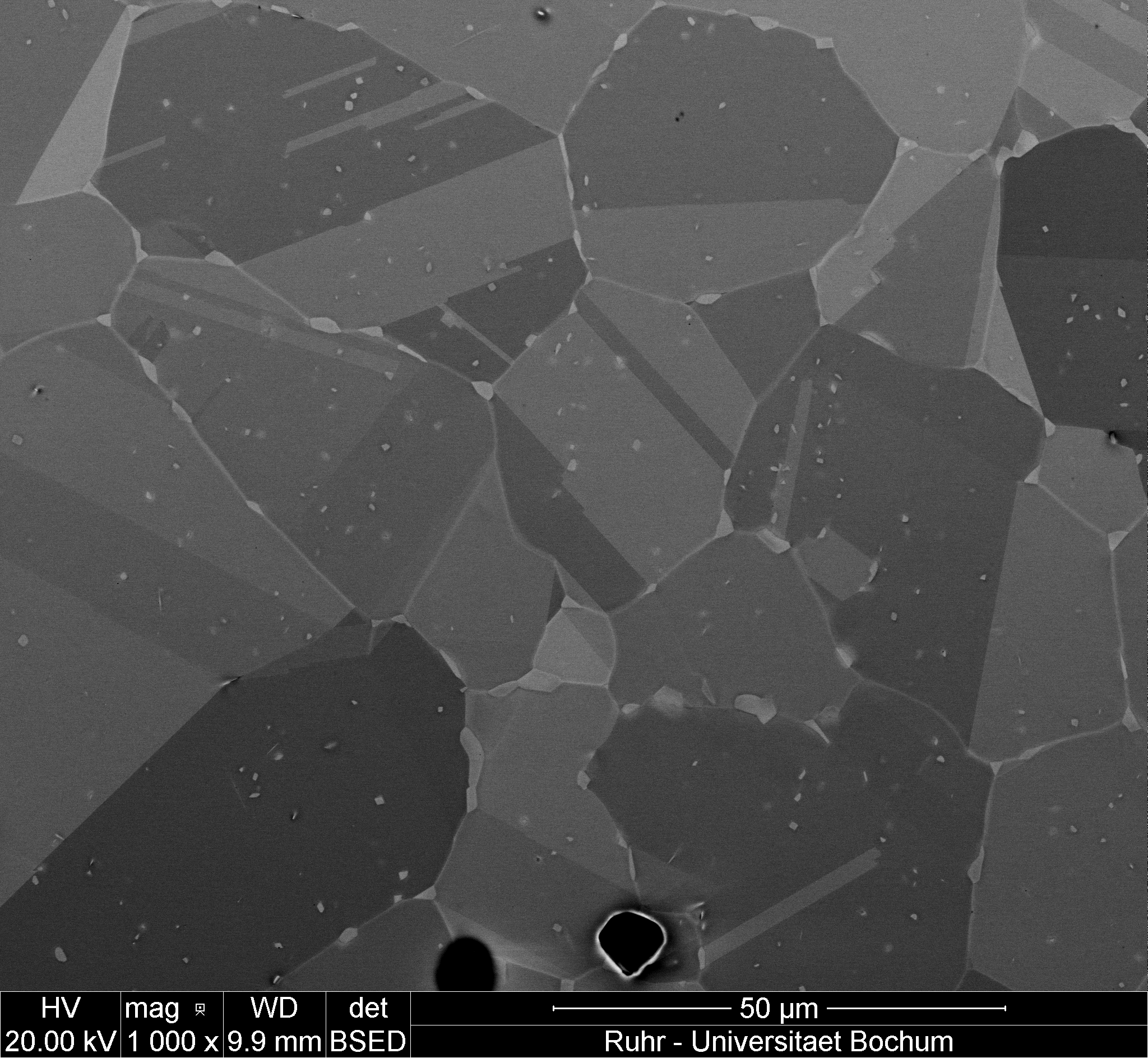

Supplement: Supplementary file 1 [file mmc1.zip › Upload_Data_in_Brief/BSE_microstructures/0700C_0100h/0700C_0100h_area7.tif]

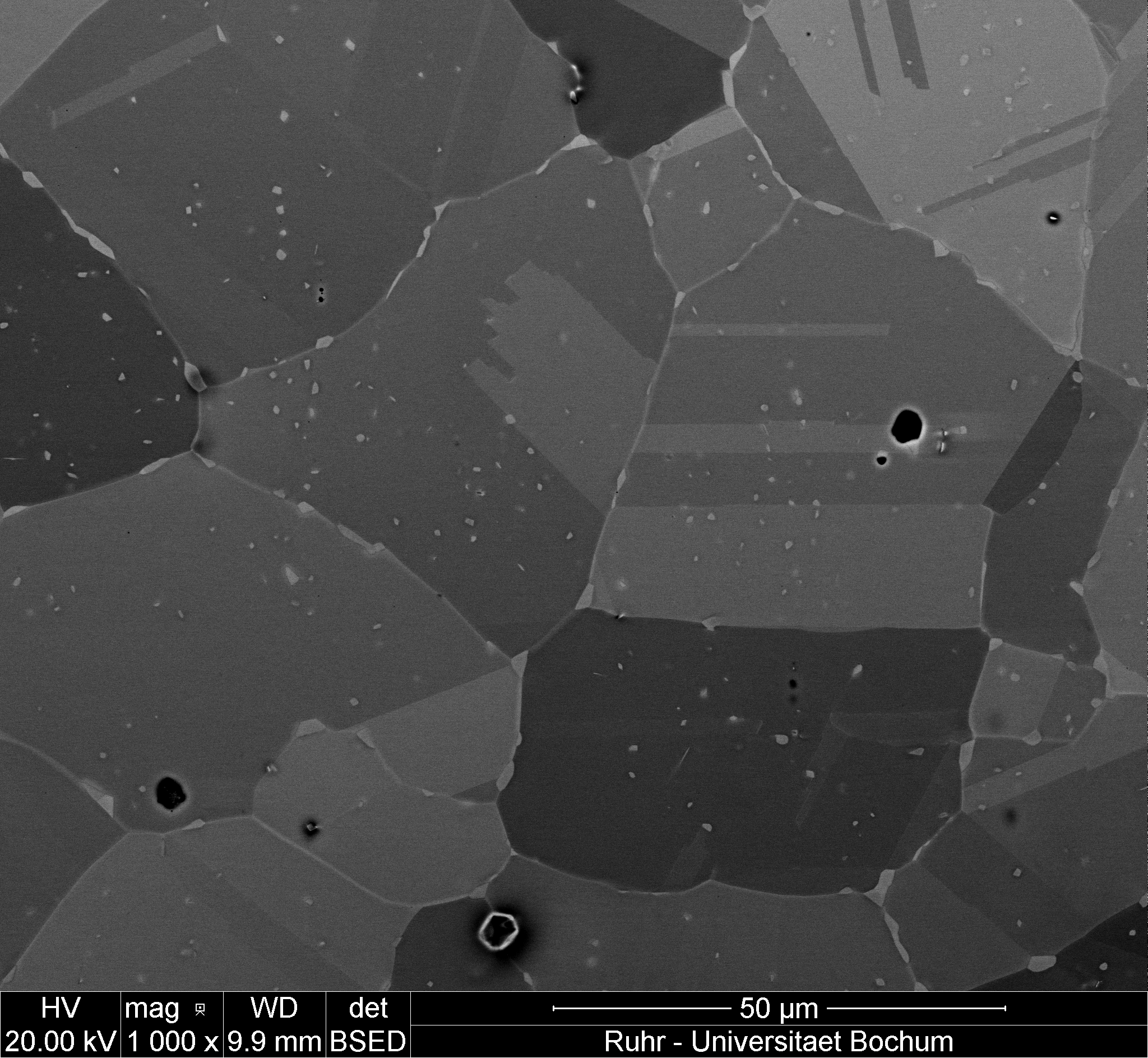

Supplement: Supplementary file 1 [file mmc1.zip › Upload_Data_in_Brief/BSE_microstructures/0700C_0100h/0700C_0100h_area8.tif]

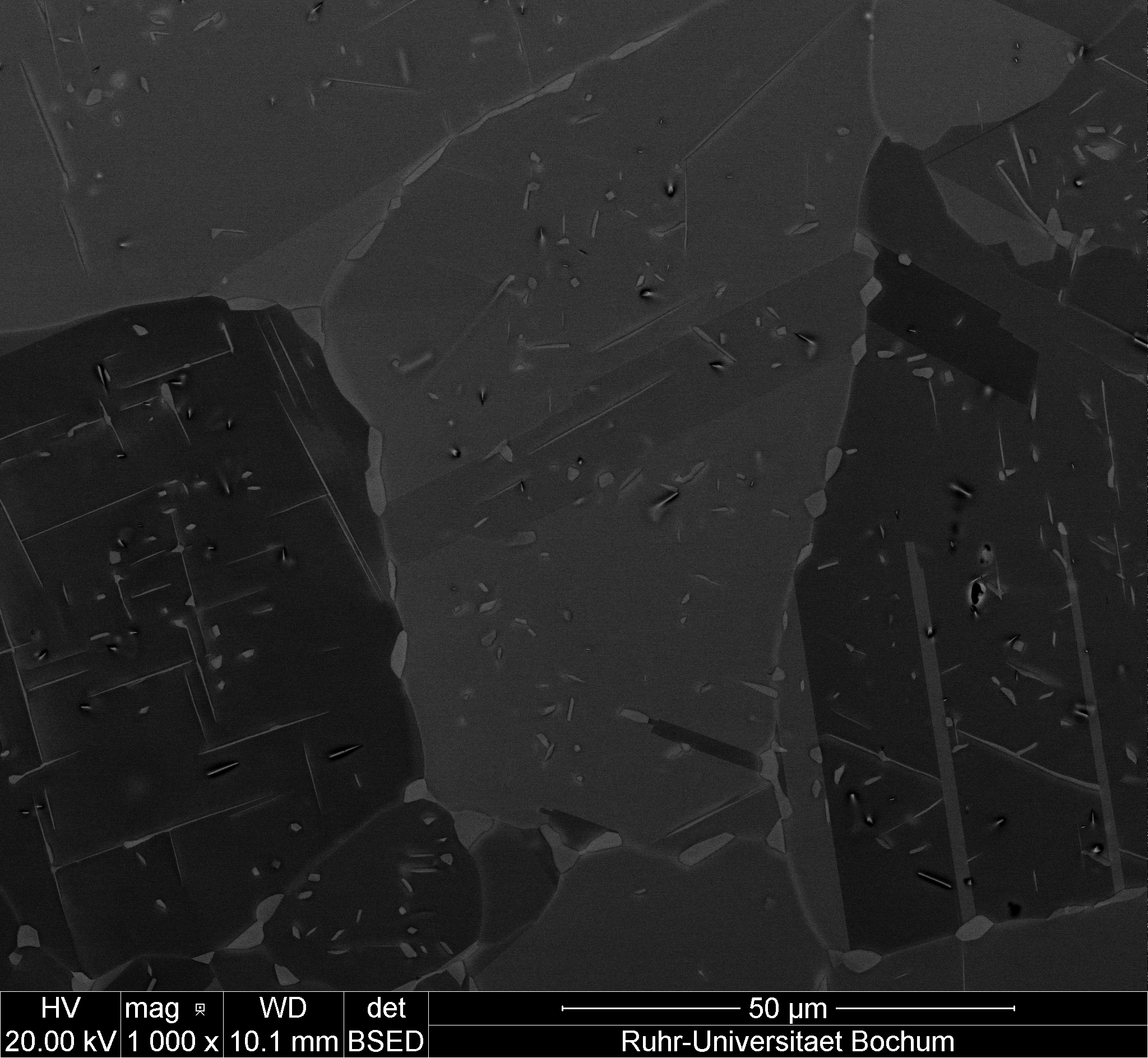

Supplement: Supplementary file 1 [file mmc1.zip › Upload_Data_in_Brief/BSE_microstructures/0700C_0500h/0700C_0500h_area1.tif]

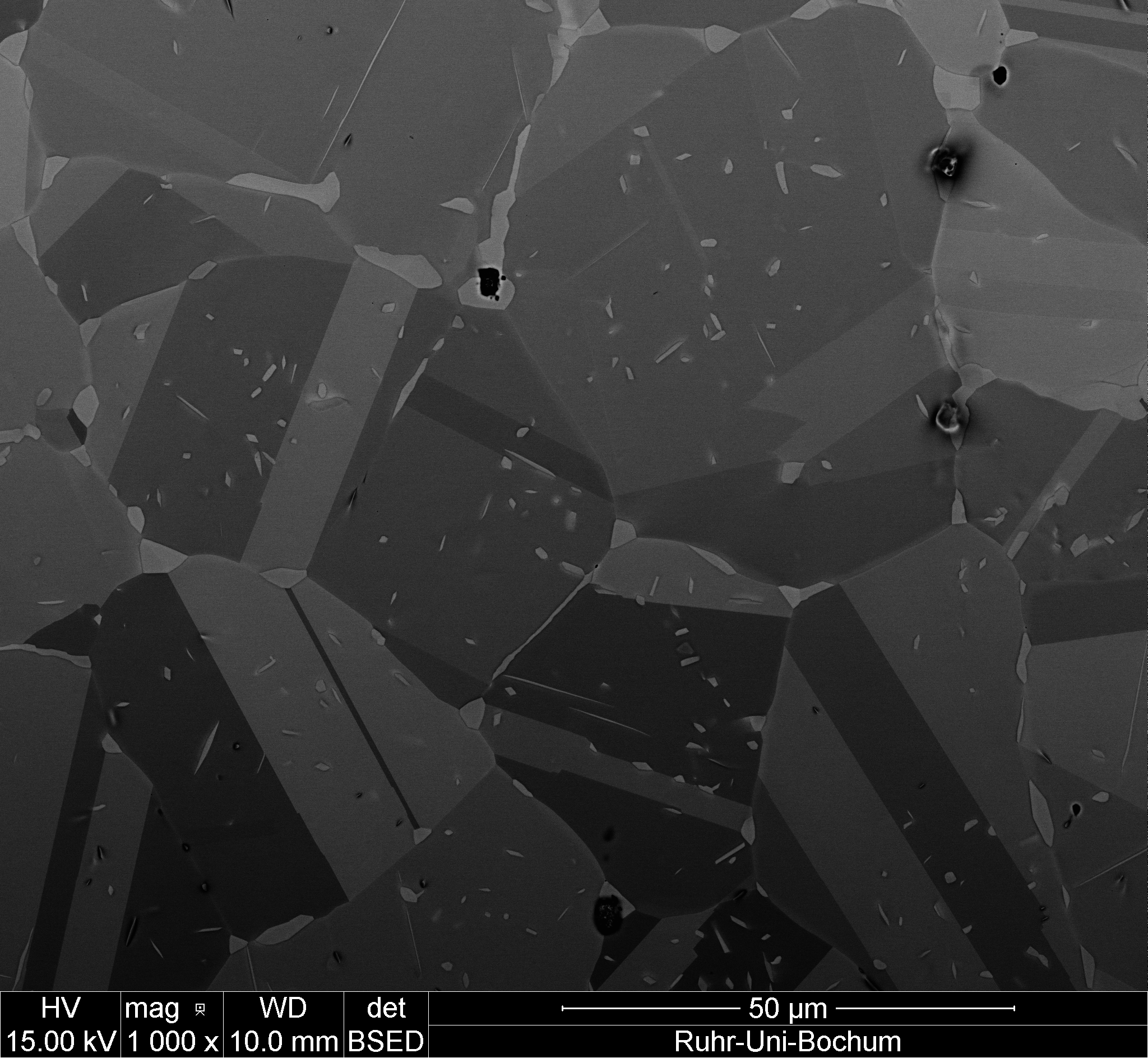

Supplement: Supplementary file 1 [file mmc1.zip › Upload_Data_in_Brief/BSE_microstructures/0700C_0500h/0700C_0500h_area2.tif]

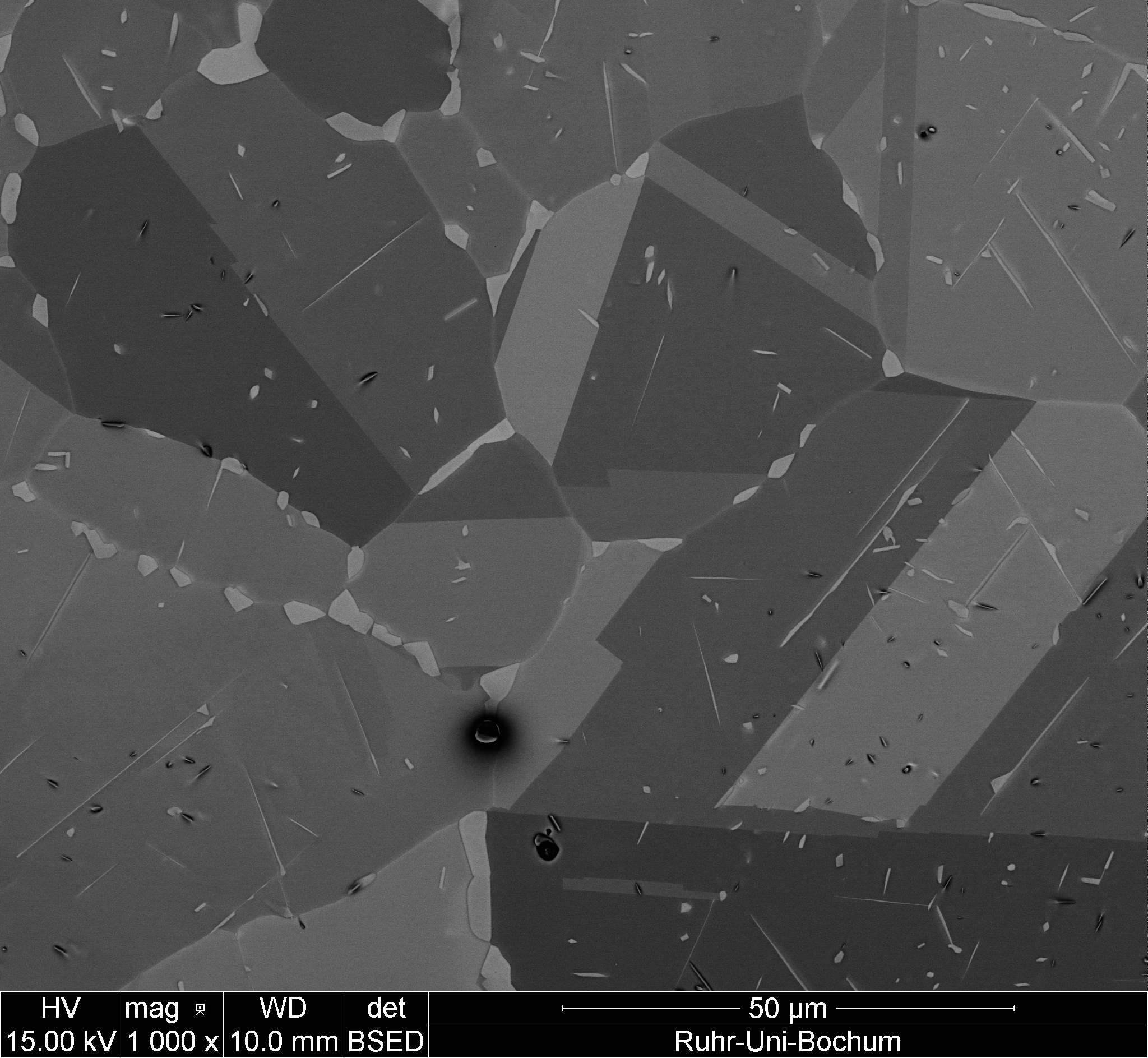

Supplement: Supplementary file 1 [file mmc1.zip › Upload_Data_in_Brief/BSE_microstructures/0700C_0500h/0700C_0500h_area3.tif]

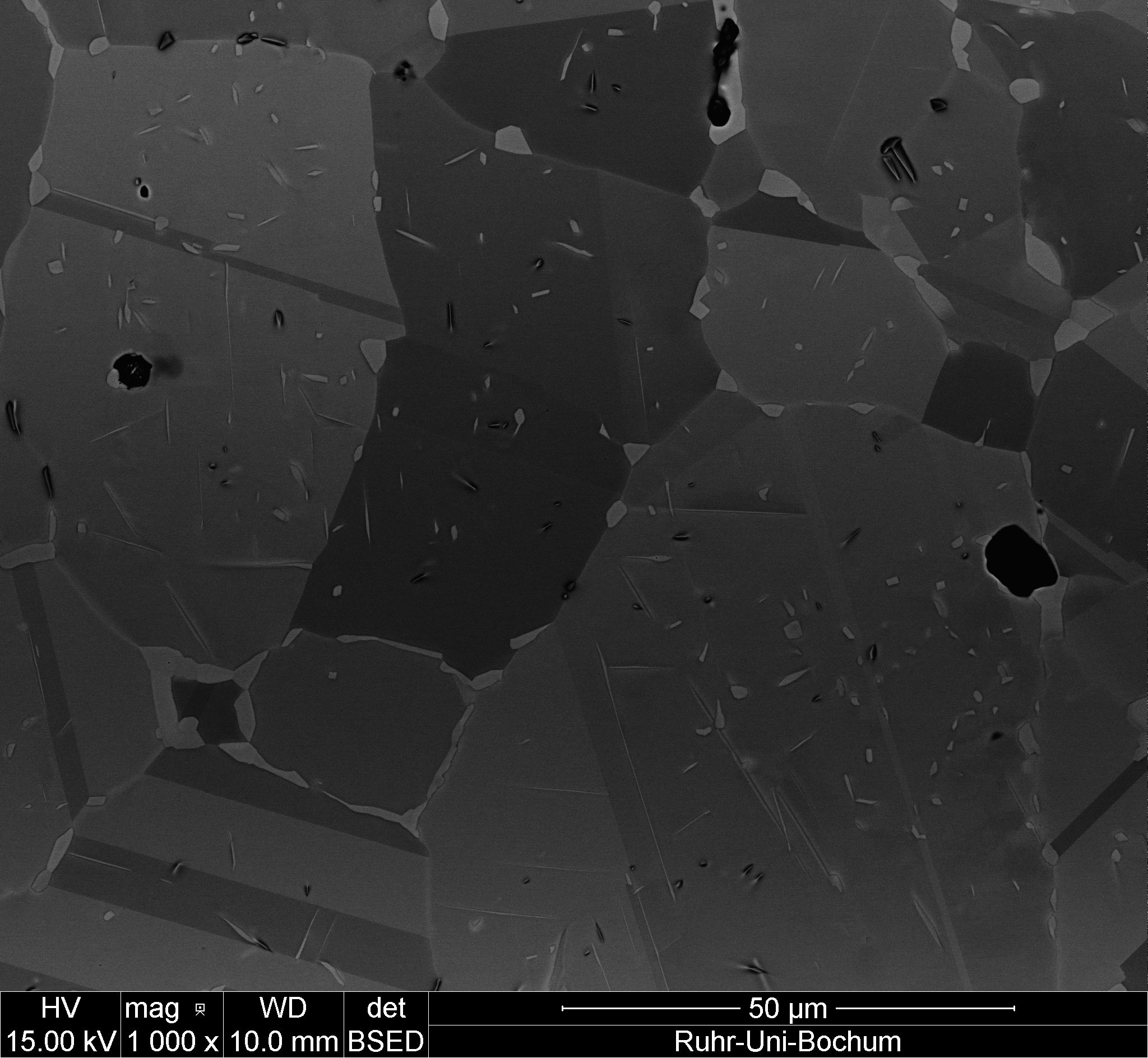

Supplement: Supplementary file 1 [file mmc1.zip › Upload_Data_in_Brief/BSE_microstructures/0700C_0500h/0700C_0500h_area4.tif]

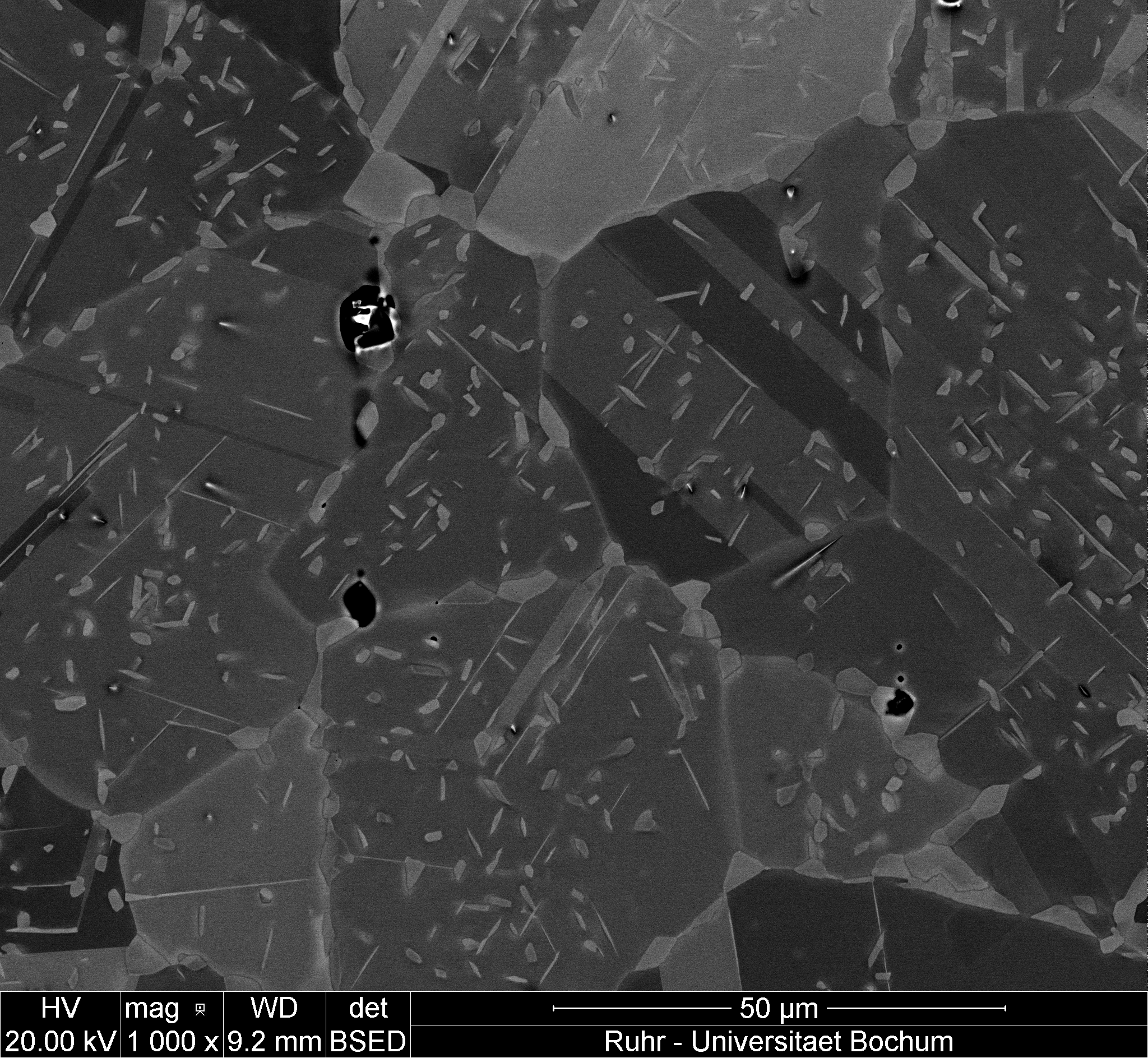

Supplement: Supplementary file 1 [file mmc1.zip › Upload_Data_in_Brief/BSE_microstructures/0700C_1000h/0700C_1000h_area1.tif]

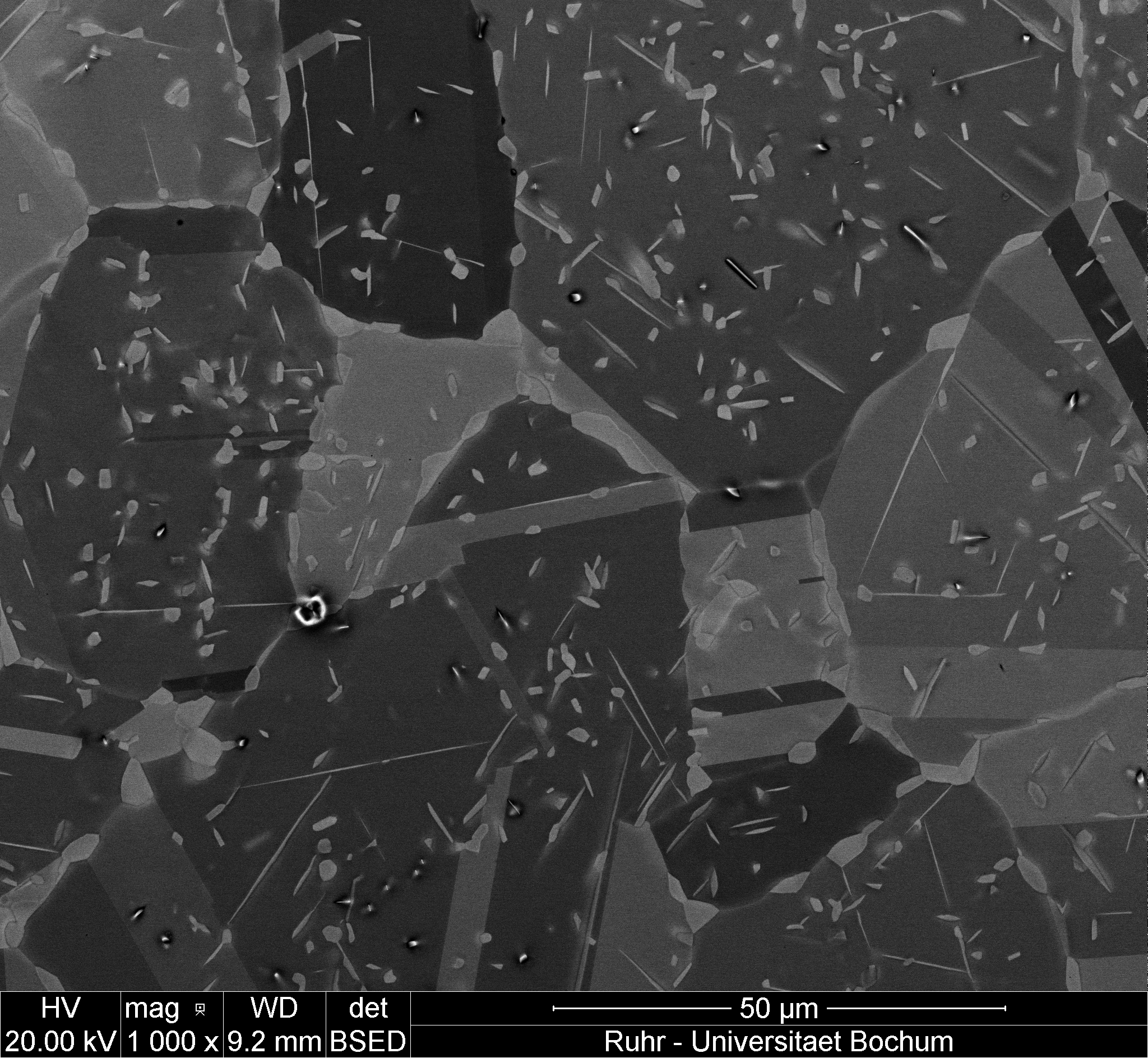

Supplement: Supplementary file 1 [file mmc1.zip › Upload_Data_in_Brief/BSE_microstructures/0700C_1000h/0700C_1000h_area2.tif]

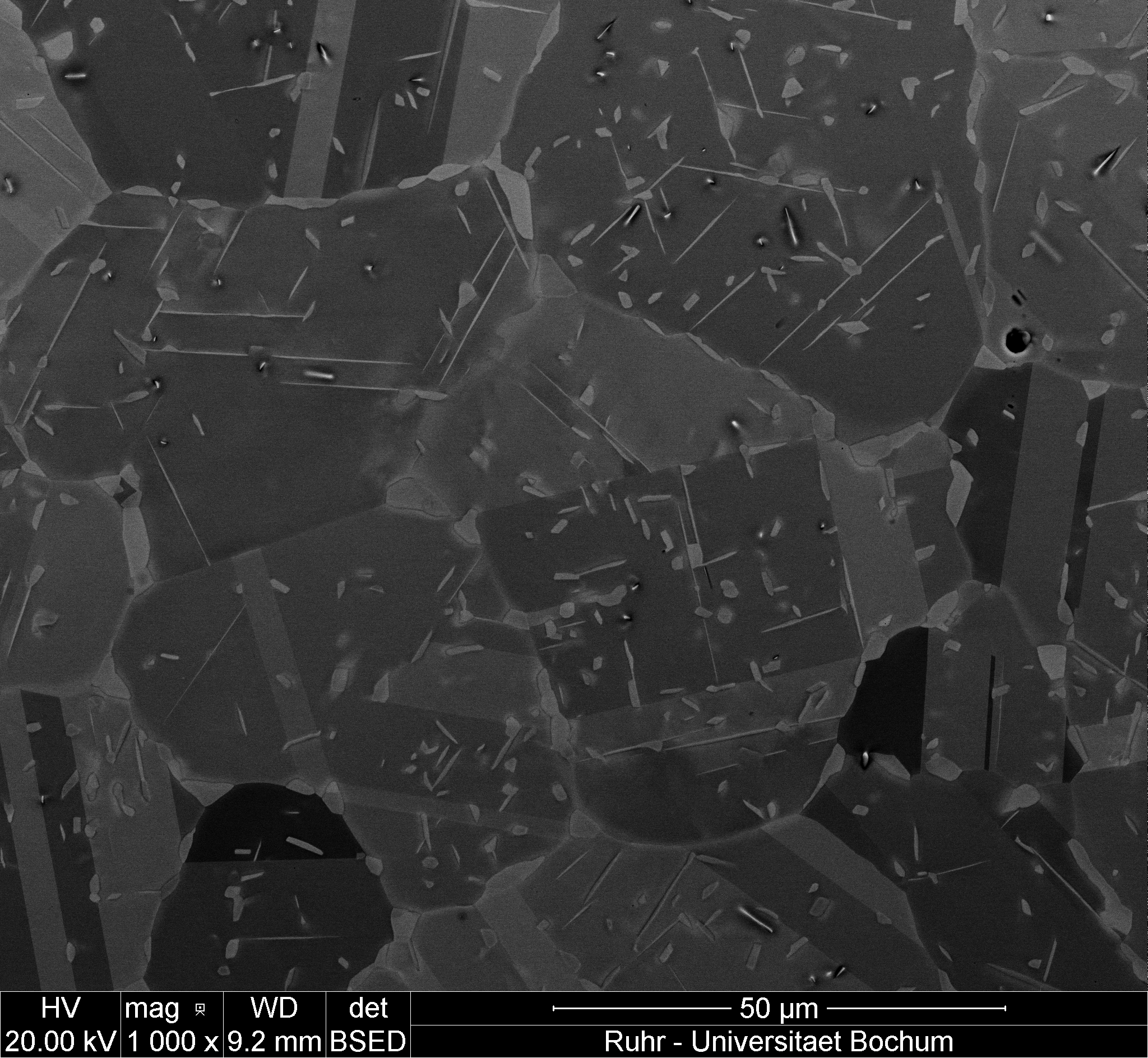

Supplement: Supplementary file 1 [file mmc1.zip › Upload_Data_in_Brief/BSE_microstructures/0700C_1000h/0700C_1000h_area3.tif]

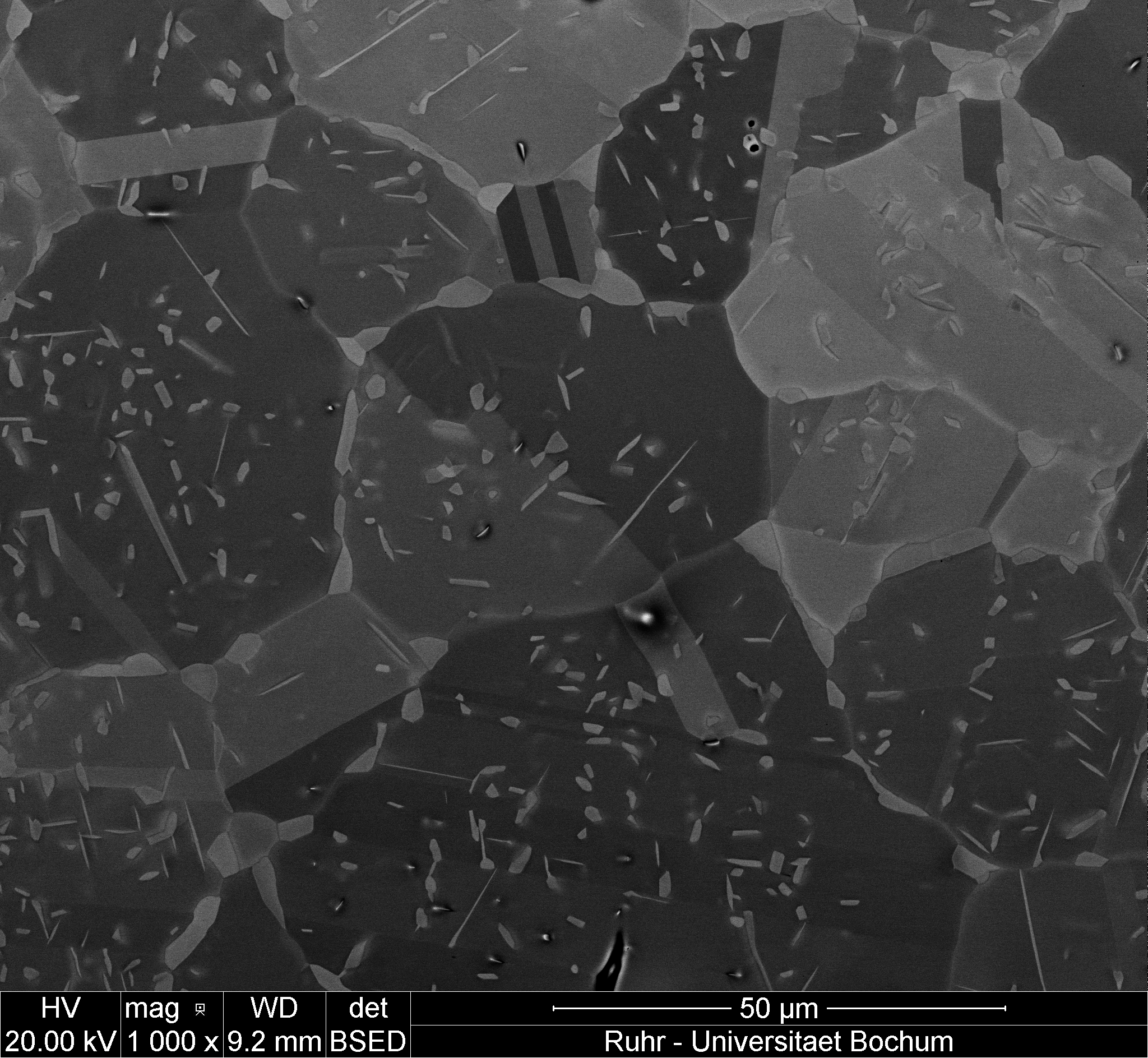

Supplement: Supplementary file 1 [file mmc1.zip › Upload_Data_in_Brief/BSE_microstructures/0700C_1000h/0700C_1000h_area4.tif]

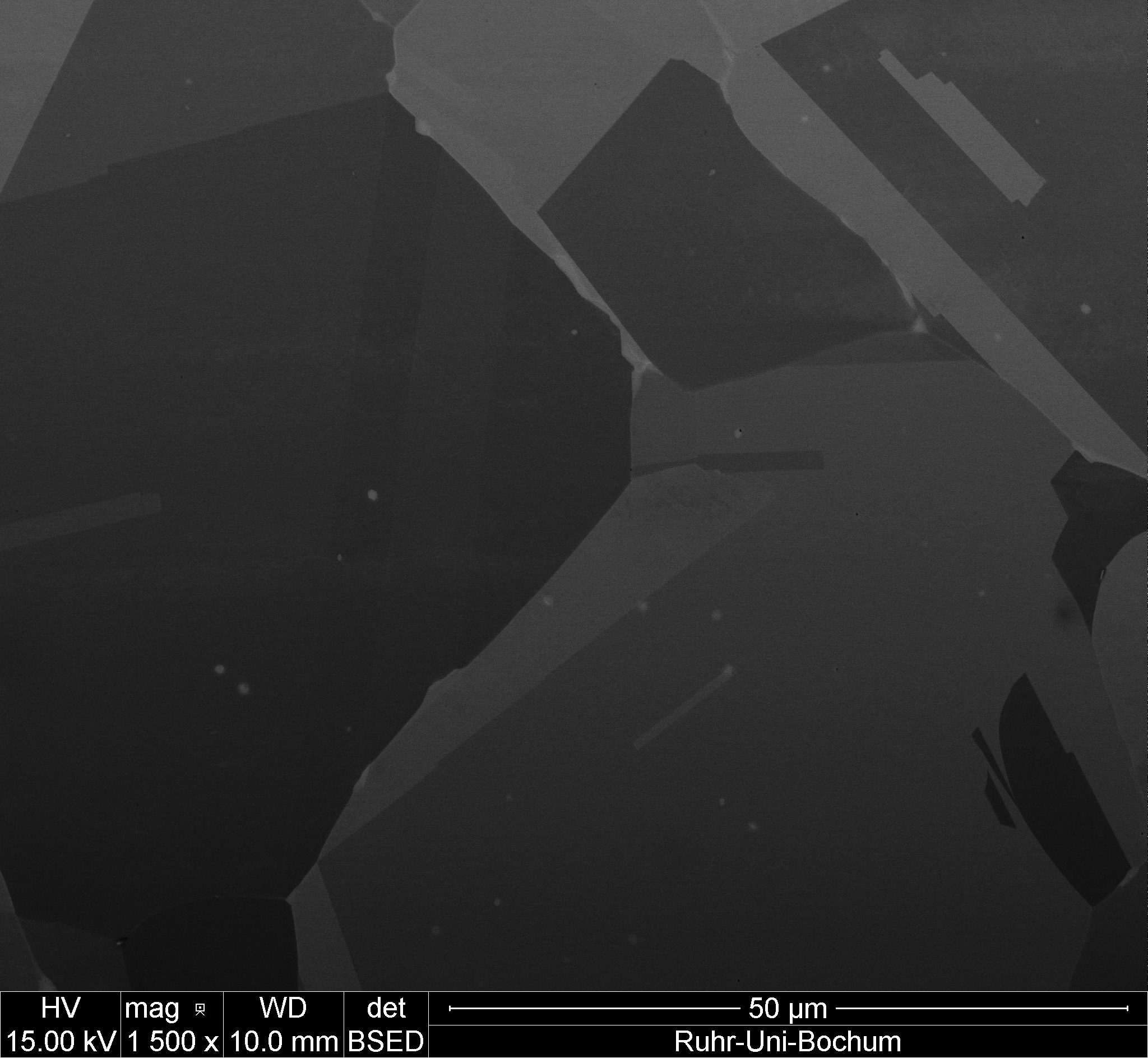

Supplement: Supplementary file 1 [file mmc1.zip › Upload_Data_in_Brief/BSE_microstructures/0800C_0001h/0800C_0001h_area1.tif]

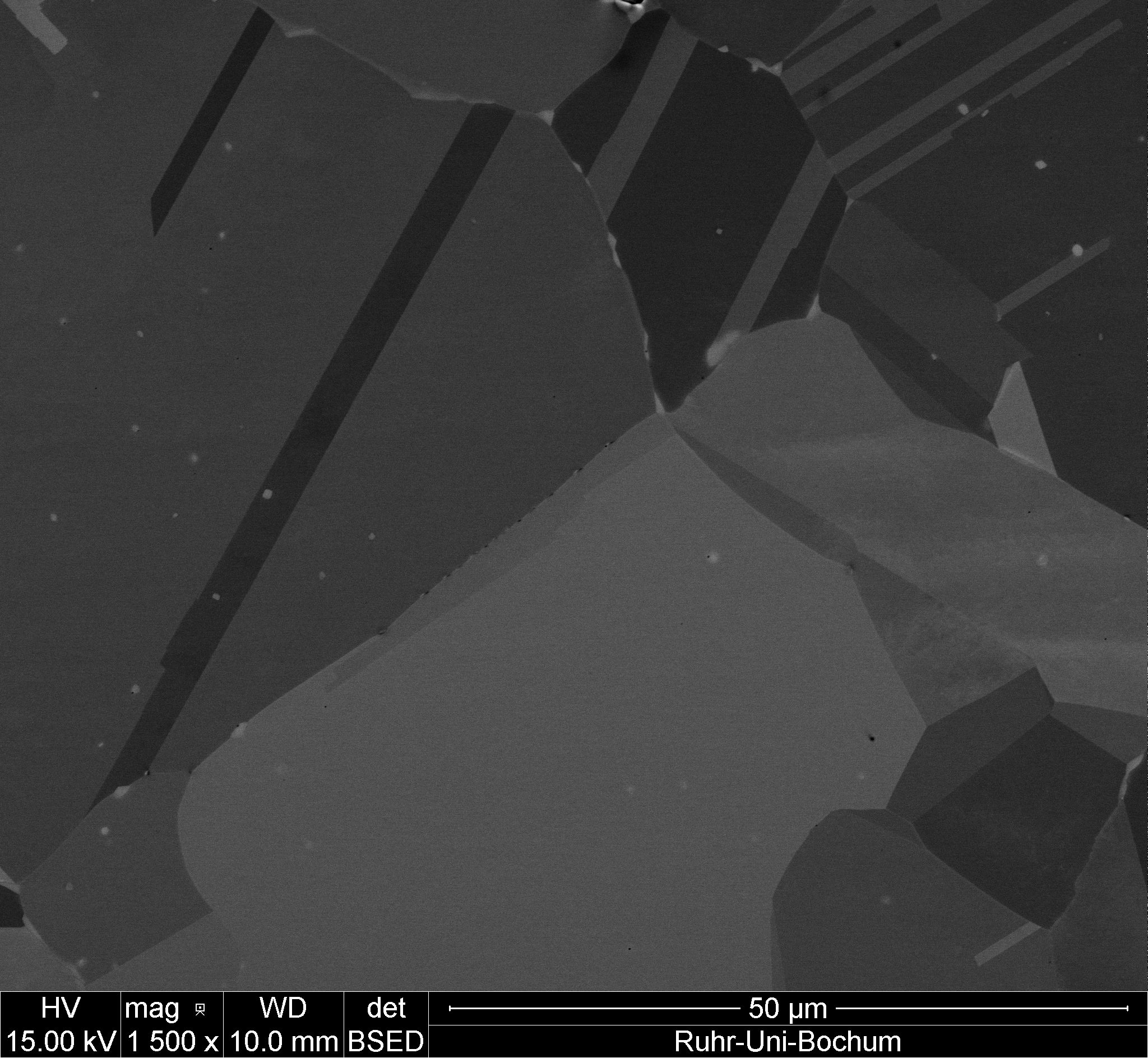

Supplement: Supplementary file 1 [file mmc1.zip › Upload_Data_in_Brief/BSE_microstructures/0800C_0001h/0800C_0001h_area2.tif]

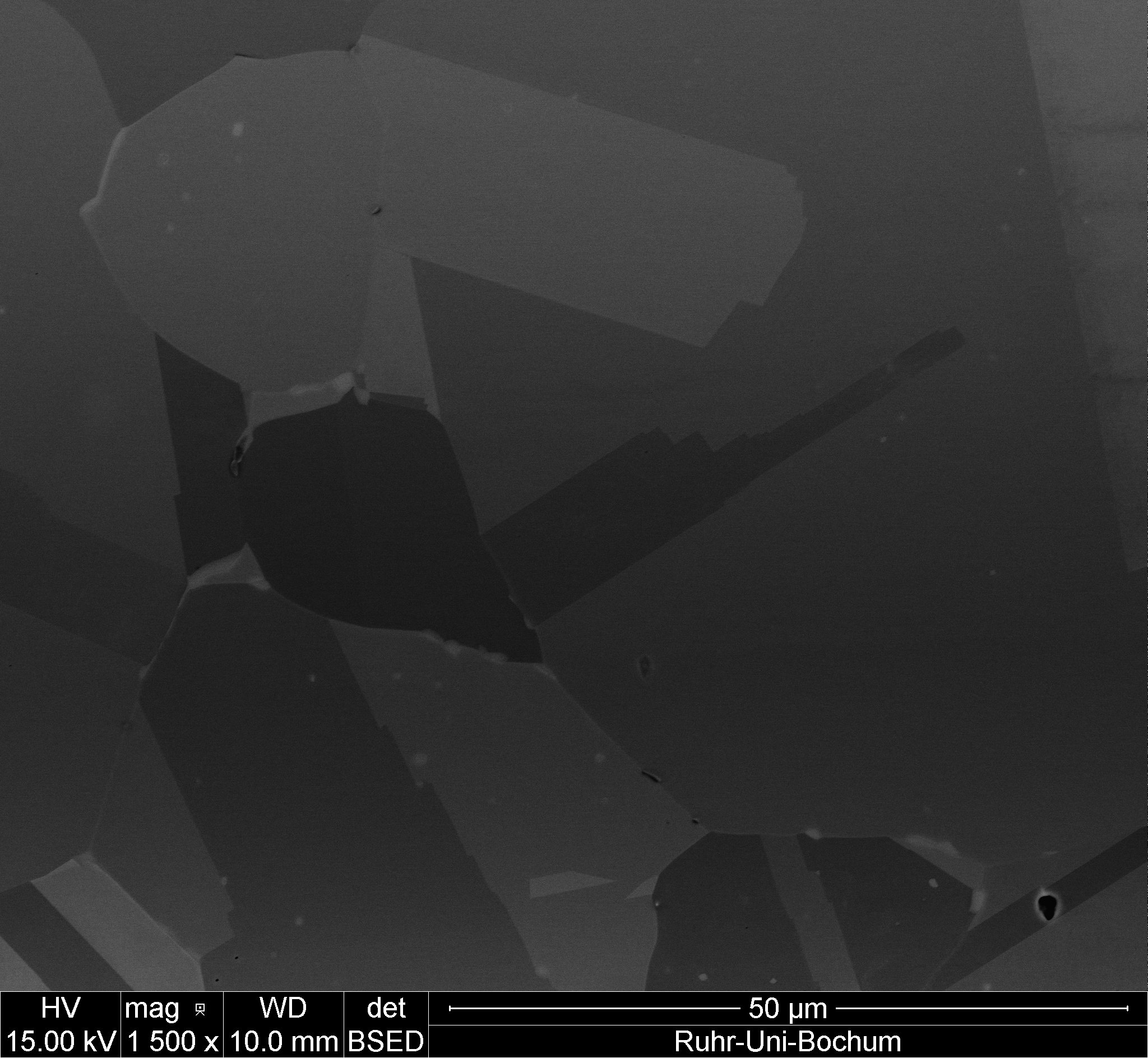

Supplement: Supplementary file 1 [file mmc1.zip › Upload_Data_in_Brief/BSE_microstructures/0800C_0001h/0800C_0001h_area3.tif]

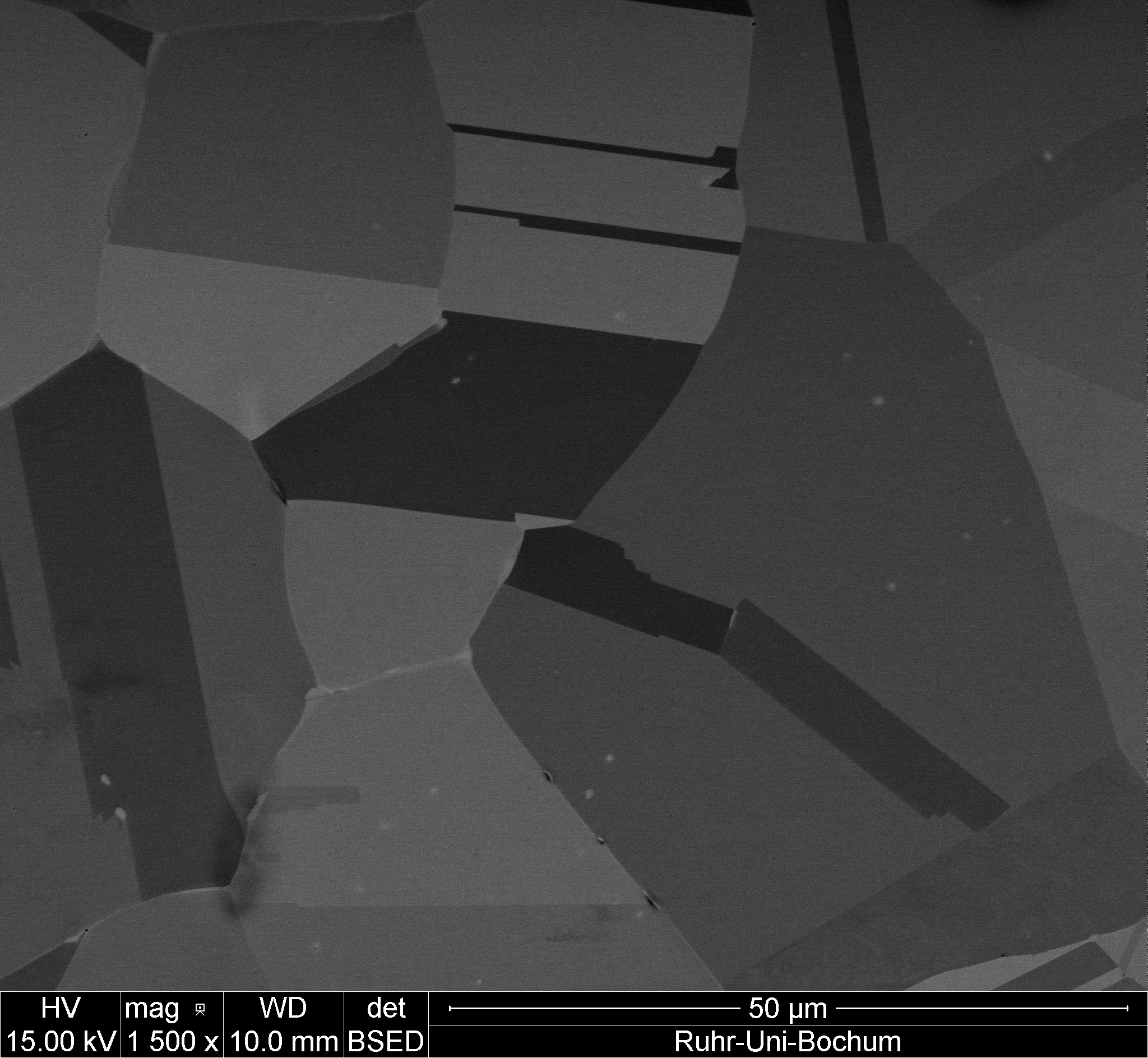

Supplement: Supplementary file 1 [file mmc1.zip › Upload_Data_in_Brief/BSE_microstructures/0800C_0001h/0800C_0001h_area4.tif]

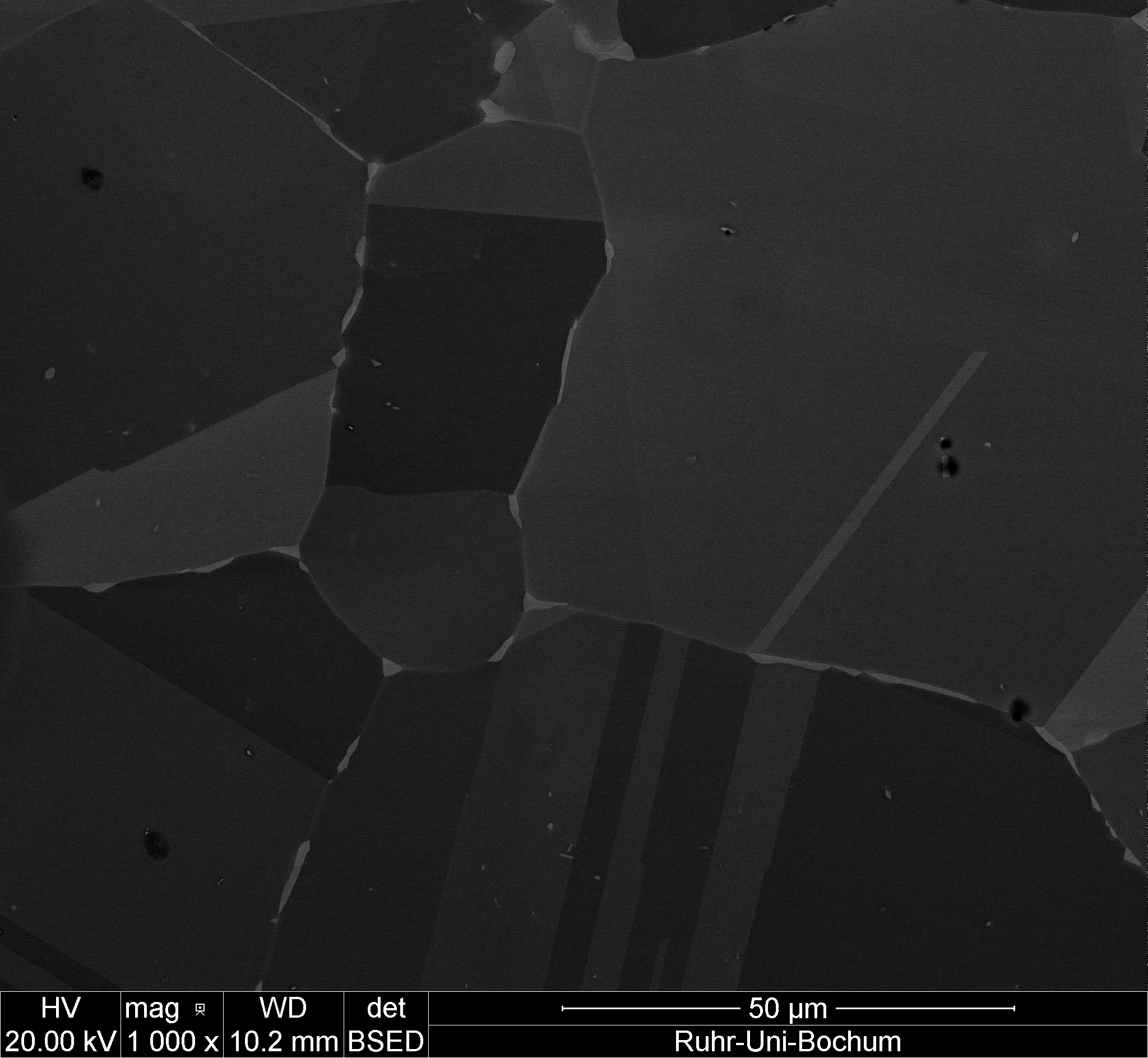

Supplement: Supplementary file 1 [file mmc1.zip › Upload_Data_in_Brief/BSE_microstructures/0800C_0010h/0800C_0010h_area1.tif]

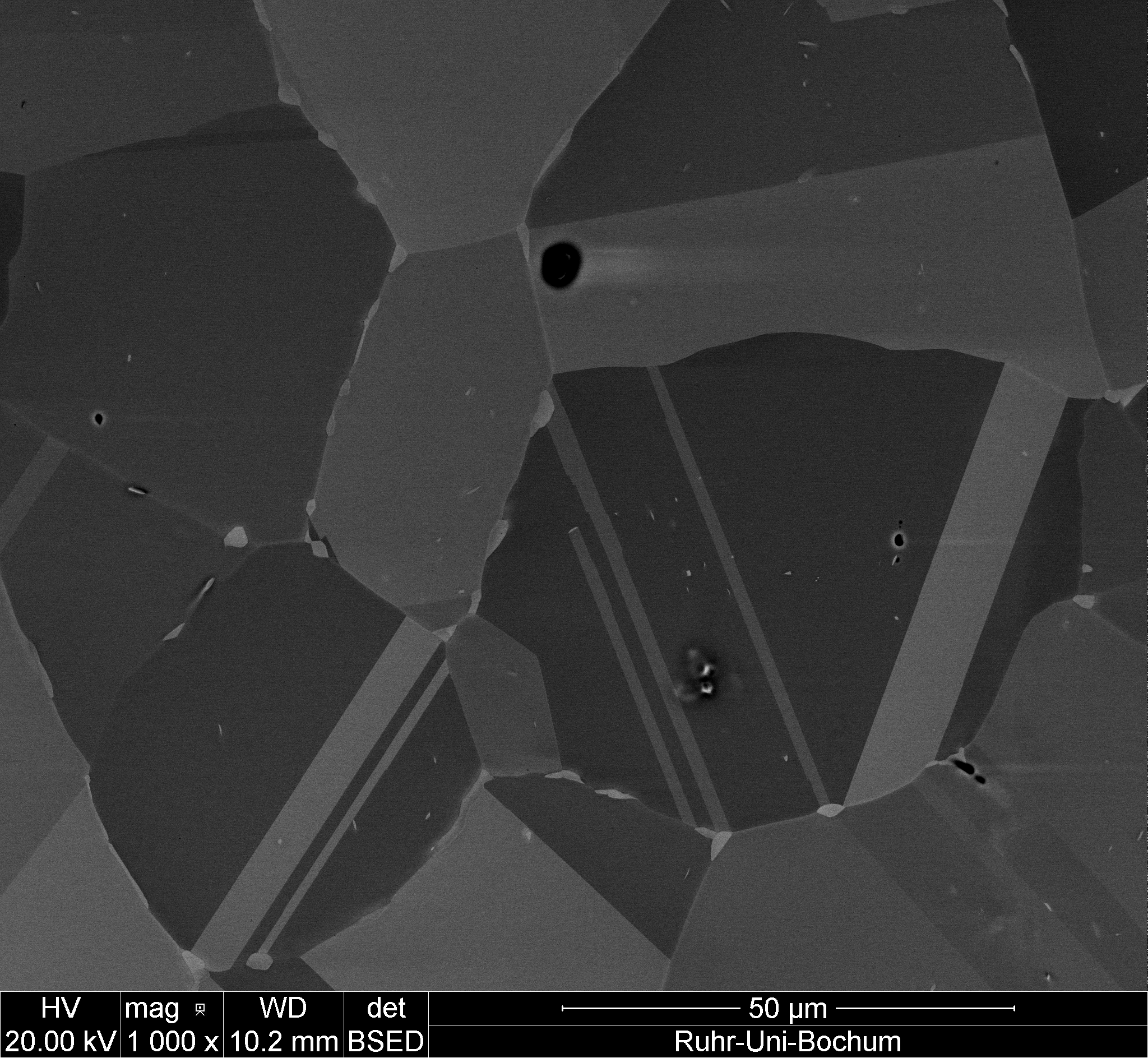

Supplement: Supplementary file 1 [file mmc1.zip › Upload_Data_in_Brief/BSE_microstructures/0800C_0010h/0800C_0010h_area2.tif]

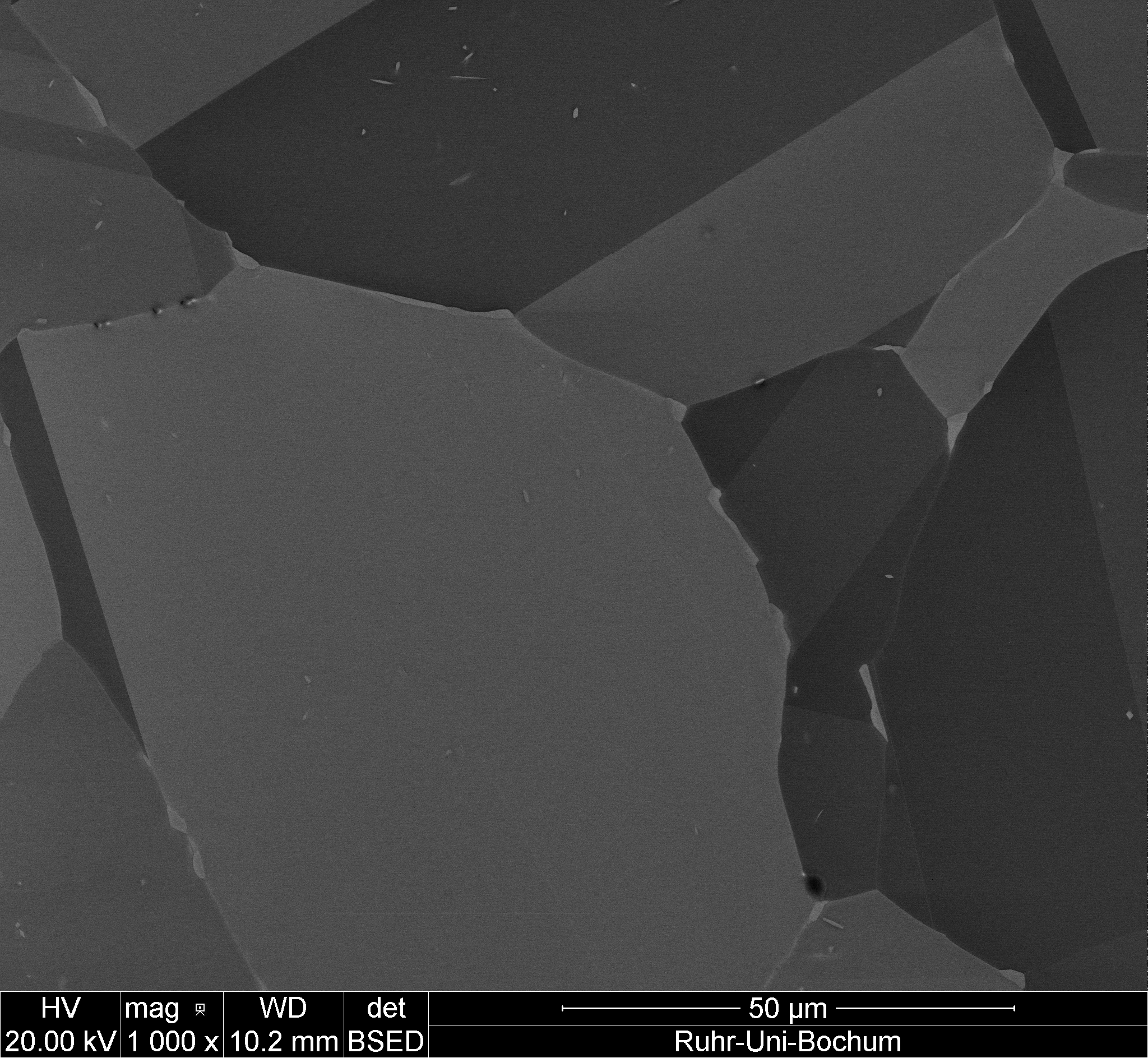

Supplement: Supplementary file 1 [file mmc1.zip › Upload_Data_in_Brief/BSE_microstructures/0800C_0010h/0800C_0010h_area3.tif]

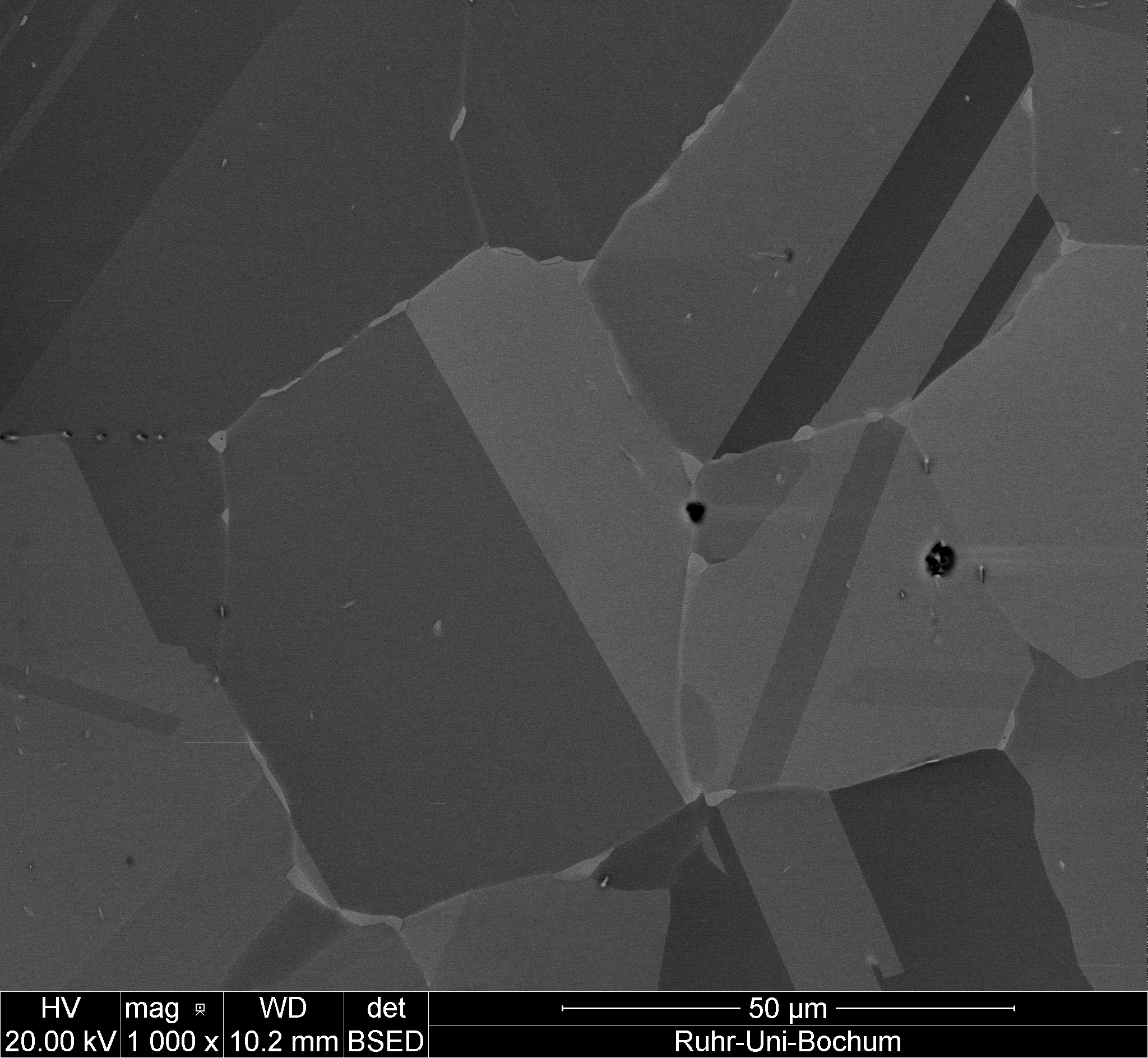

Supplement: Supplementary file 1 [file mmc1.zip › Upload_Data_in_Brief/BSE_microstructures/0800C_0010h/0800C_0010h_area4.tif]

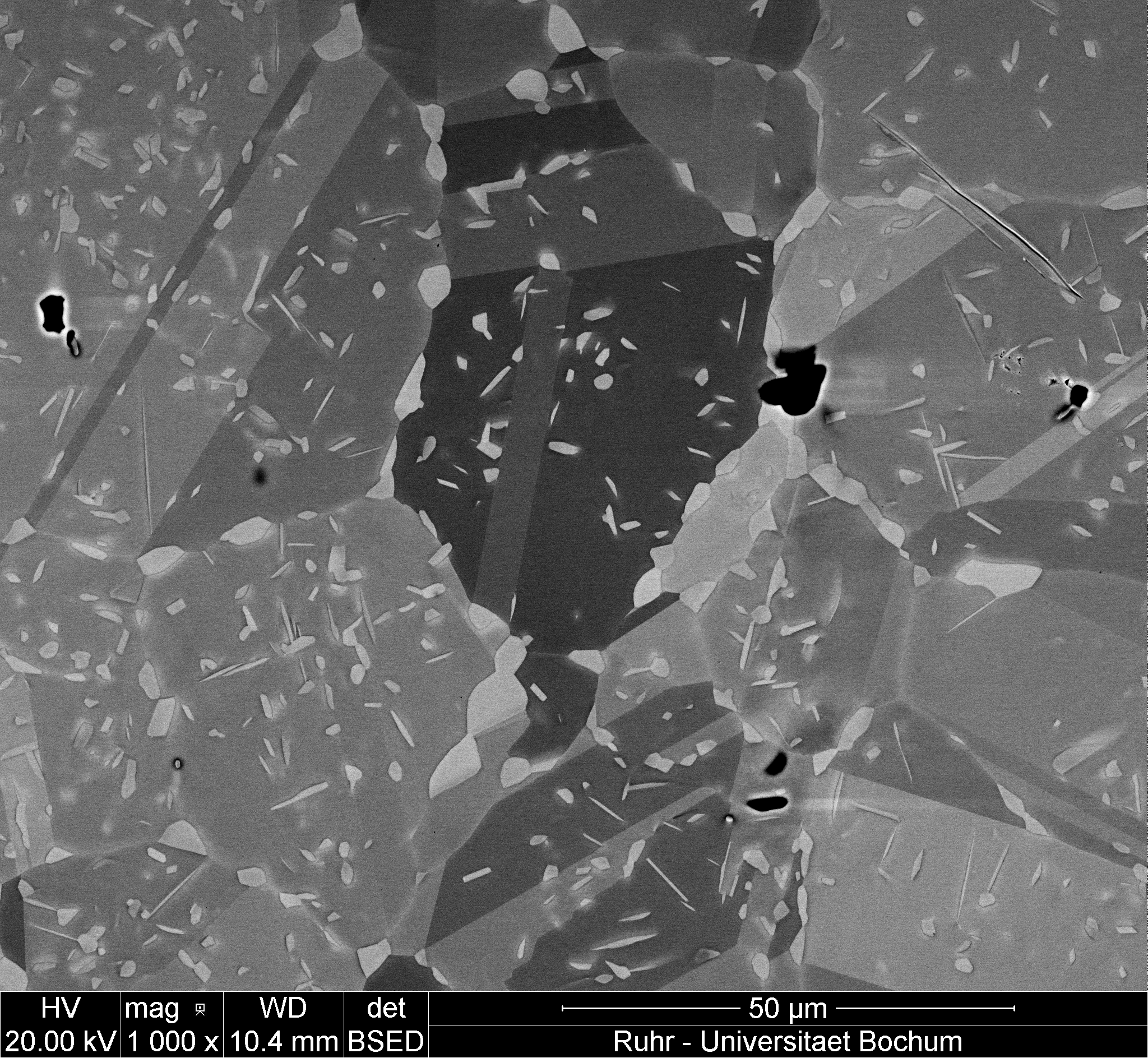

Supplement: Supplementary file 1 [file mmc1.zip › Upload_Data_in_Brief/BSE_microstructures/0800C_0100h/0800C_0100h_area1.tif]

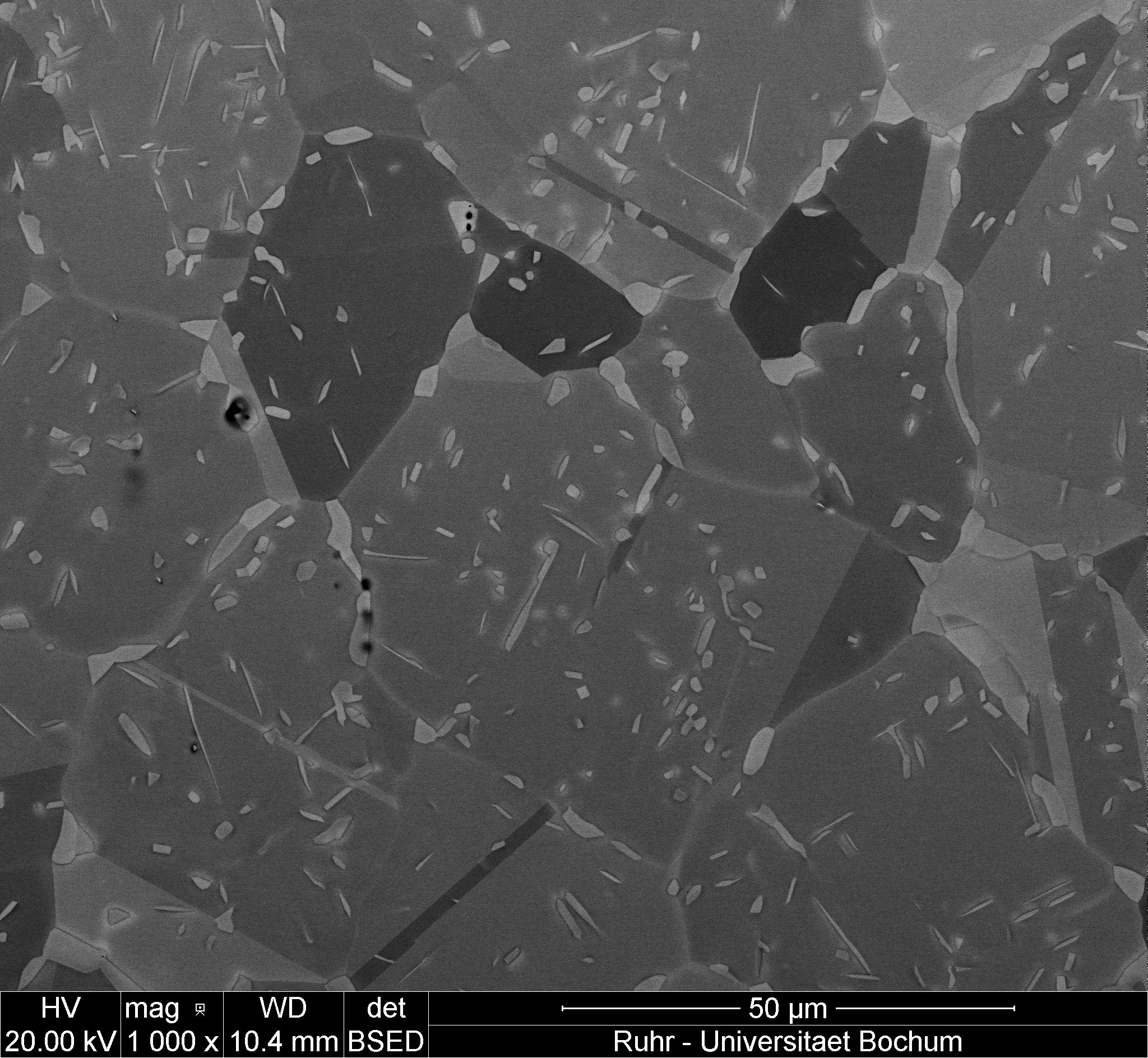

Supplement: Supplementary file 1 [file mmc1.zip › Upload_Data_in_Brief/BSE_microstructures/0800C_0100h/0800C_0100h_area2.tif]

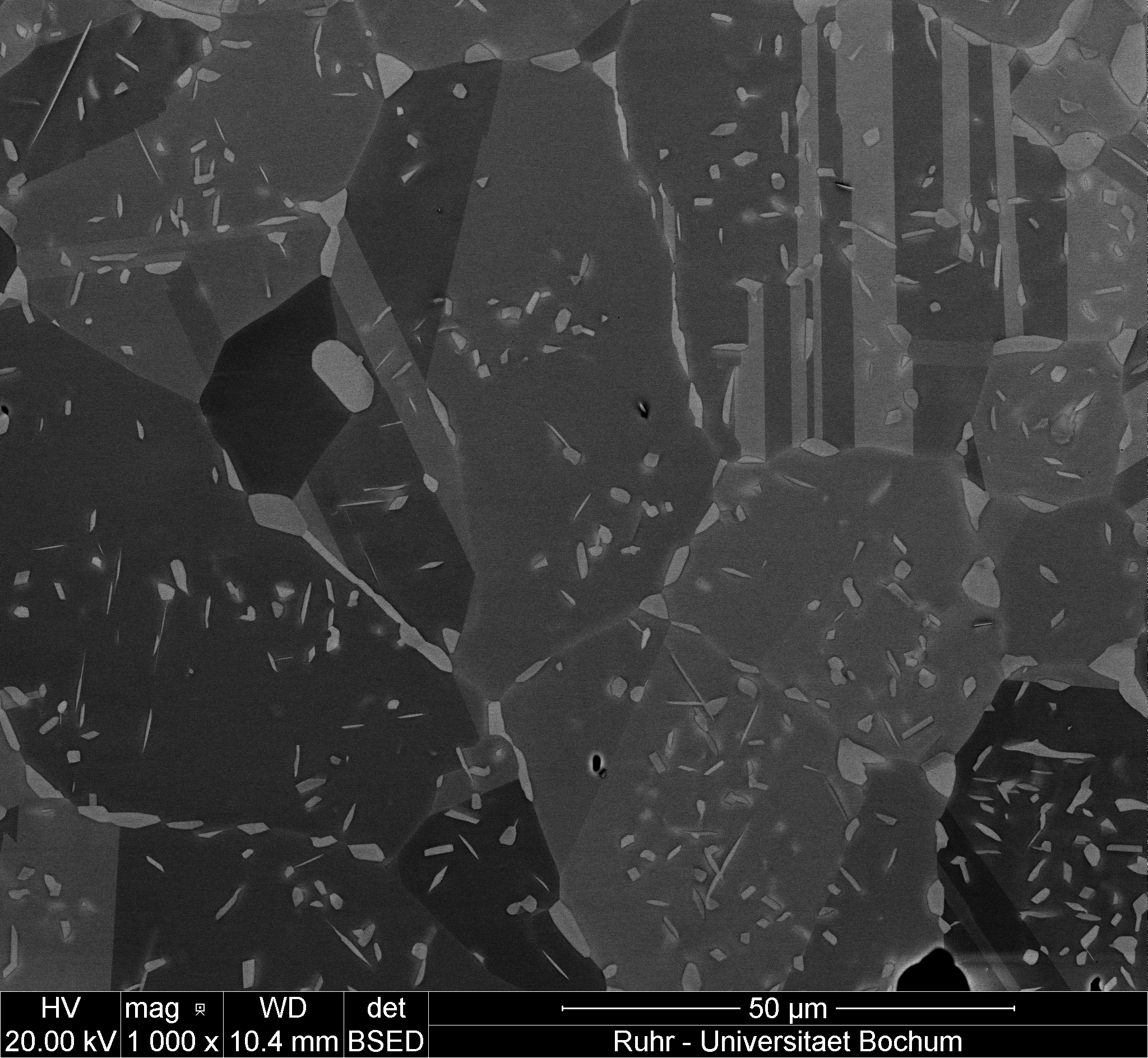

Supplement: Supplementary file 1 [file mmc1.zip › Upload_Data_in_Brief/BSE_microstructures/0800C_0100h/0800C_0100h_area3.tif]

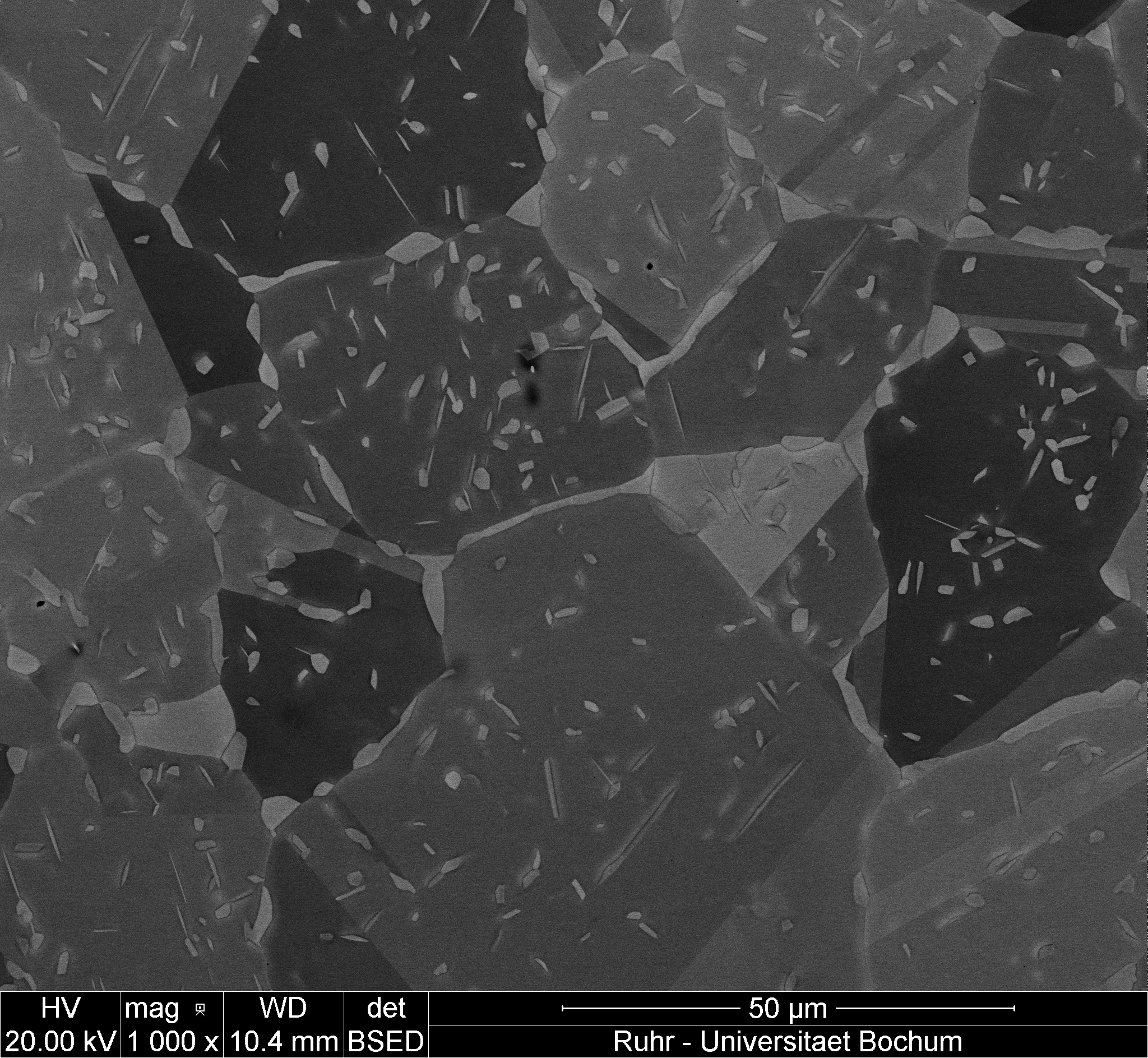

Supplement: Supplementary file 1 [file mmc1.zip › Upload_Data_in_Brief/BSE_microstructures/0800C_0100h/0800C_0100h_area4.tif]

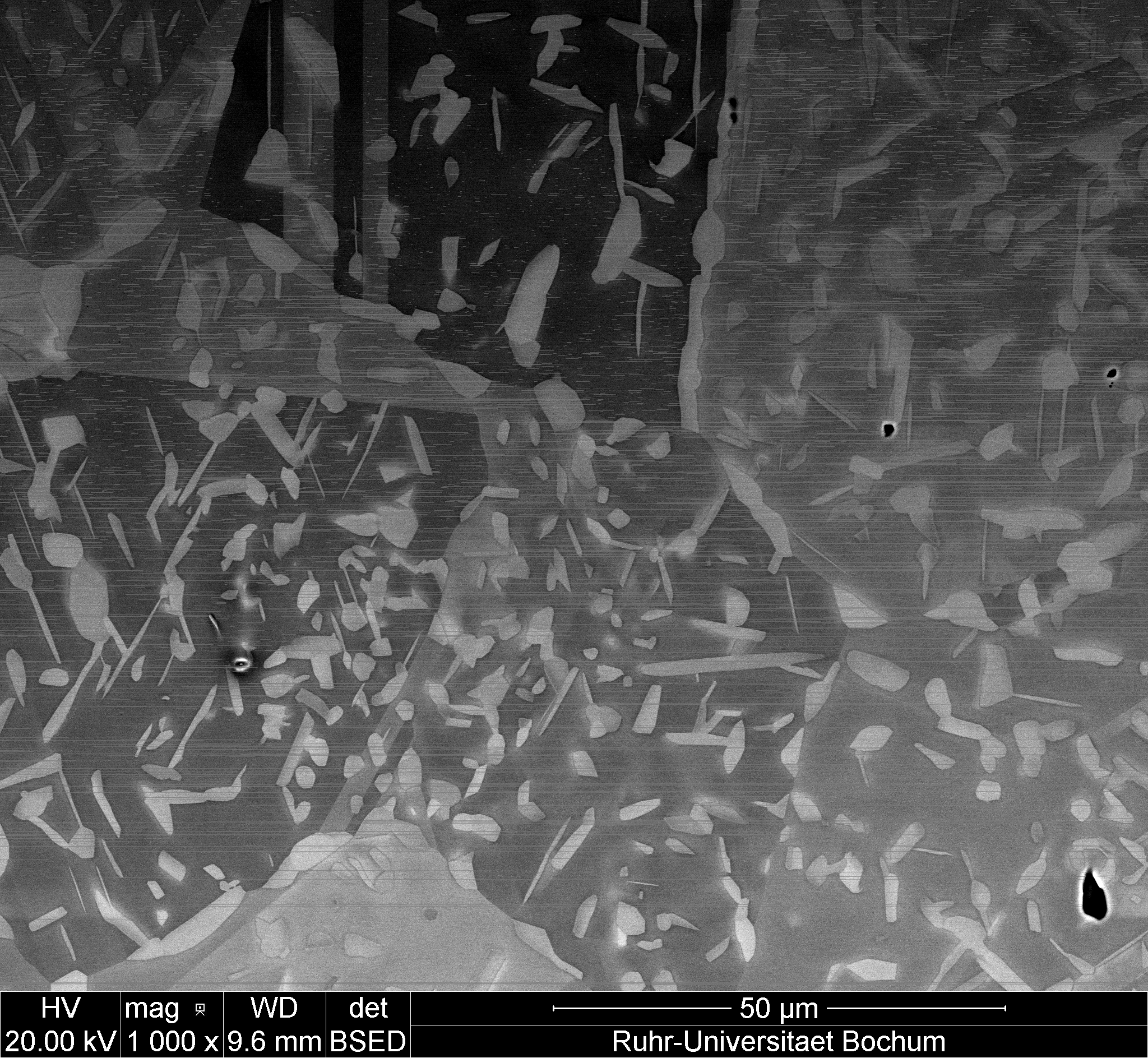

Supplement: Supplementary file 1 [file mmc1.zip › Upload_Data_in_Brief/BSE_microstructures/0800C_0500h/0800C_0500h_area1.tif]

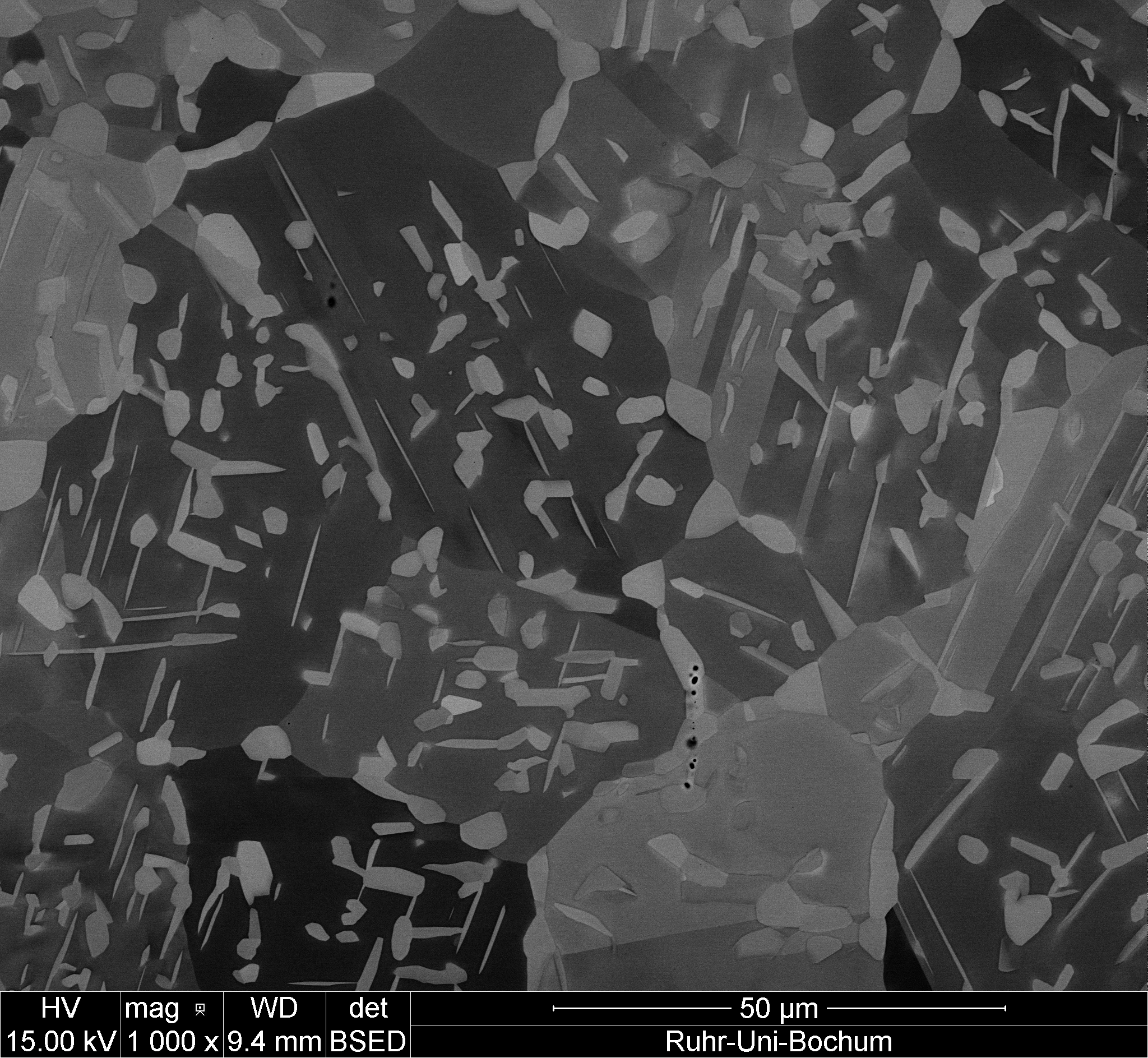

Supplement: Supplementary file 1 [file mmc1.zip › Upload_Data_in_Brief/BSE_microstructures/0800C_0500h/0800C_0500h_area2.tif]

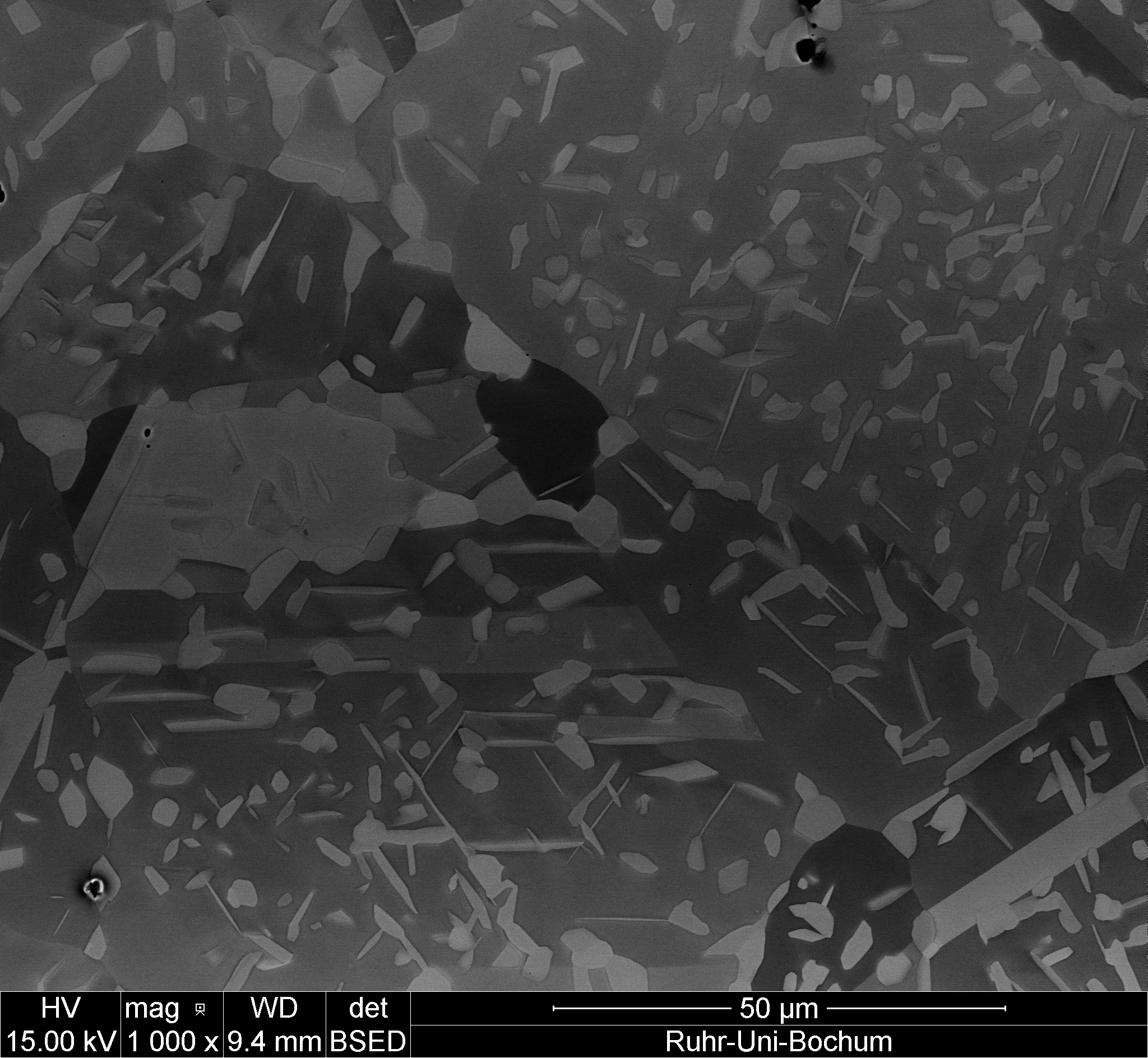

Supplement: Supplementary file 1 [file mmc1.zip › Upload_Data_in_Brief/BSE_microstructures/0800C_0500h/0800C_0500h_area3.tif]

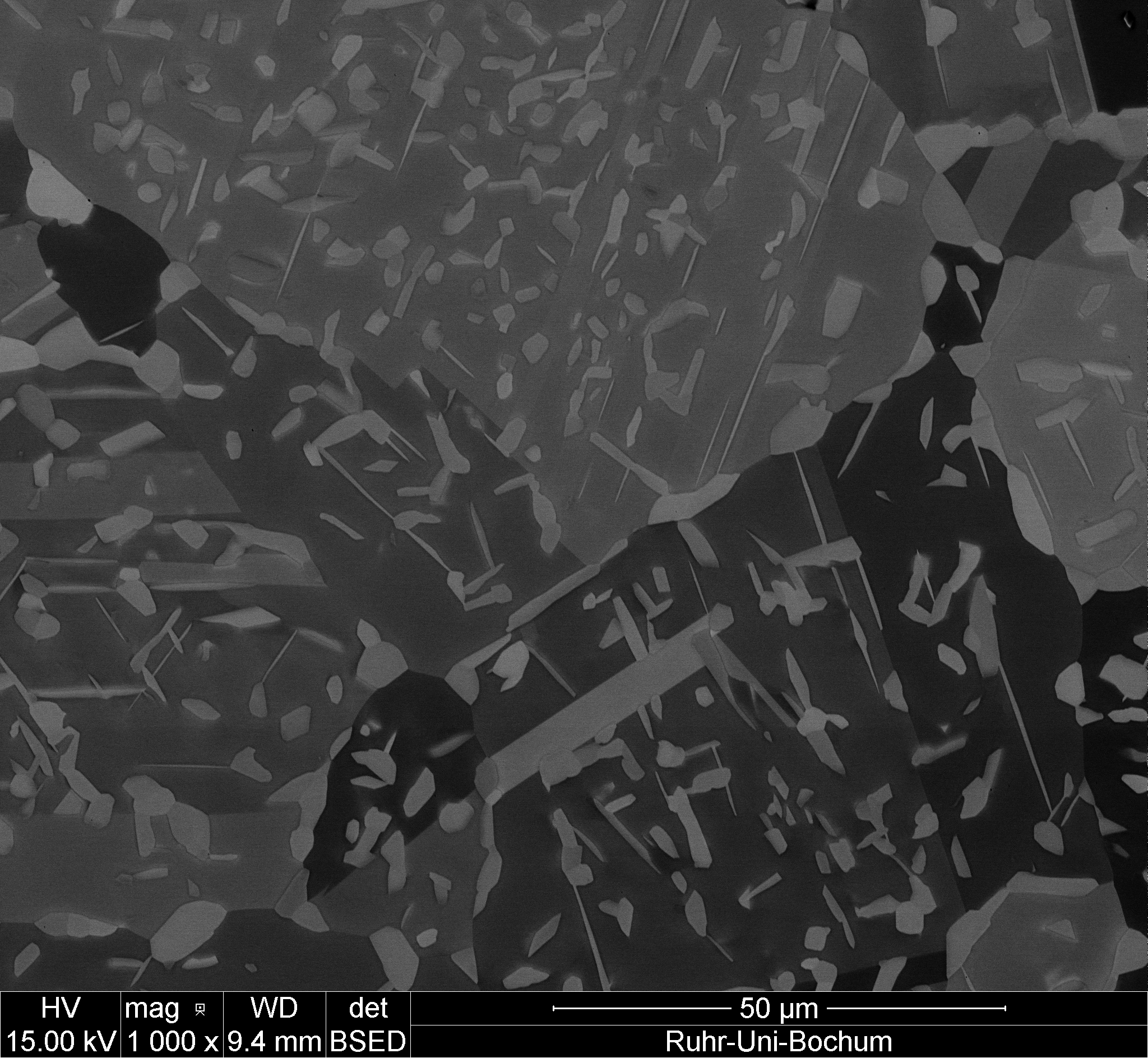

Supplement: Supplementary file 1 [file mmc1.zip › Upload_Data_in_Brief/BSE_microstructures/0800C_0500h/0800C_0500h_area4.tif]

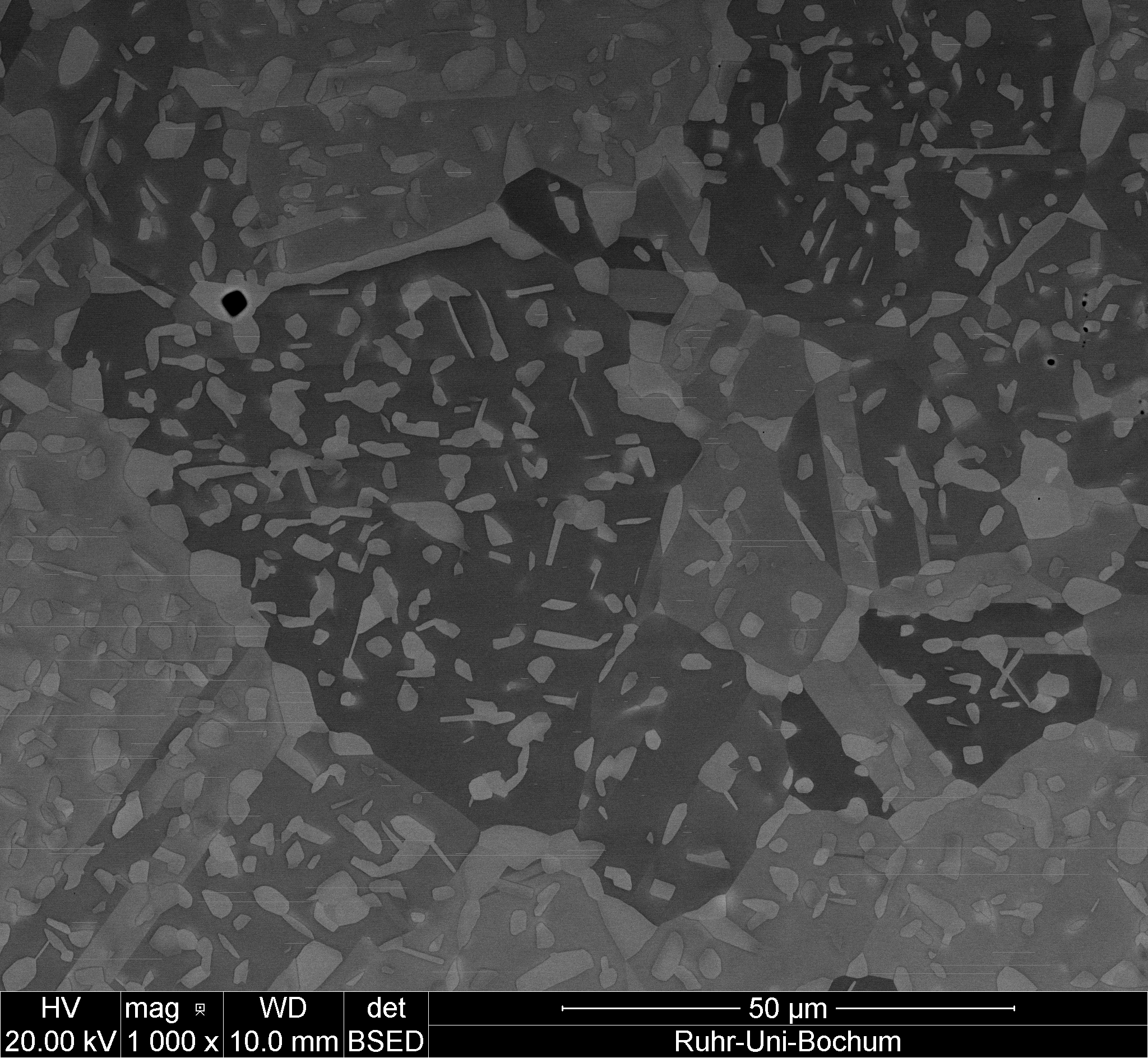

Supplement: Supplementary file 1 [file mmc1.zip › Upload_Data_in_Brief/BSE_microstructures/0800C_1000h/0800C_1000h_area1.tif]

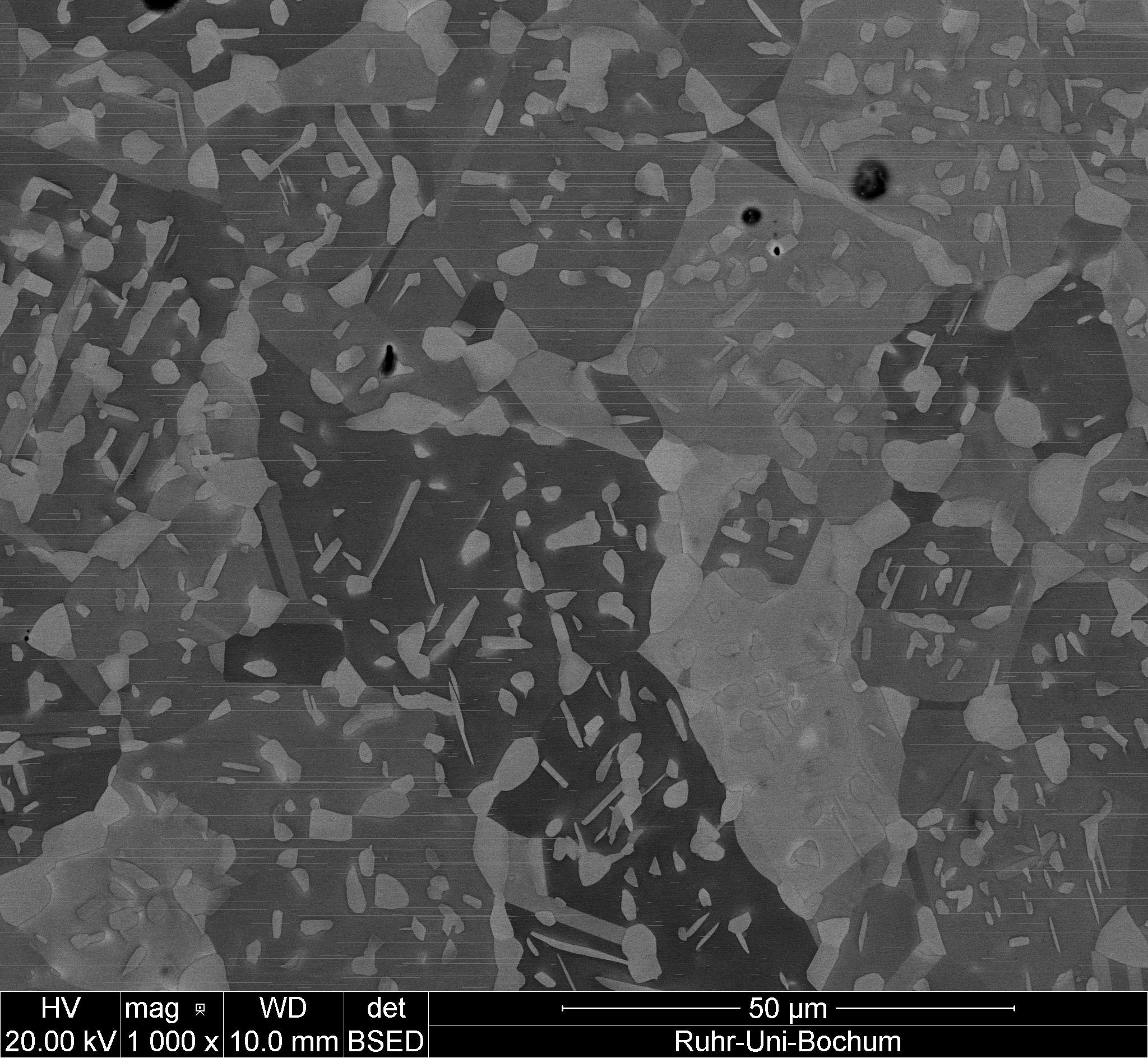

Supplement: Supplementary file 1 [file mmc1.zip › Upload_Data_in_Brief/BSE_microstructures/0800C_1000h/0800C_1000h_area2.tif]

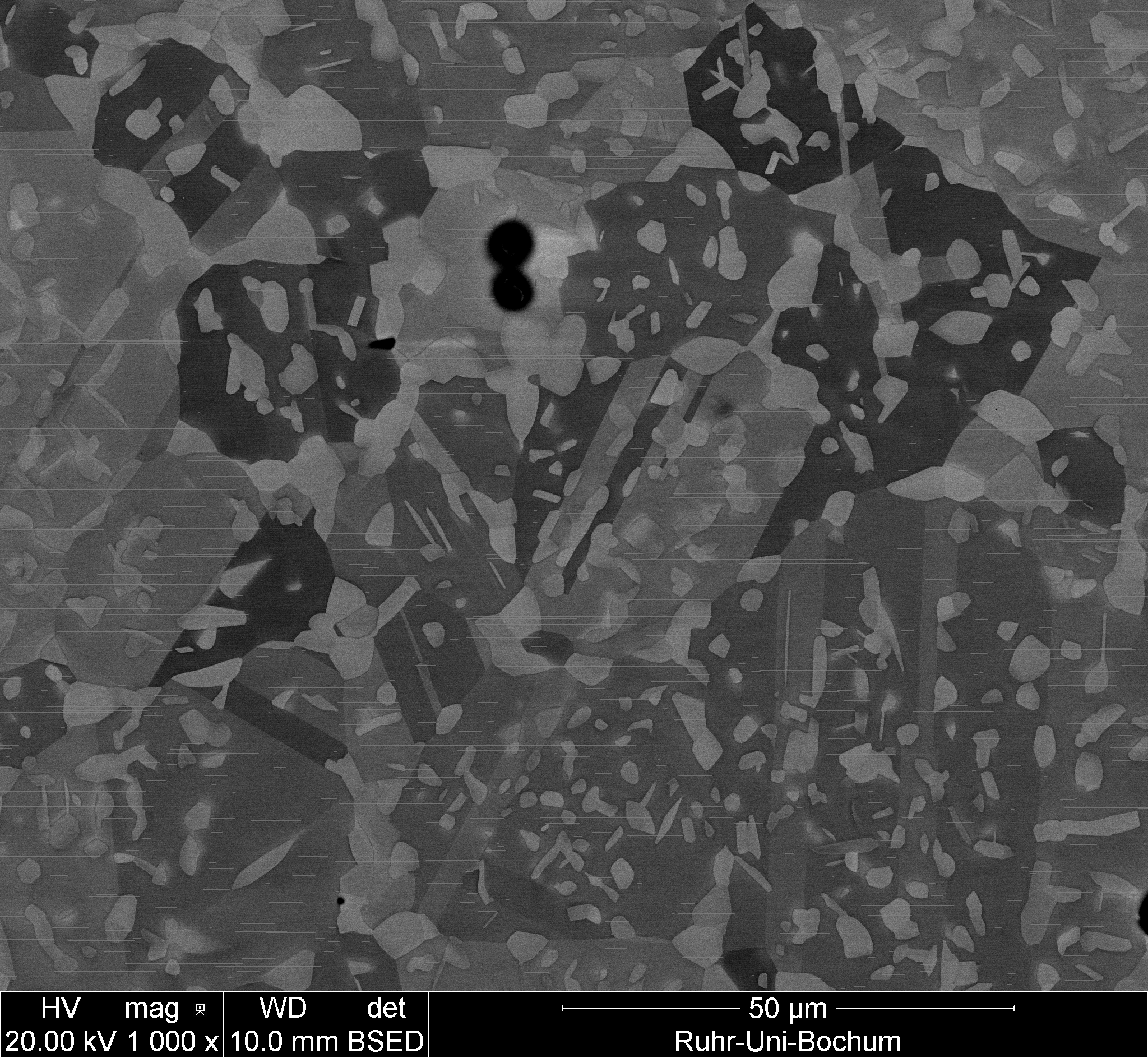

Supplement: Supplementary file 1 [file mmc1.zip › Upload_Data_in_Brief/BSE_microstructures/0800C_1000h/0800C_1000h_area3.tif]

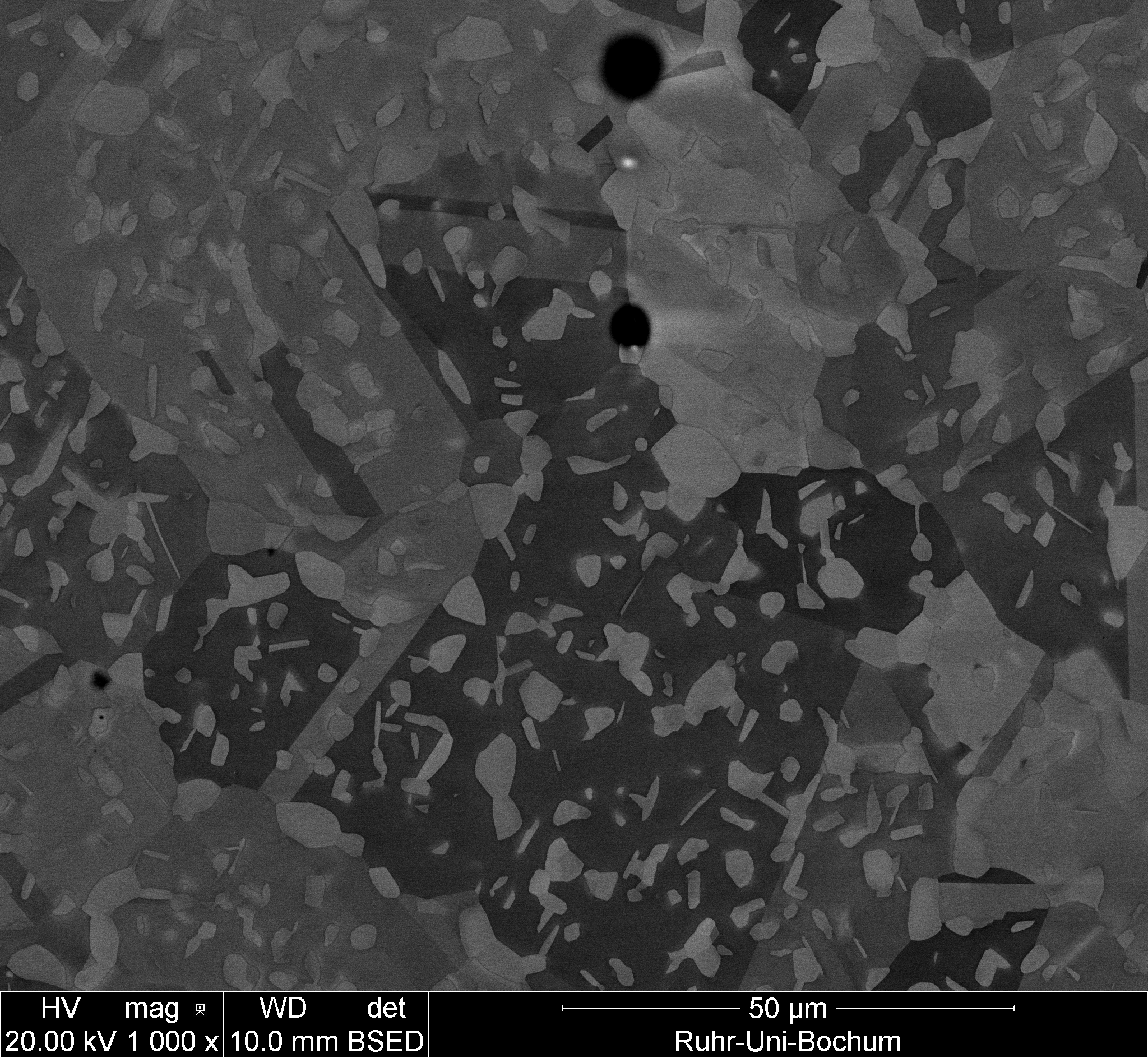

Supplement: Supplementary file 1 [file mmc1.zip › Upload_Data_in_Brief/BSE_microstructures/0800C_1000h/0800C_1000h_area4.tif]

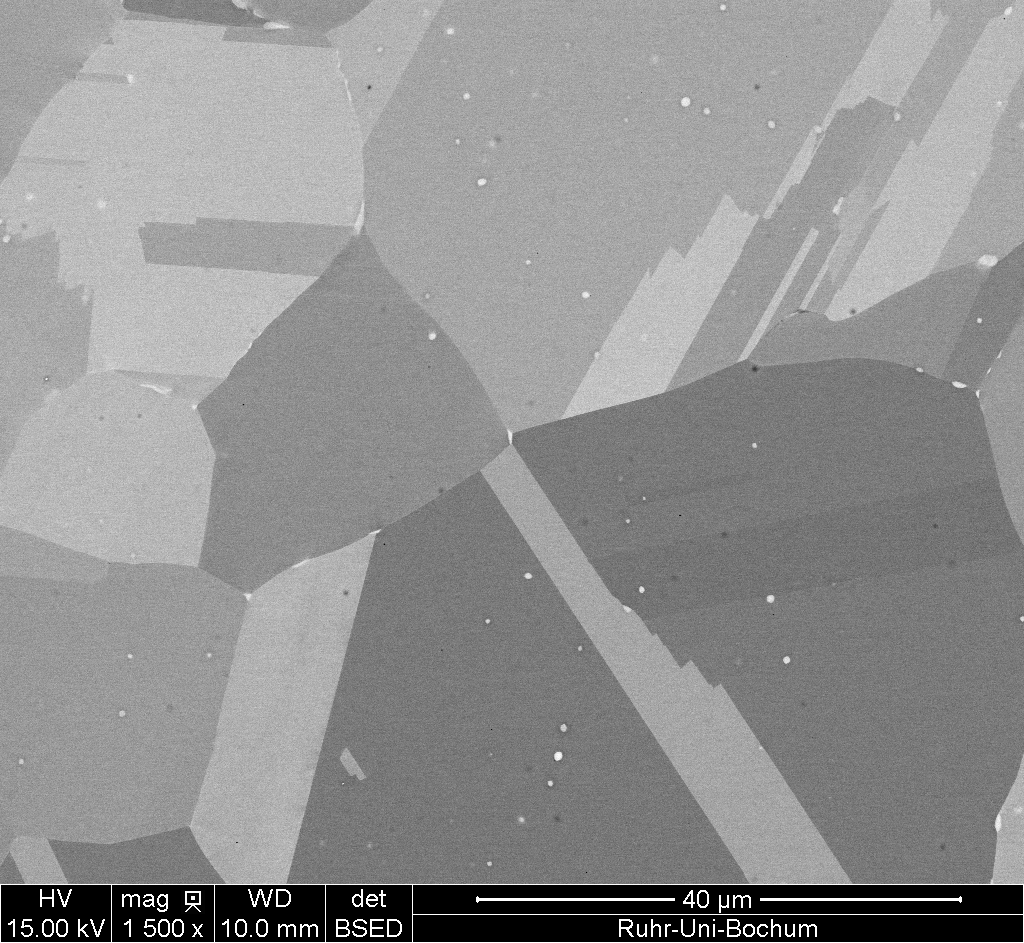

Supplement: Supplementary file 1 [file mmc1.zip › Upload_Data_in_Brief/BSE_microstructures/0900C_0.05h/0900C_0.05h_area1.tif]

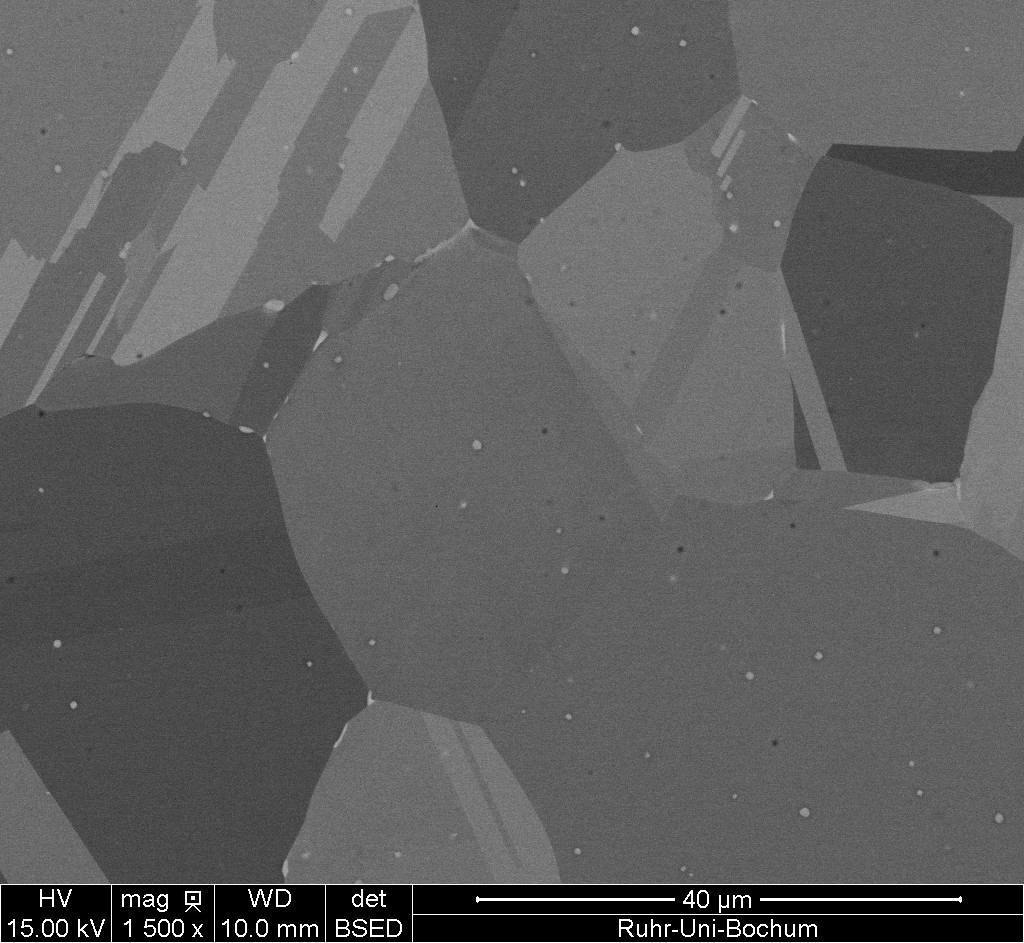

Supplement: Supplementary file 1 [file mmc1.zip › Upload_Data_in_Brief/BSE_microstructures/0900C_0.05h/0900C_0.05h_area2.tif]

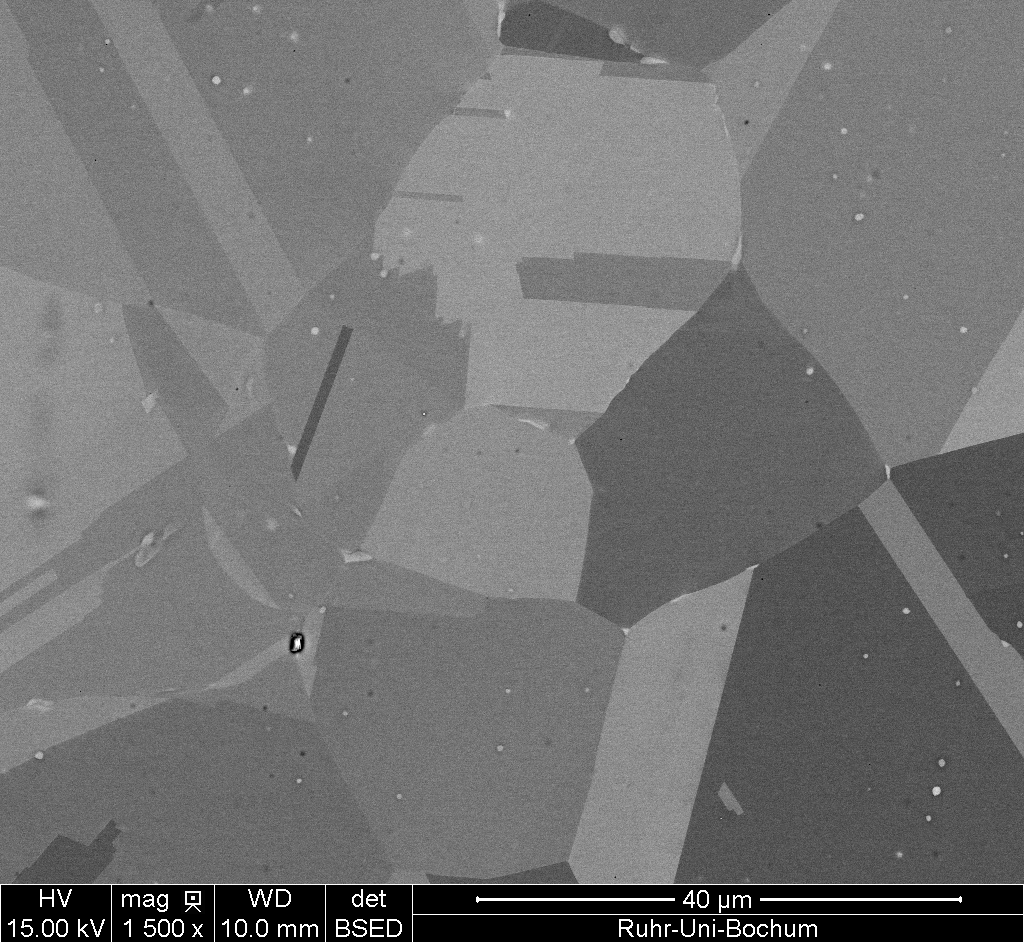

Supplement: Supplementary file 1 [file mmc1.zip › Upload_Data_in_Brief/BSE_microstructures/0900C_0.05h/0900C_0.05h_area3.tif]

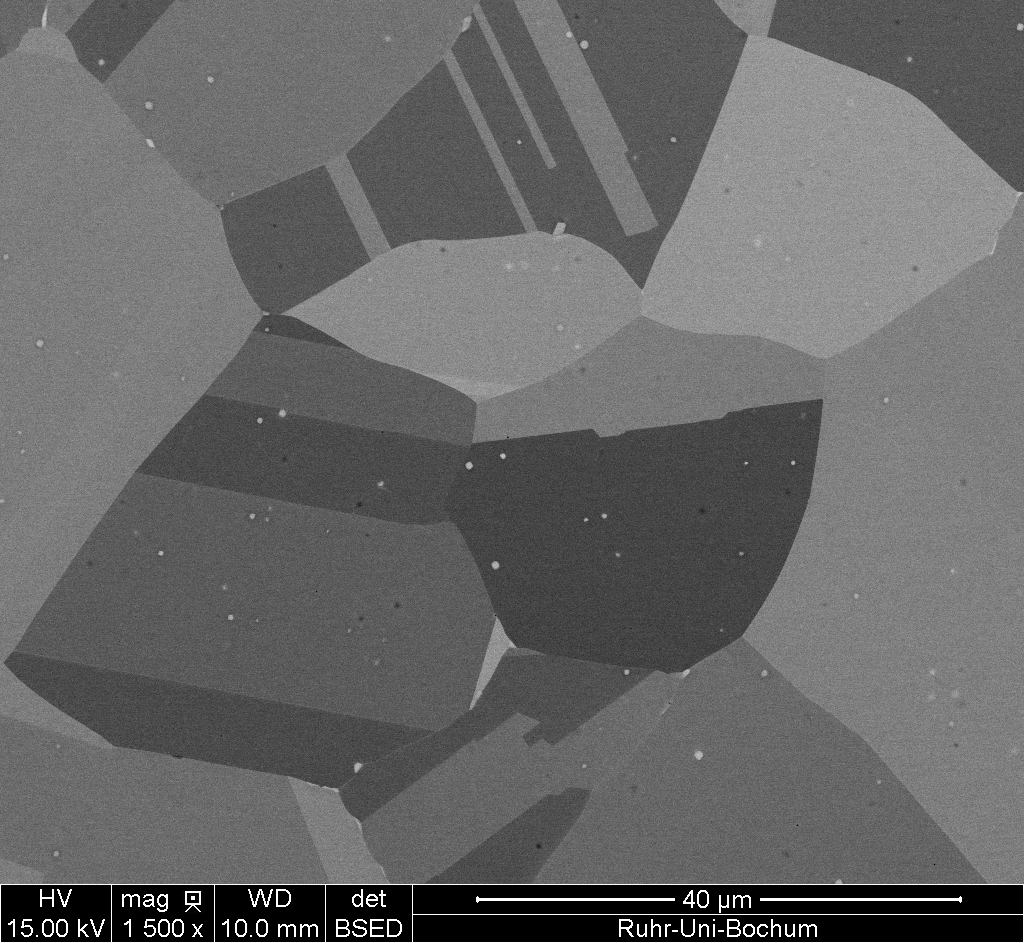

Supplement: Supplementary file 1 [file mmc1.zip › Upload_Data_in_Brief/BSE_microstructures/0900C_0.05h/0900C_0.05h_area4.tif]

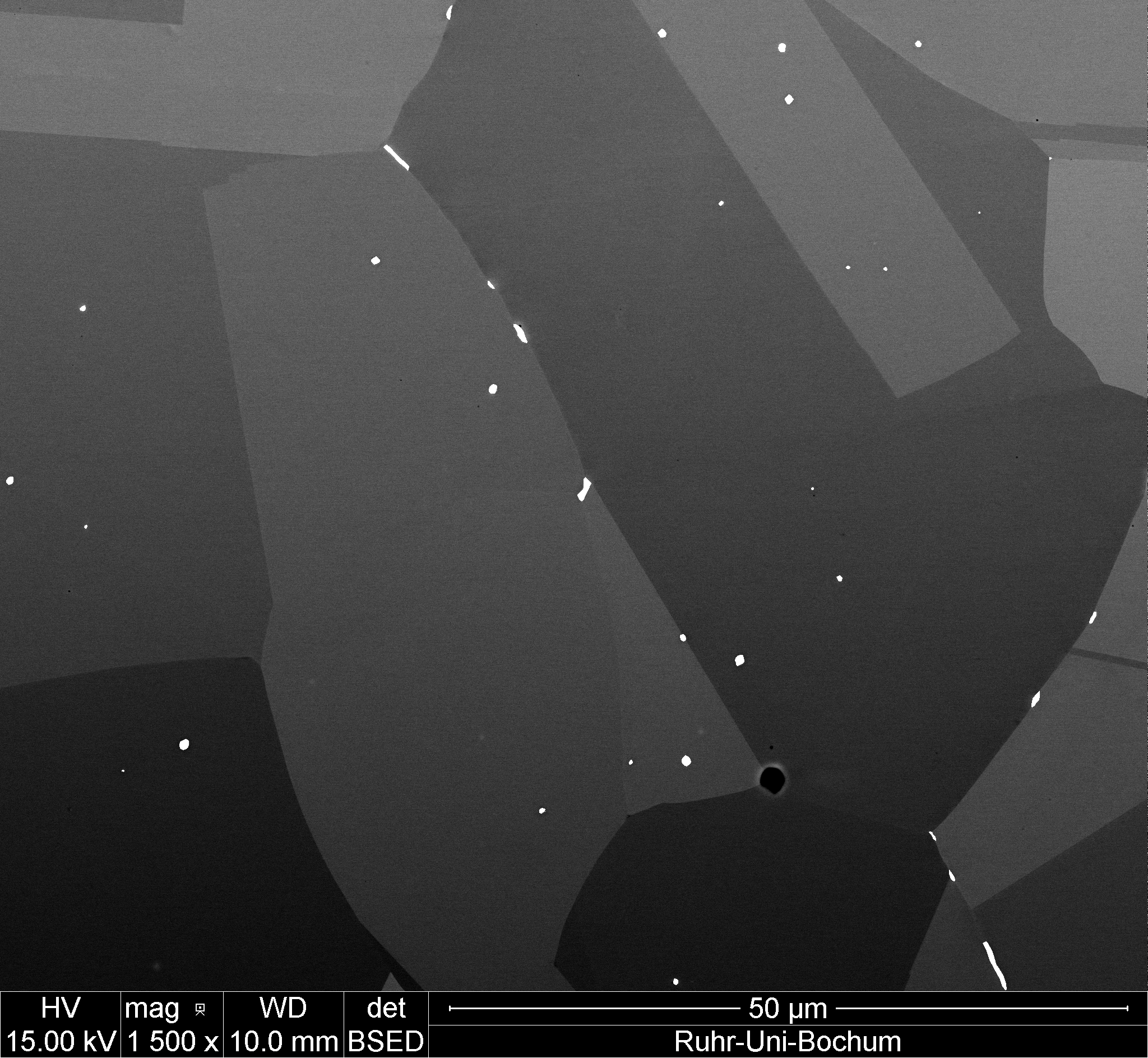

Supplement: Supplementary file 1 [file mmc1.zip › Upload_Data_in_Brief/BSE_microstructures/0900C_0.10h/0900C_0.10h_area1.tif]

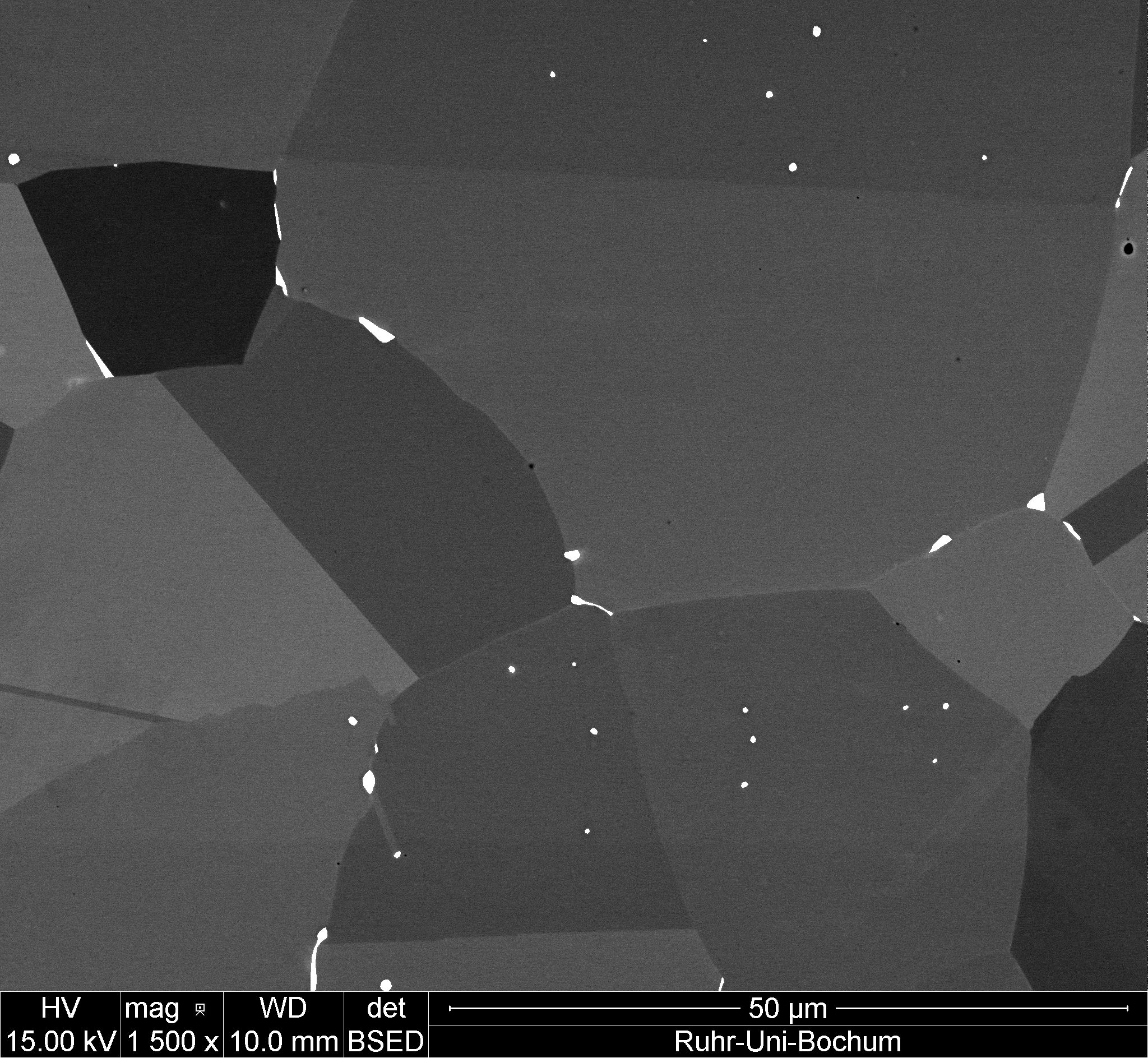

Supplement: Supplementary file 1 [file mmc1.zip › Upload_Data_in_Brief/BSE_microstructures/0900C_0.10h/0900C_0.10h_area2.tif]

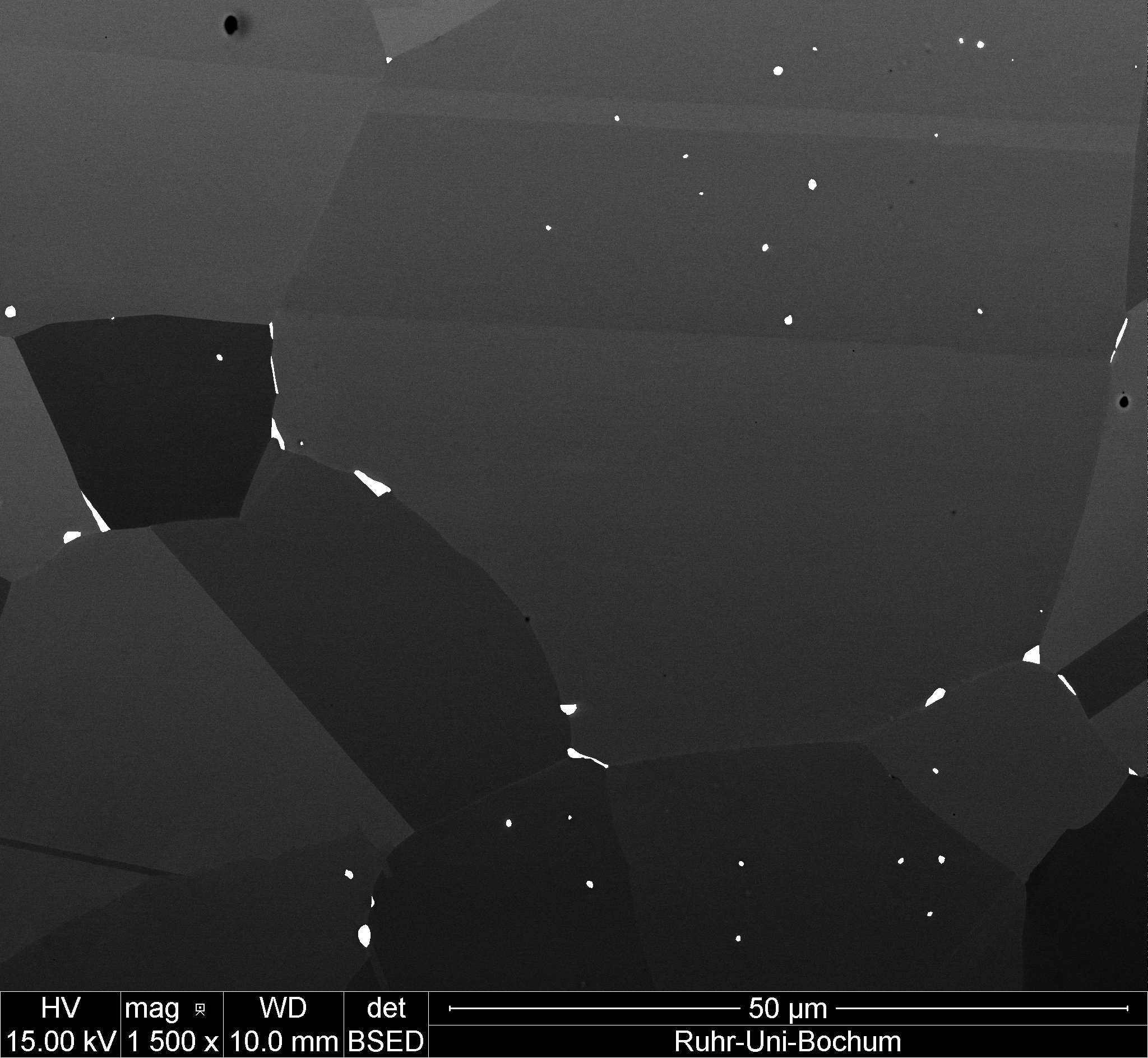

Supplement: Supplementary file 1 [file mmc1.zip › Upload_Data_in_Brief/BSE_microstructures/0900C_0.10h/0900C_0.10h_area3.tif]

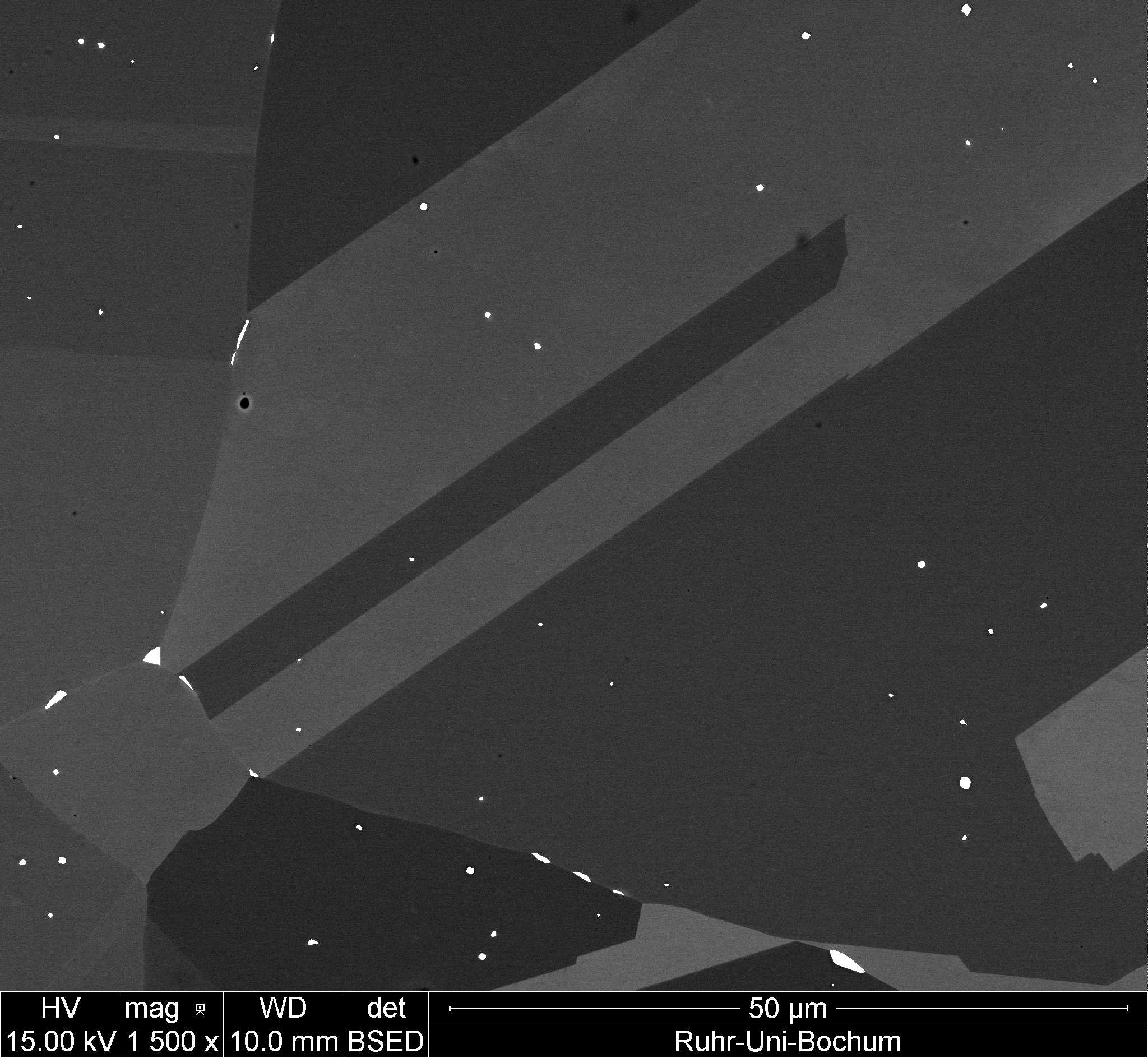

Supplement: Supplementary file 1 [file mmc1.zip › Upload_Data_in_Brief/BSE_microstructures/0900C_0.10h/0900C_0.10h_area4.tif]

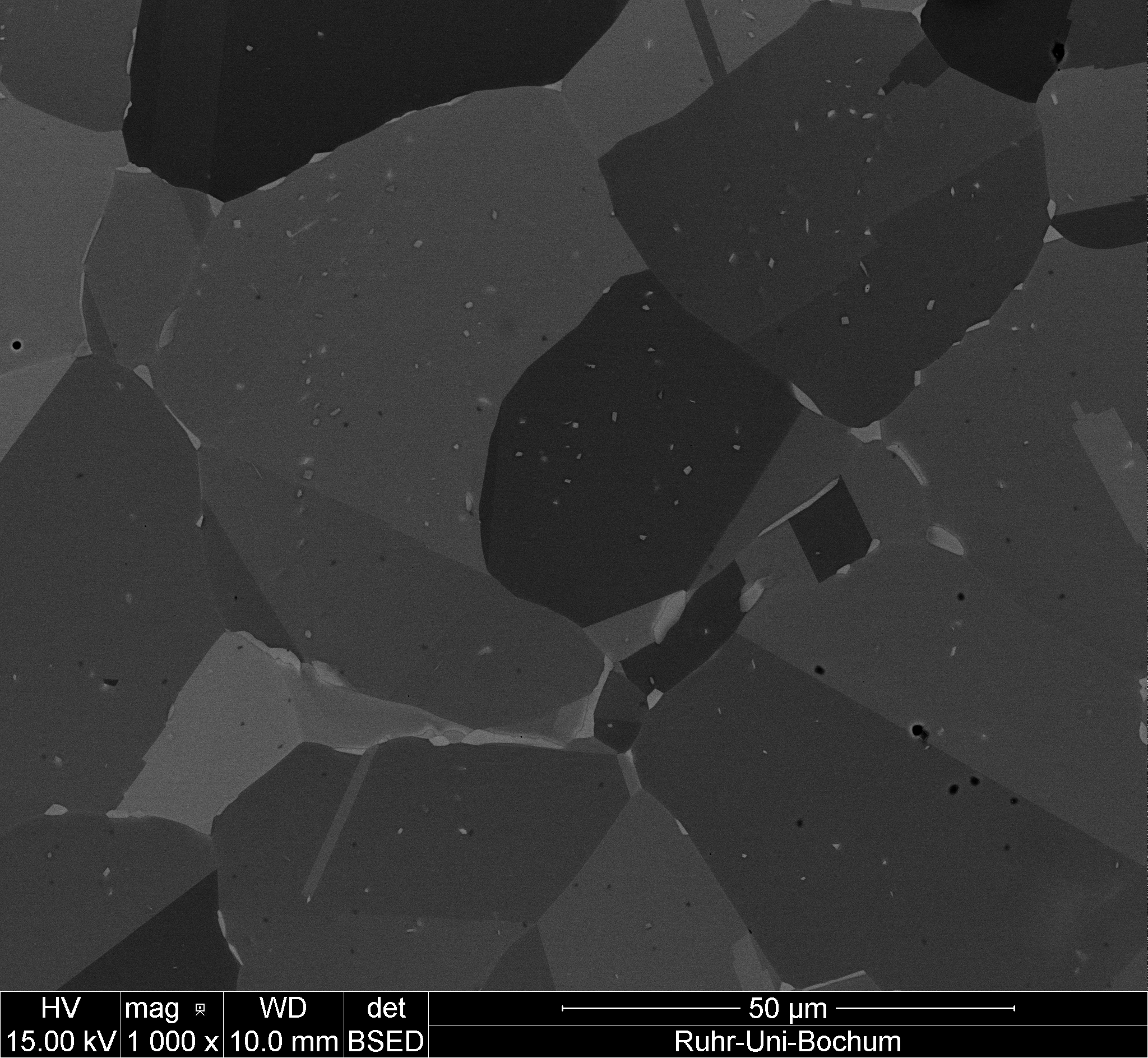

Supplement: Supplementary file 1 [file mmc1.zip › Upload_Data_in_Brief/BSE_microstructures/0900C_0001h/0900C_0001h_area1.tif]

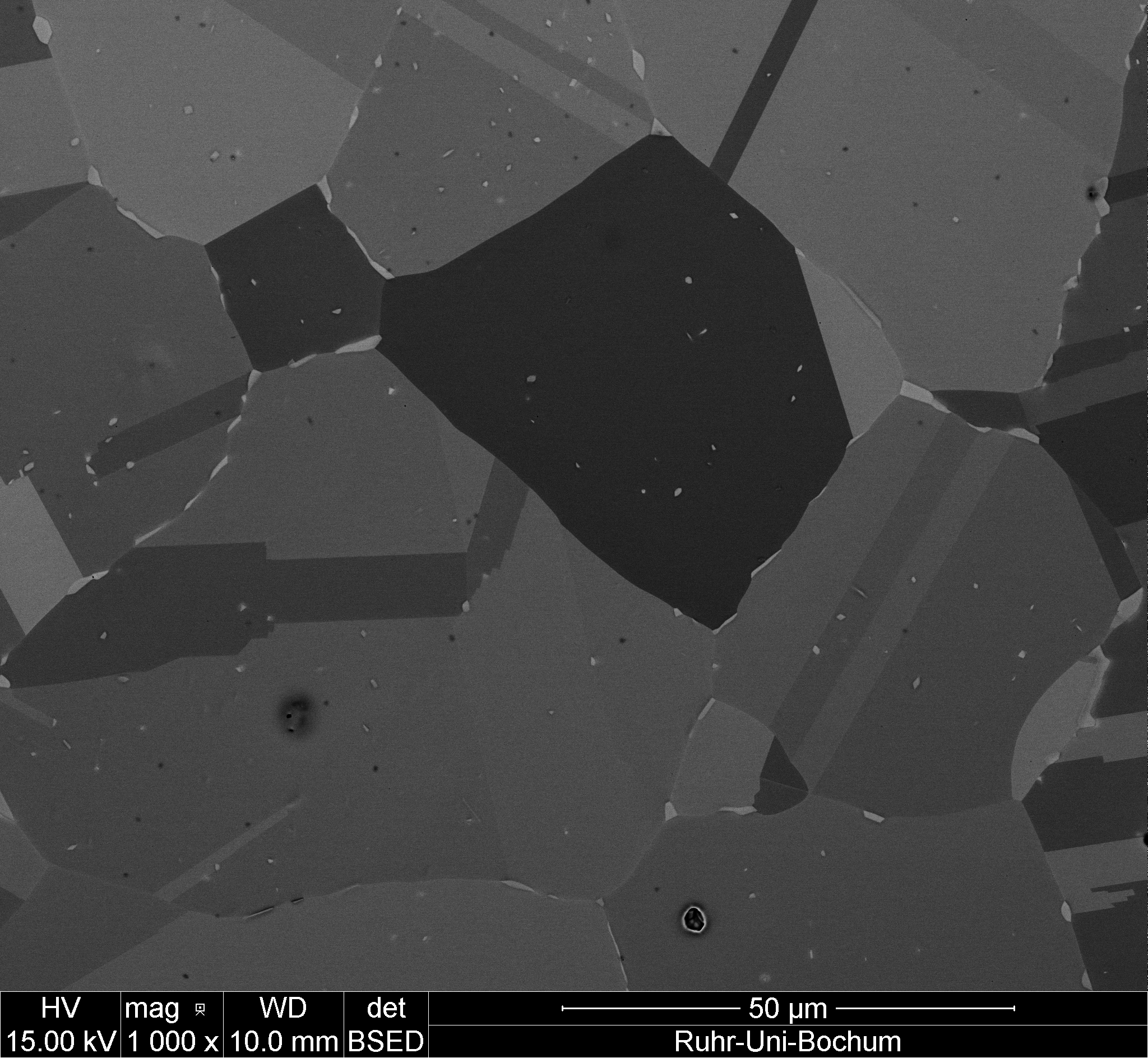

Supplement: Supplementary file 1 [file mmc1.zip › Upload_Data_in_Brief/BSE_microstructures/0900C_0001h/0900C_0001h_area2.tif]

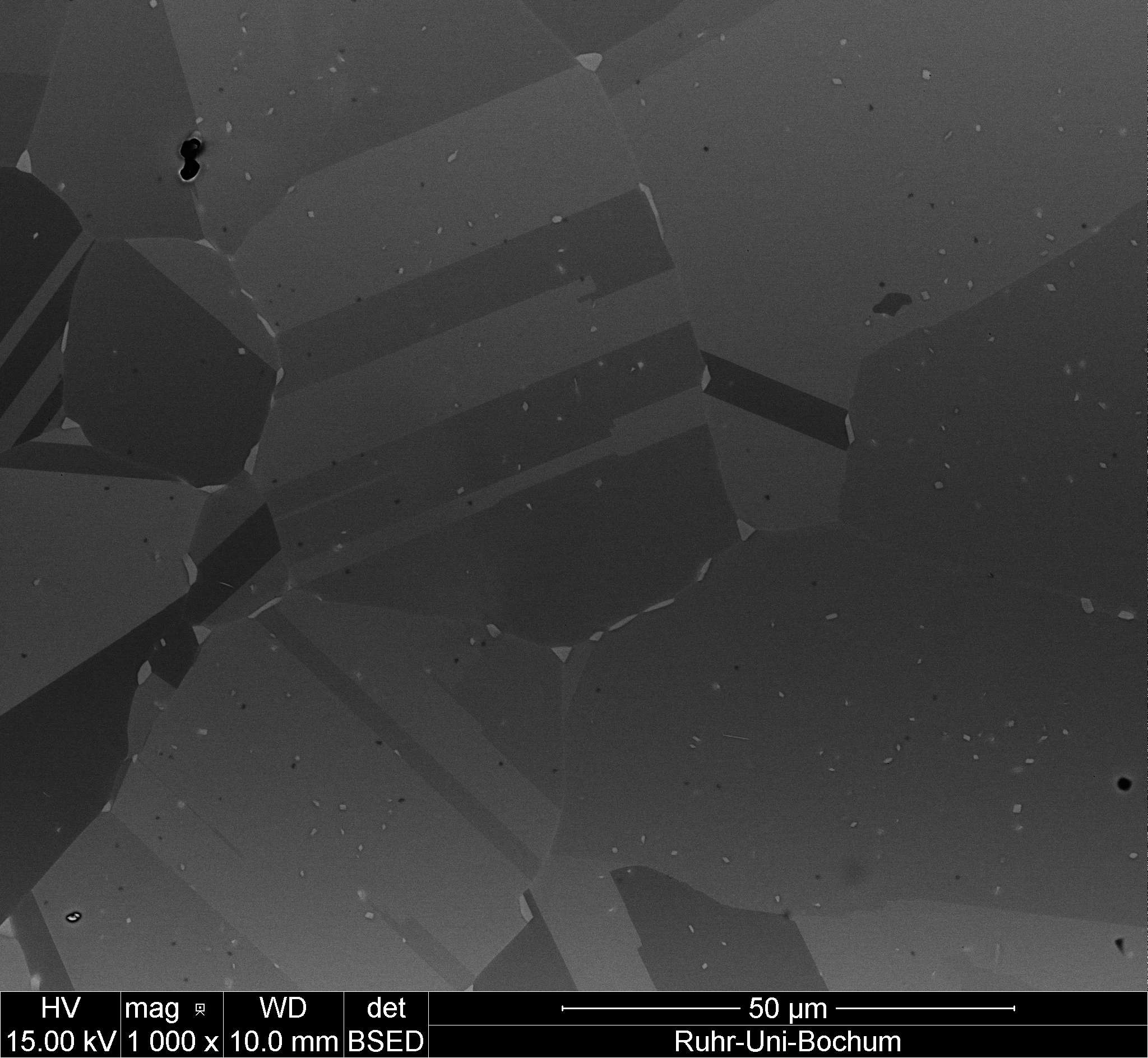

Supplement: Supplementary file 1 [file mmc1.zip › Upload_Data_in_Brief/BSE_microstructures/0900C_0001h/0900C_0001h_area3.tif]

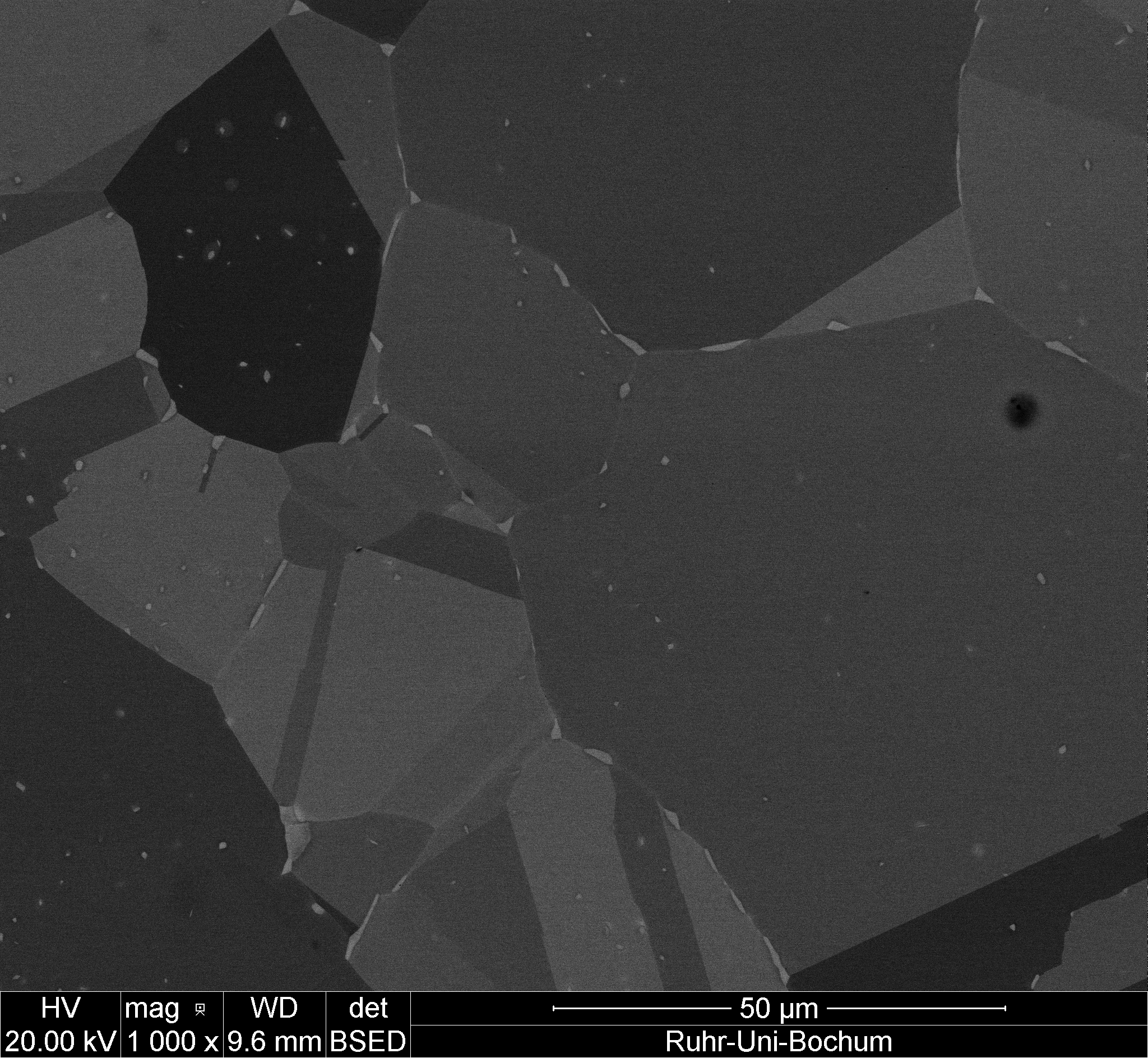

Supplement: Supplementary file 1 [file mmc1.zip › Upload_Data_in_Brief/BSE_microstructures/0900C_0001h/0900C_0001h_area4.tif]

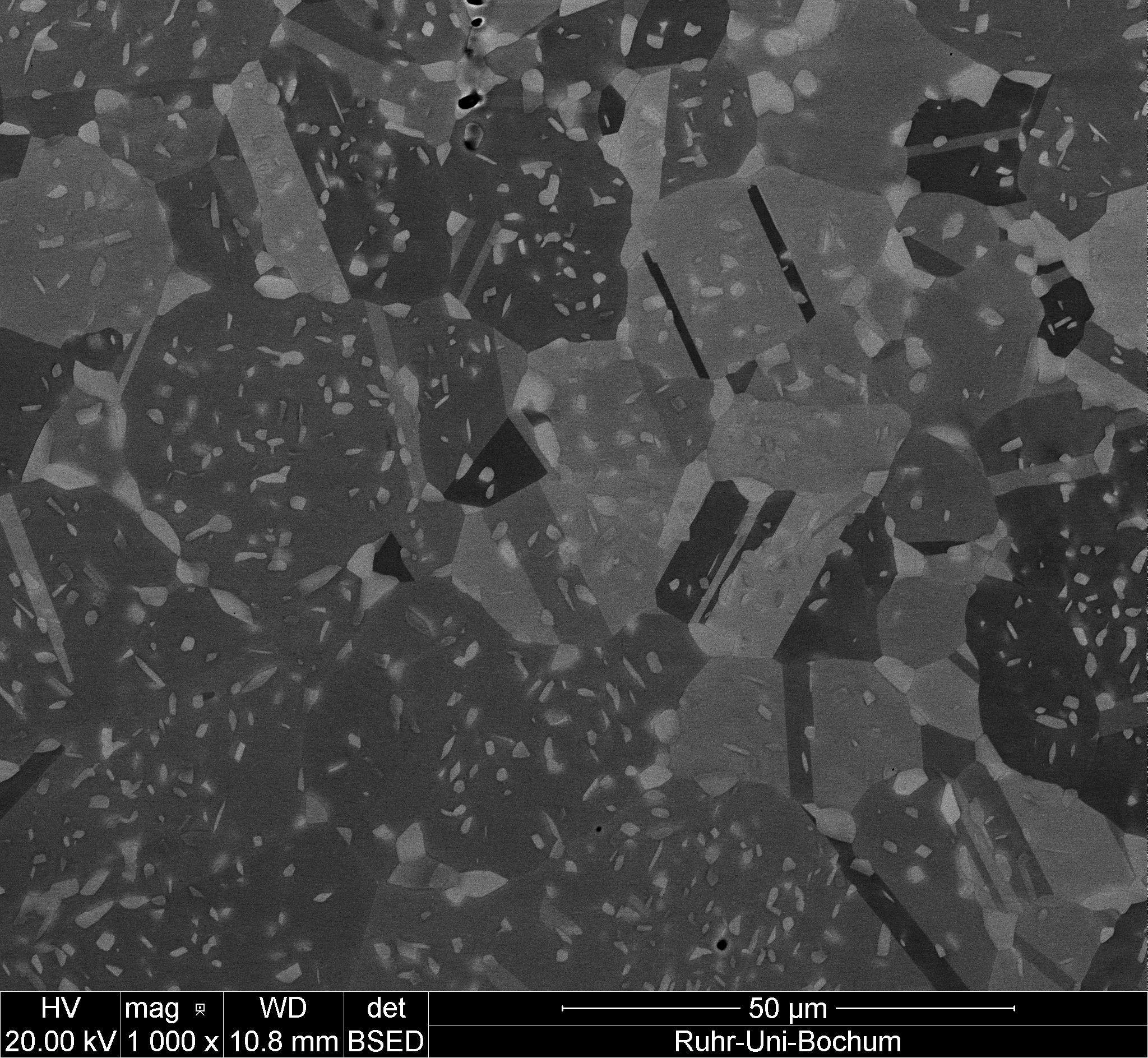

Supplement: Supplementary file 1 [file mmc1.zip › Upload_Data_in_Brief/BSE_microstructures/0900C_0010h/0900C_0010h_area1.tif]

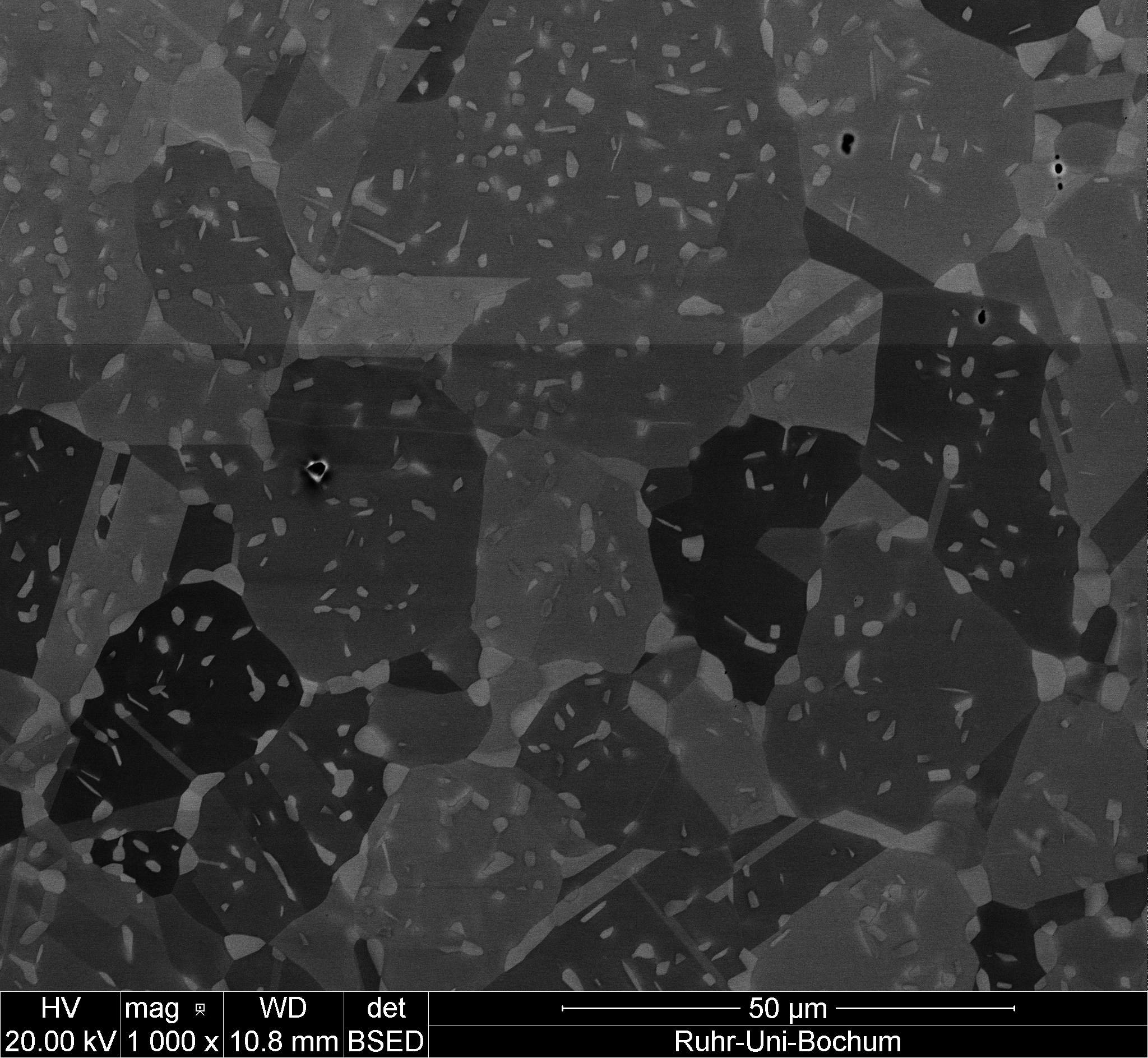

Supplement: Supplementary file 1 [file mmc1.zip › Upload_Data_in_Brief/BSE_microstructures/0900C_0010h/0900C_0010h_area2.tif]

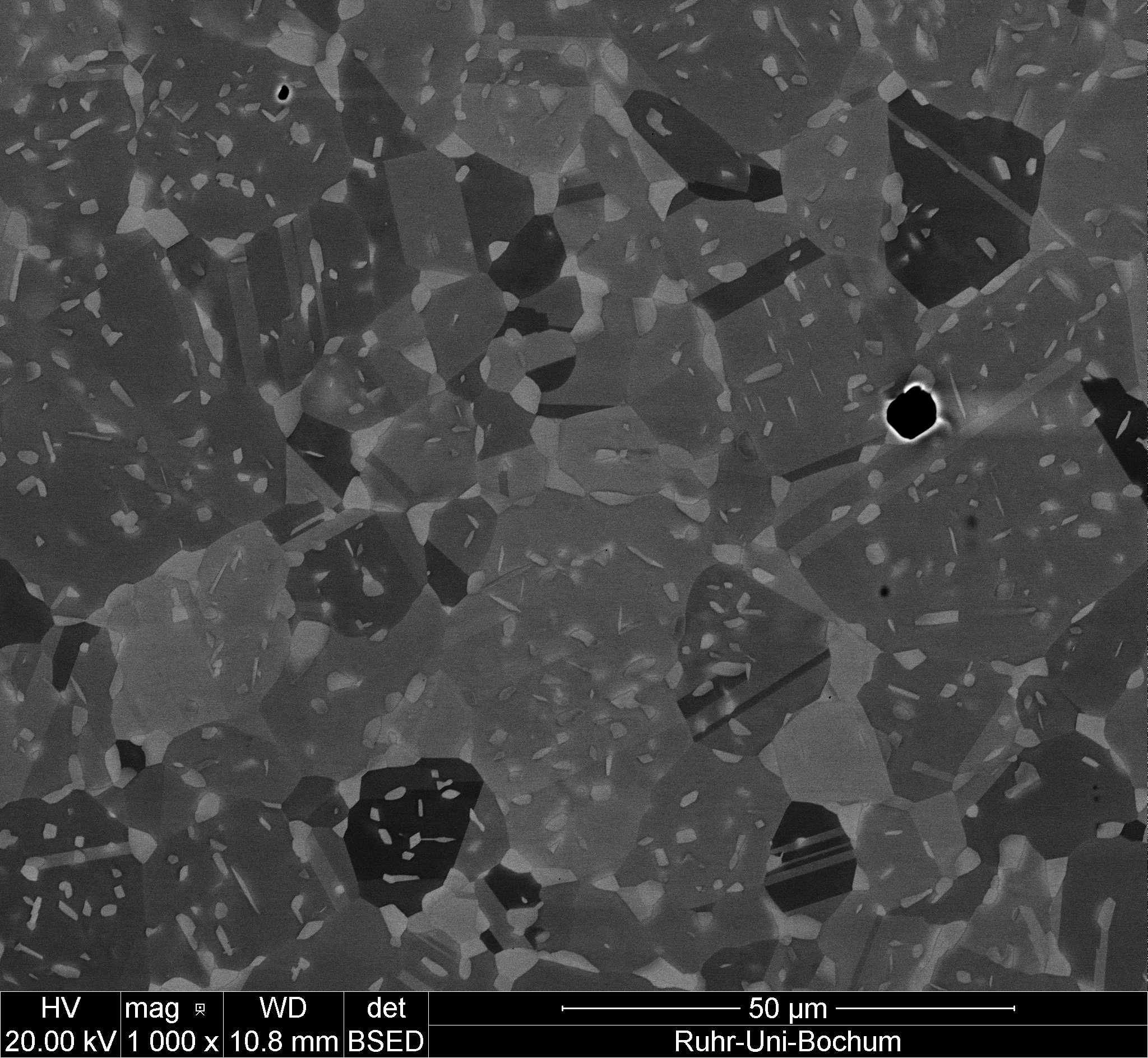

Supplement: Supplementary file 1 [file mmc1.zip › Upload_Data_in_Brief/BSE_microstructures/0900C_0010h/0900C_0010h_area3.tif]

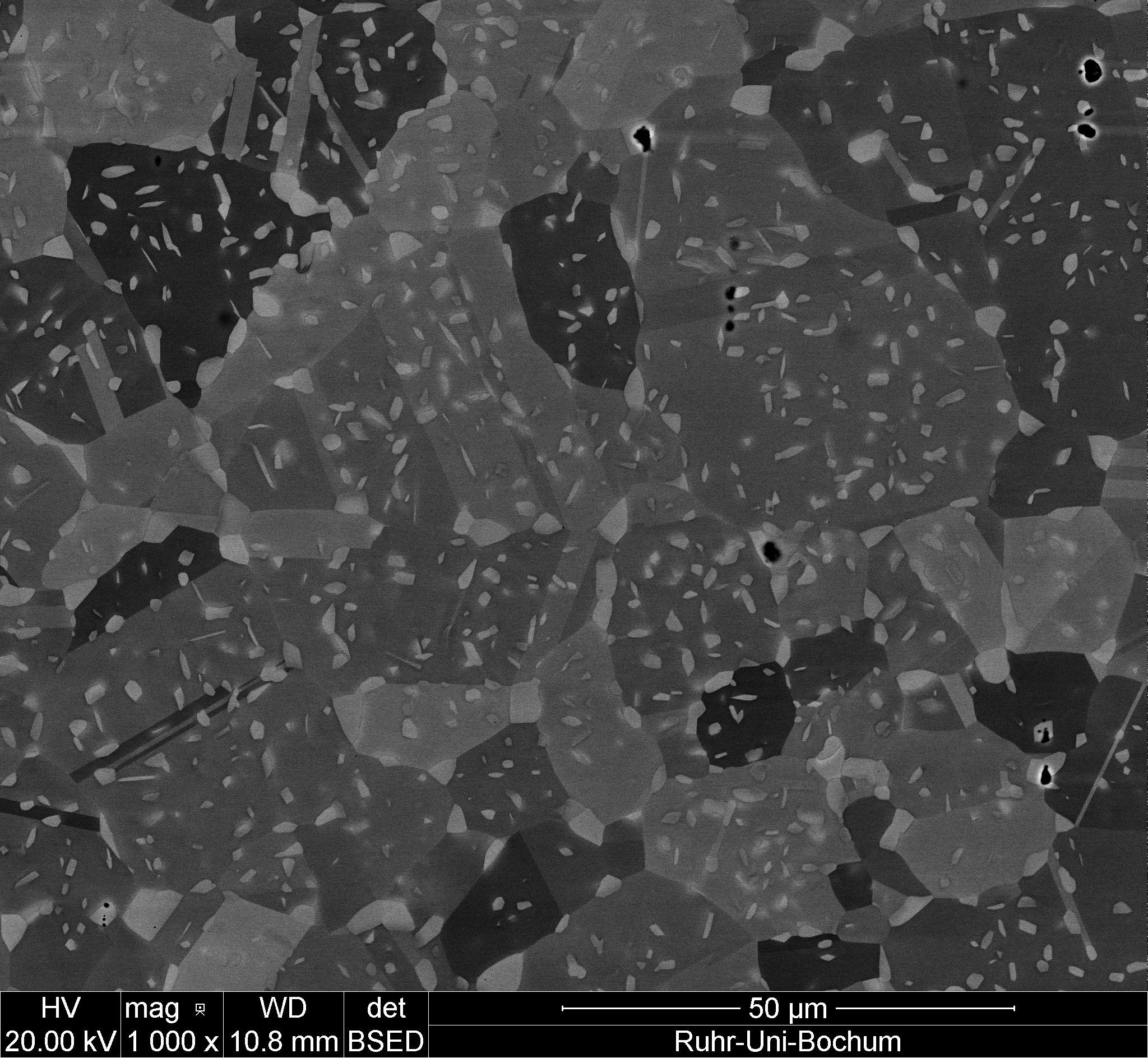

Supplement: Supplementary file 1 [file mmc1.zip › Upload_Data_in_Brief/BSE_microstructures/0900C_0010h/0900C_0010h_area4.tif]

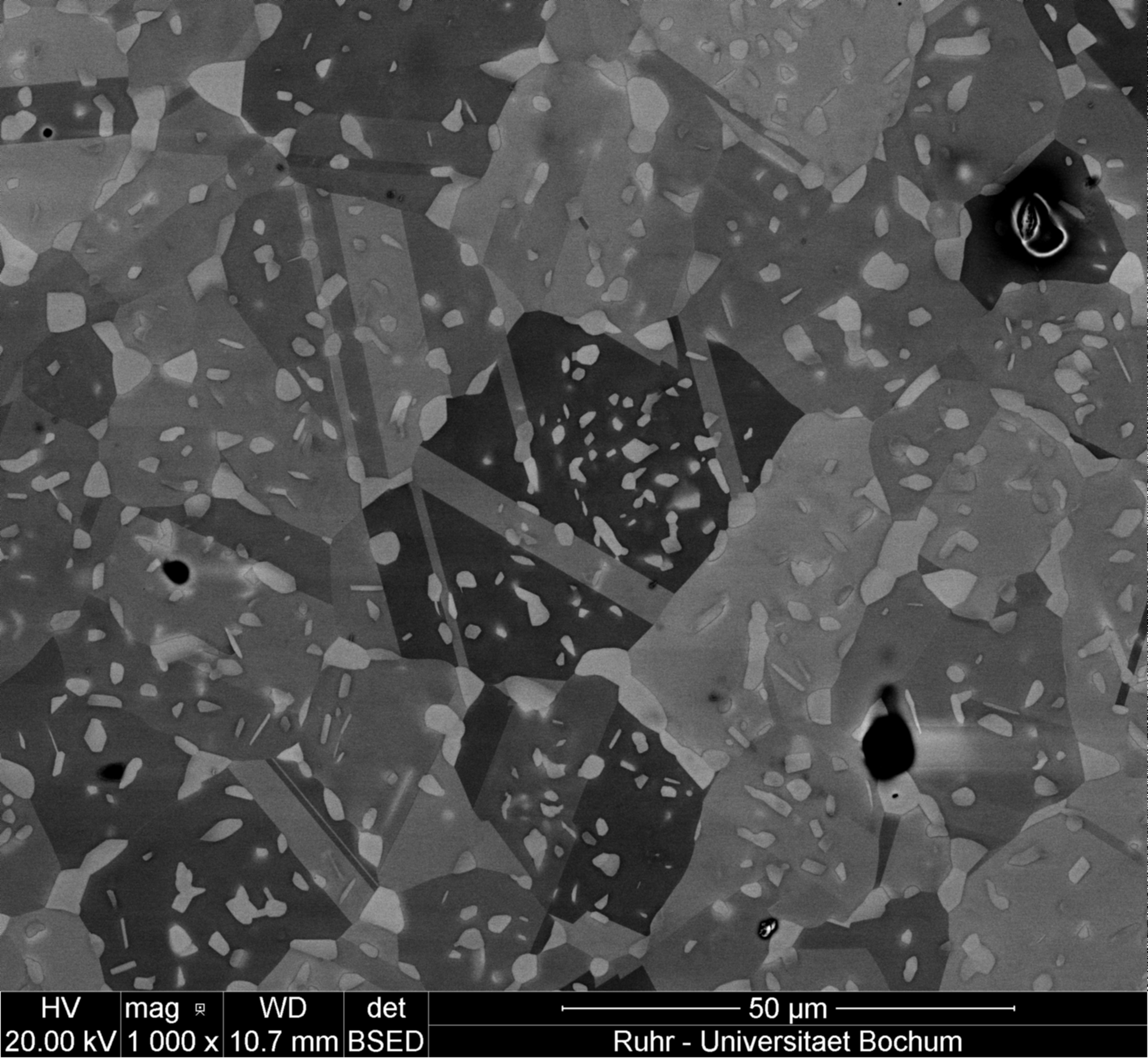

Supplement: Supplementary file 1 [file mmc1.zip › Upload_Data_in_Brief/BSE_microstructures/0900C_0100h/0900C_0100h_area1.tif]

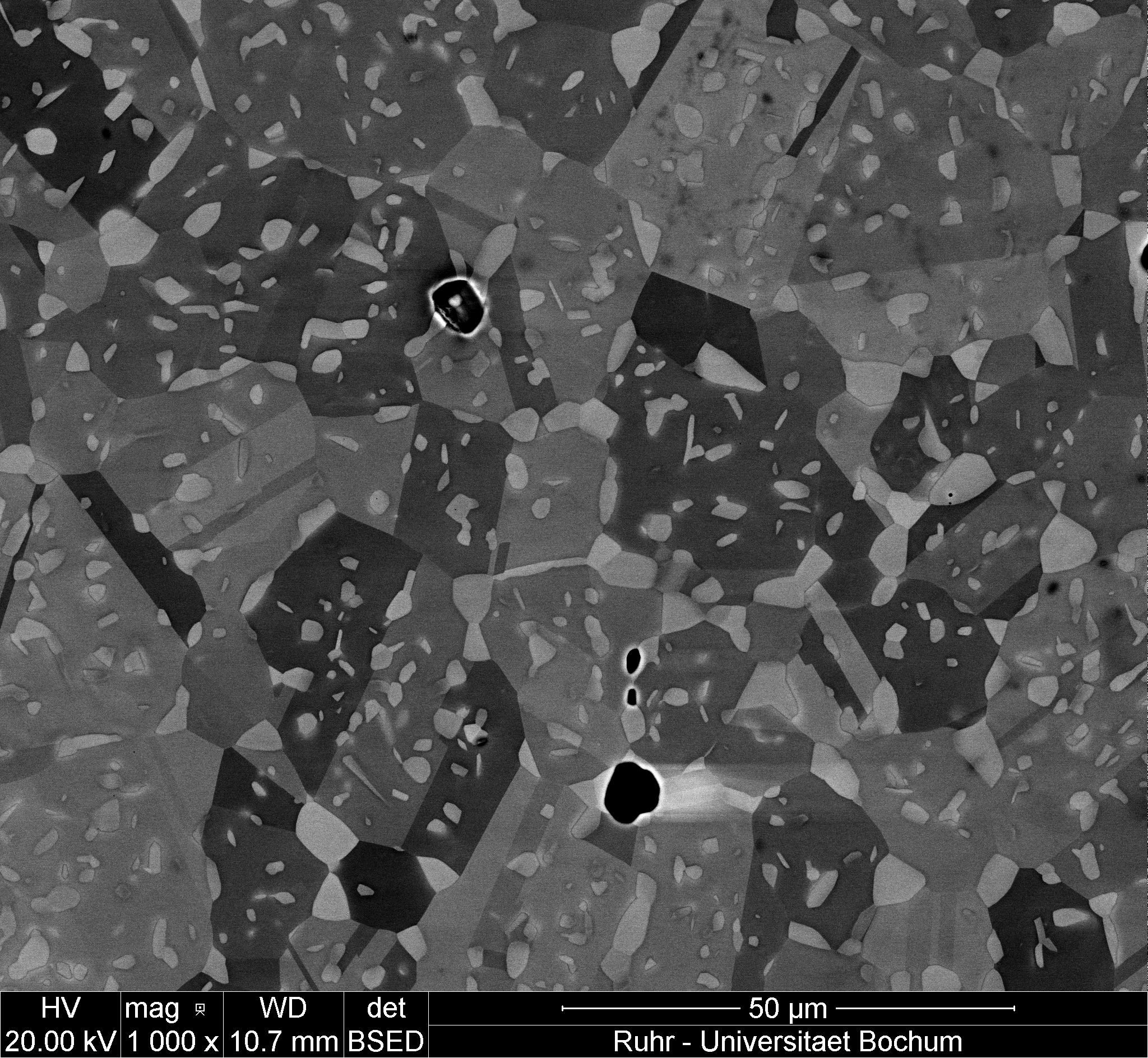

Supplement: Supplementary file 1 [file mmc1.zip › Upload_Data_in_Brief/BSE_microstructures/0900C_0100h/0900C_0100h_area2.tif]

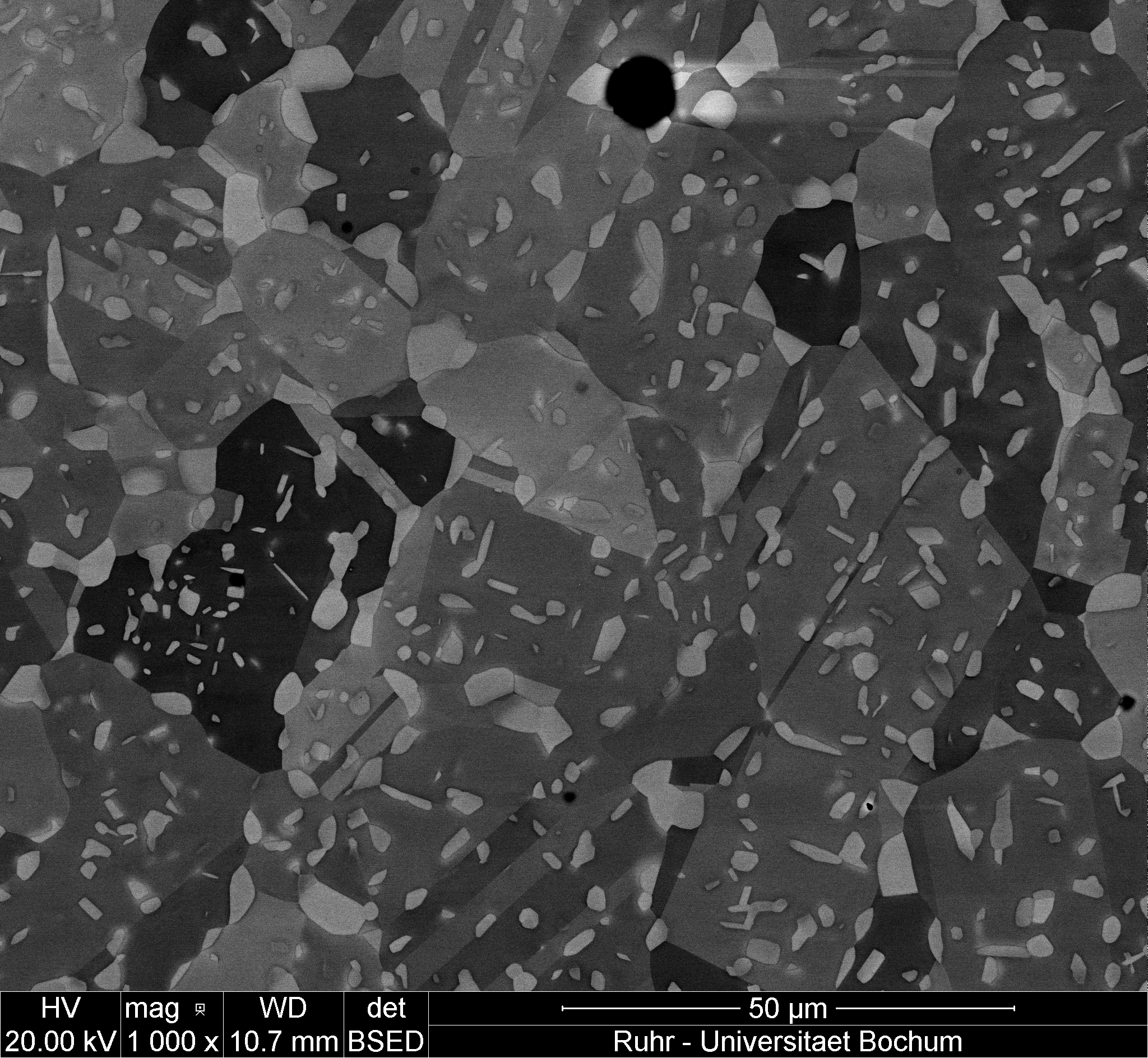

Supplement: Supplementary file 1 [file mmc1.zip › Upload_Data_in_Brief/BSE_microstructures/0900C_0100h/0900C_0100h_area3.tif]

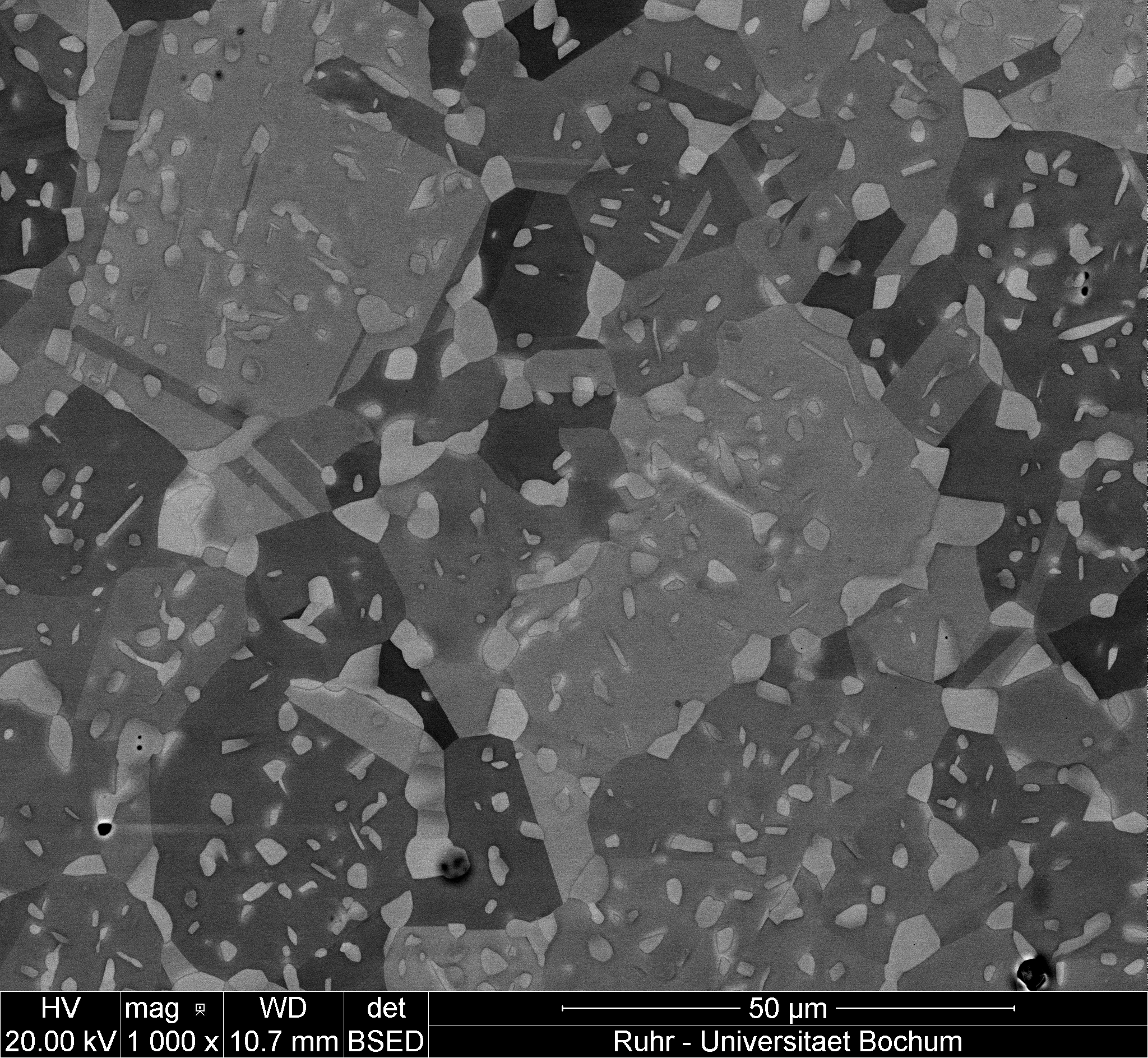

Supplement: Supplementary file 1 [file mmc1.zip › Upload_Data_in_Brief/BSE_microstructures/0900C_0100h/0900C_0100h_area4.tif]

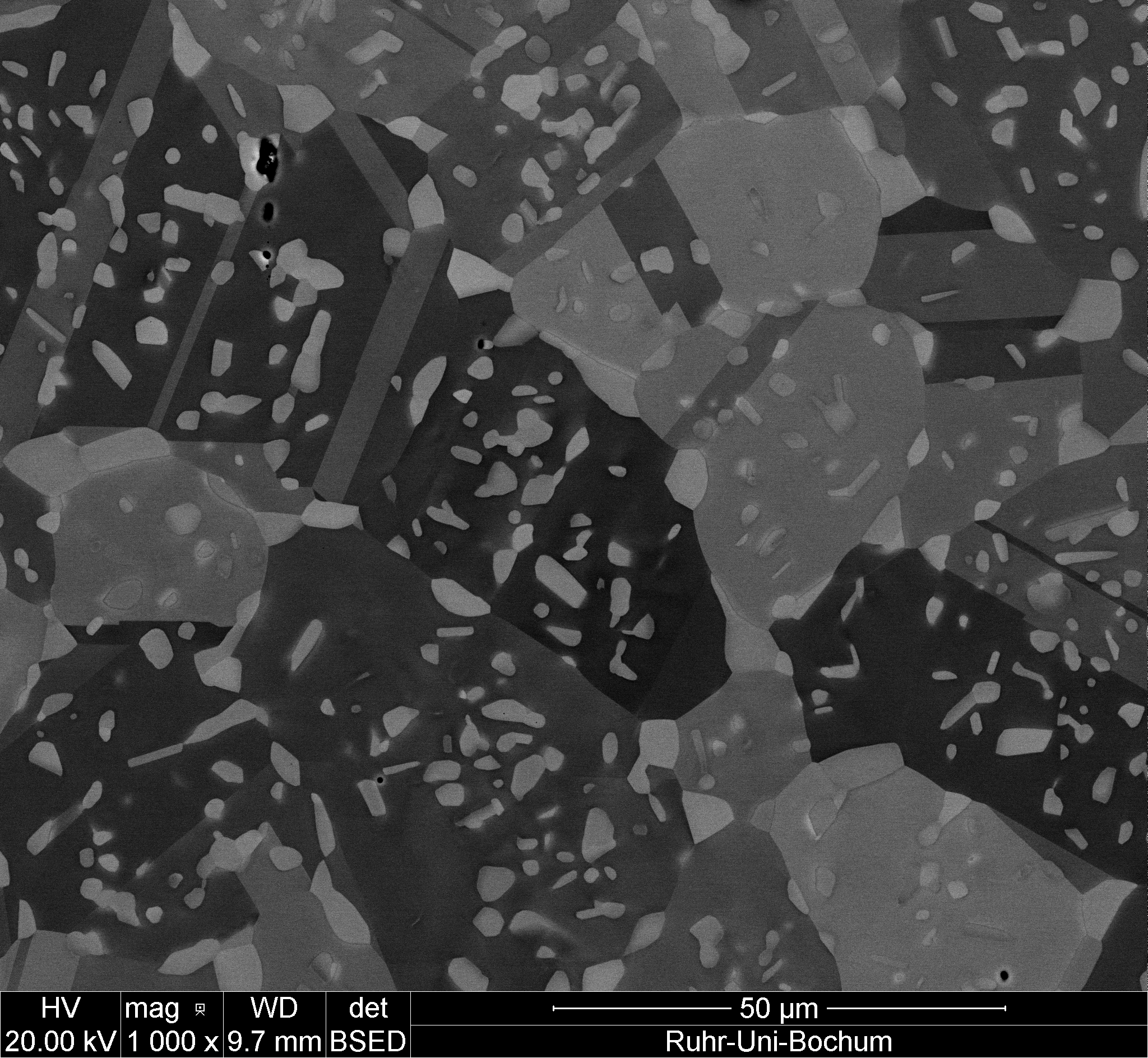

Supplement: Supplementary file 1 [file mmc1.zip › Upload_Data_in_Brief/BSE_microstructures/0900C_0500h/0900C_500h_area1.tif]

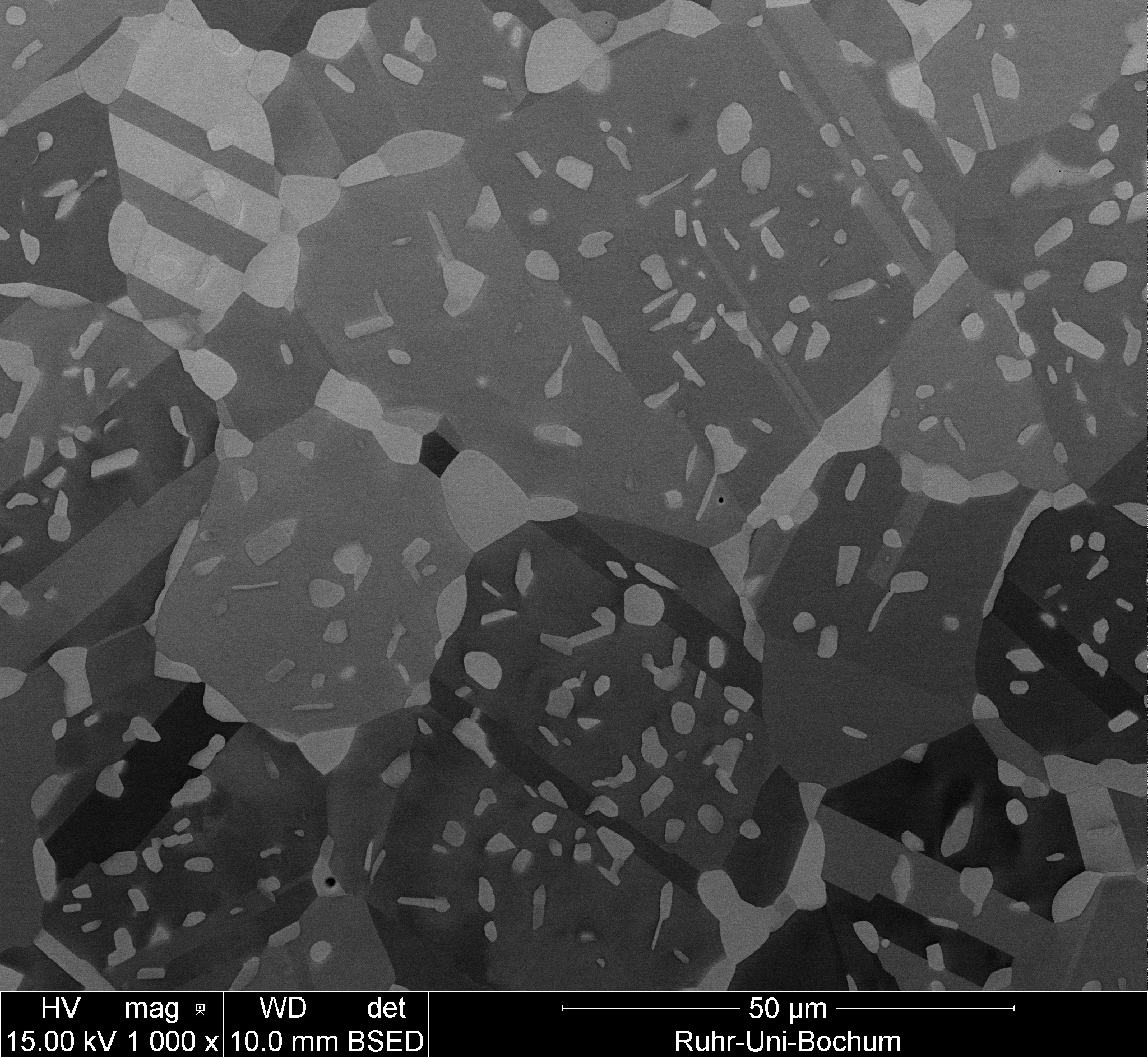

Supplement: Supplementary file 1 [file mmc1.zip › Upload_Data_in_Brief/BSE_microstructures/0900C_0500h/0900C_500h_area2.tif]

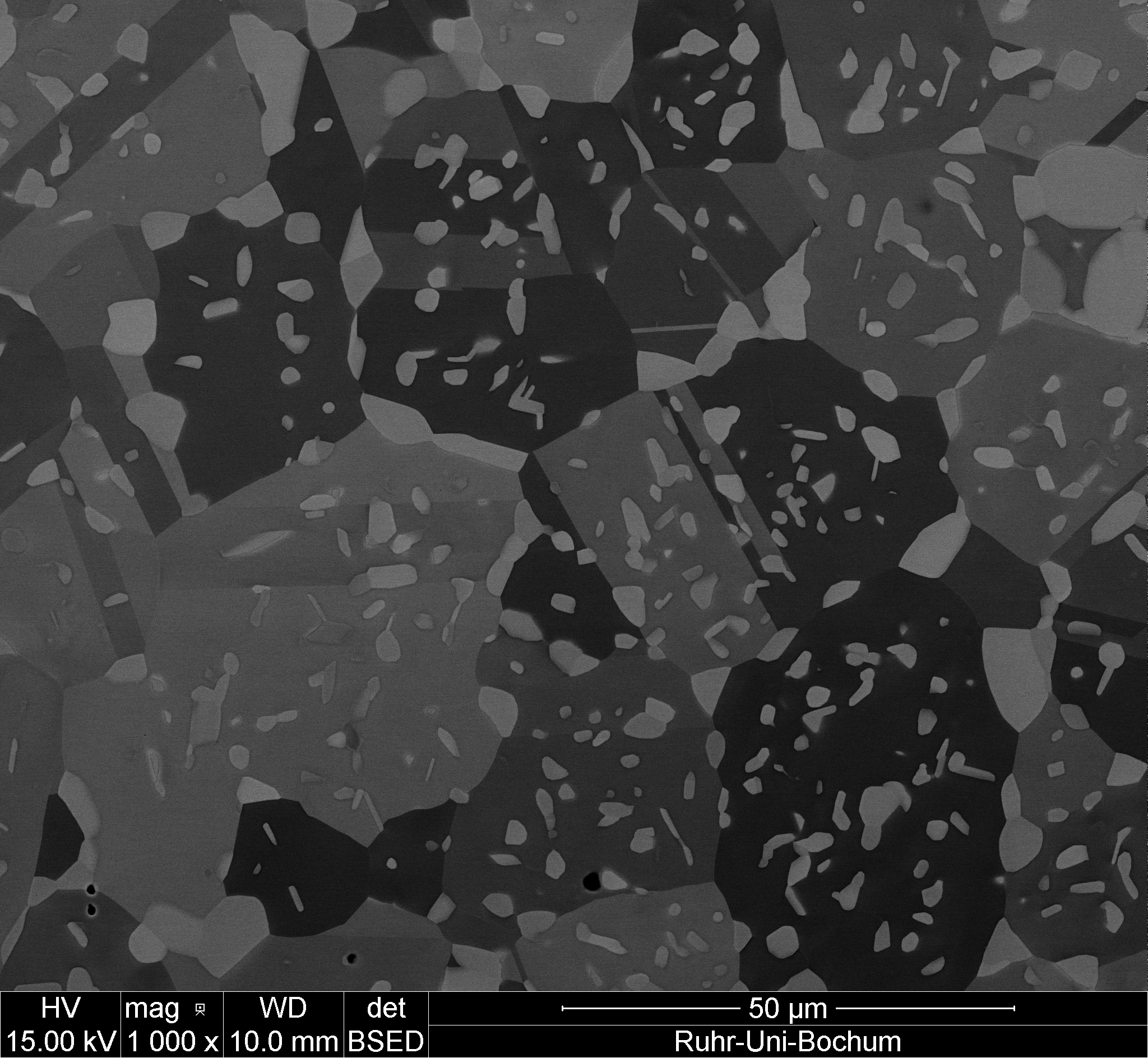

Supplement: Supplementary file 1 [file mmc1.zip › Upload_Data_in_Brief/BSE_microstructures/0900C_0500h/0900C_500h_area3.tif]

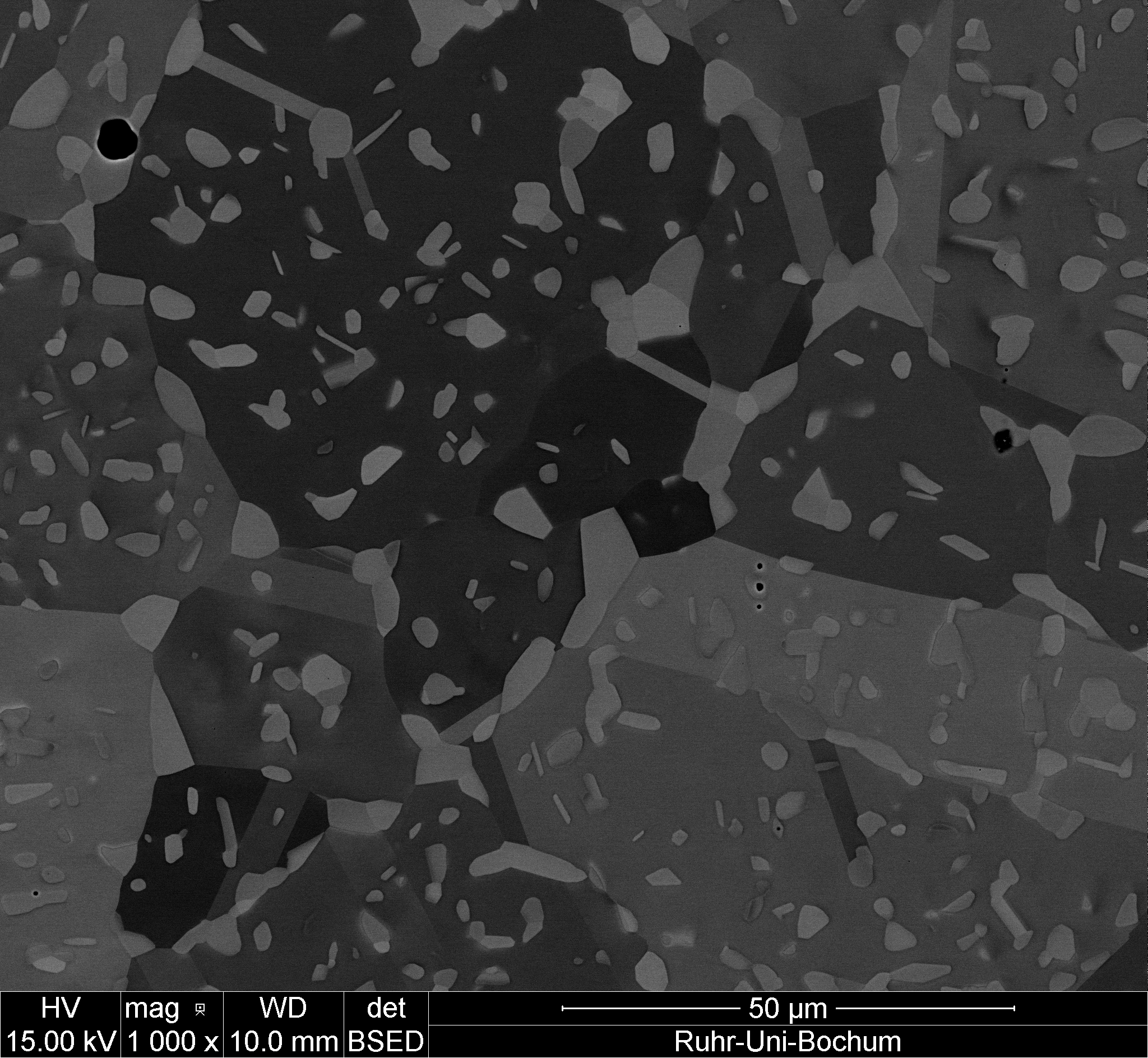

Supplement: Supplementary file 1 [file mmc1.zip › Upload_Data_in_Brief/BSE_microstructures/0900C_0500h/0900C_500h_area4.tif]

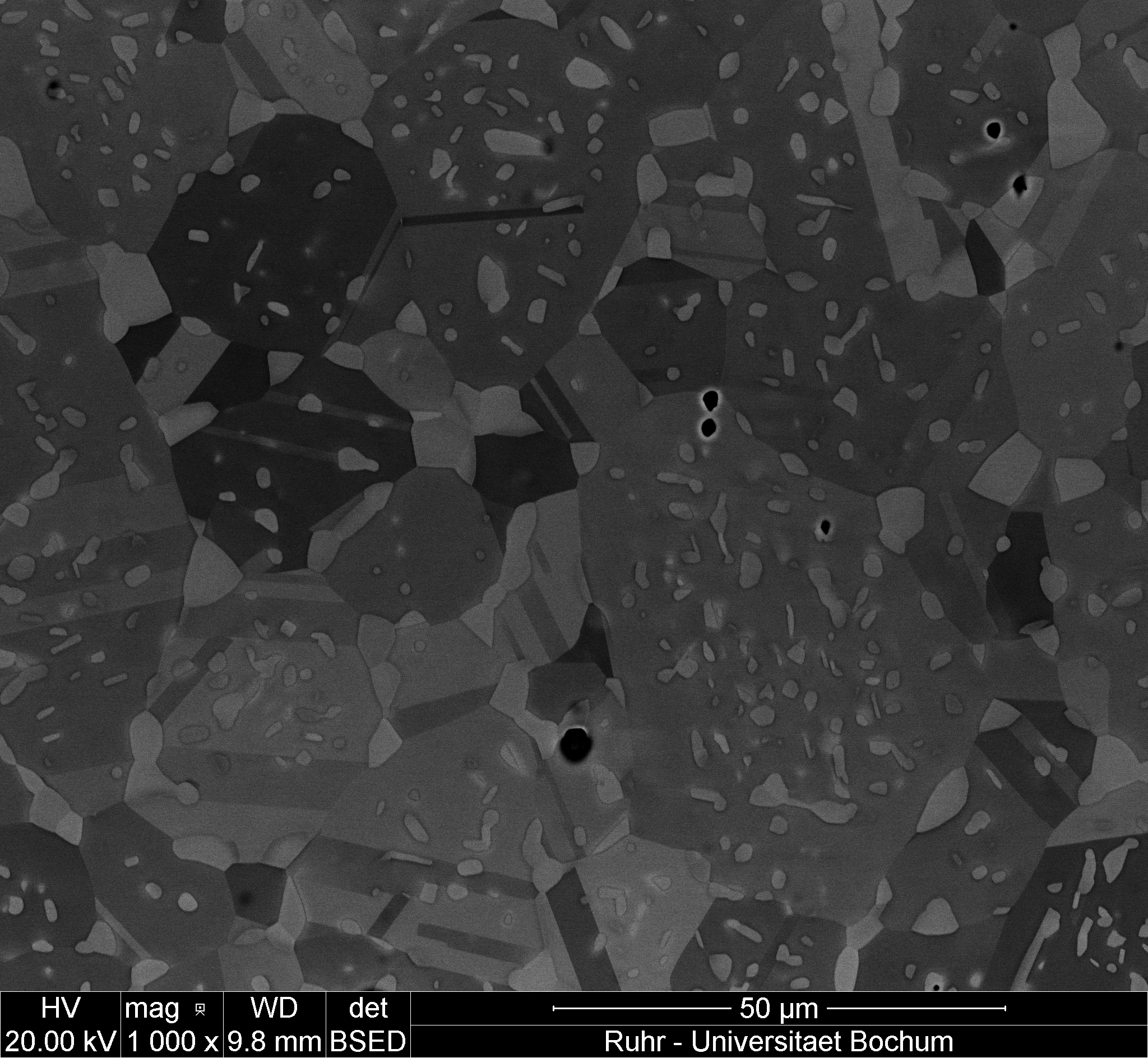

Supplement: Supplementary file 1 [file mmc1.zip › Upload_Data_in_Brief/BSE_microstructures/0900C_1000h/0900C_1000h_area1.tif]

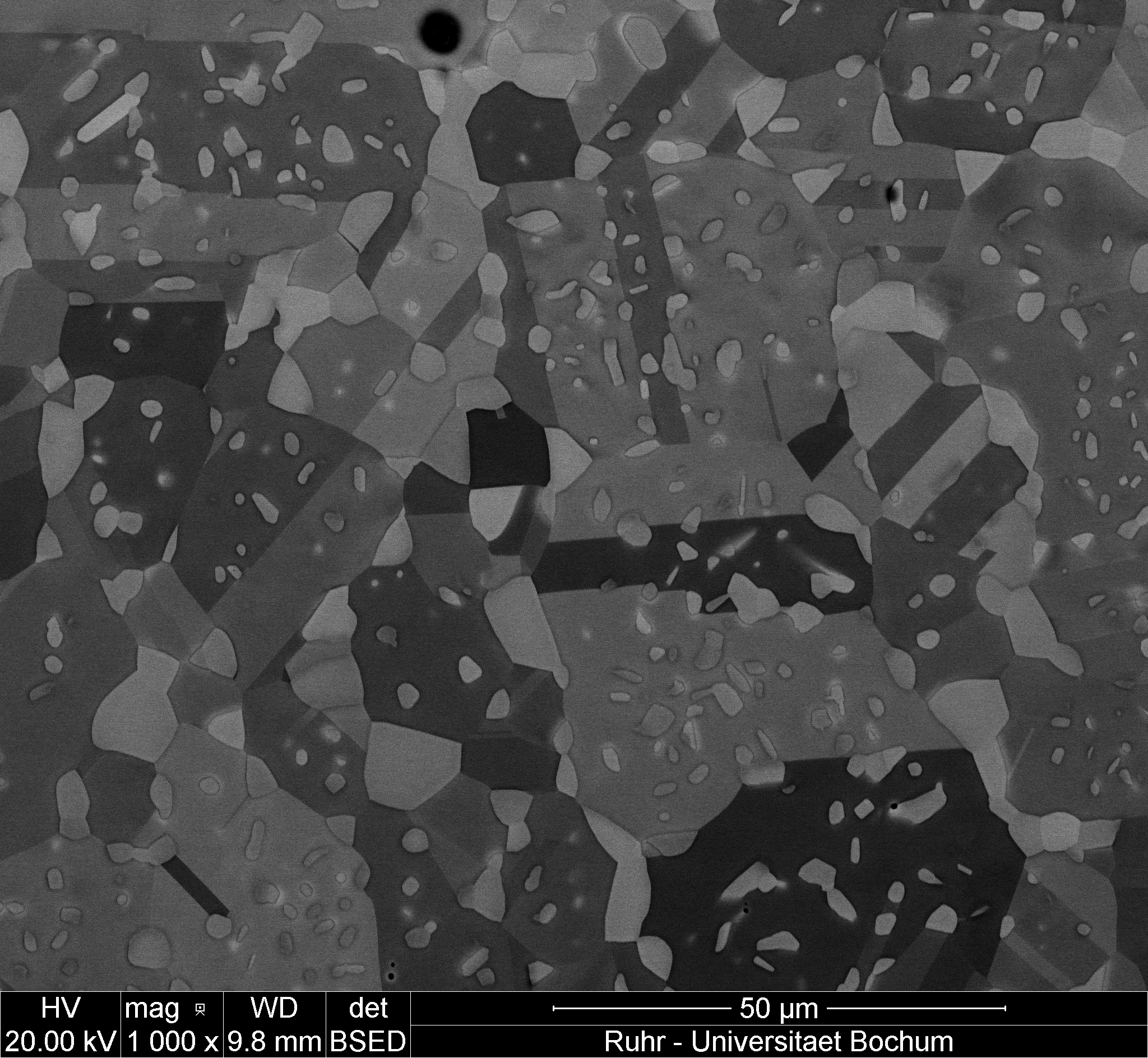

Supplement: Supplementary file 1 [file mmc1.zip › Upload_Data_in_Brief/BSE_microstructures/0900C_1000h/0900C_1000h_area2.tif]

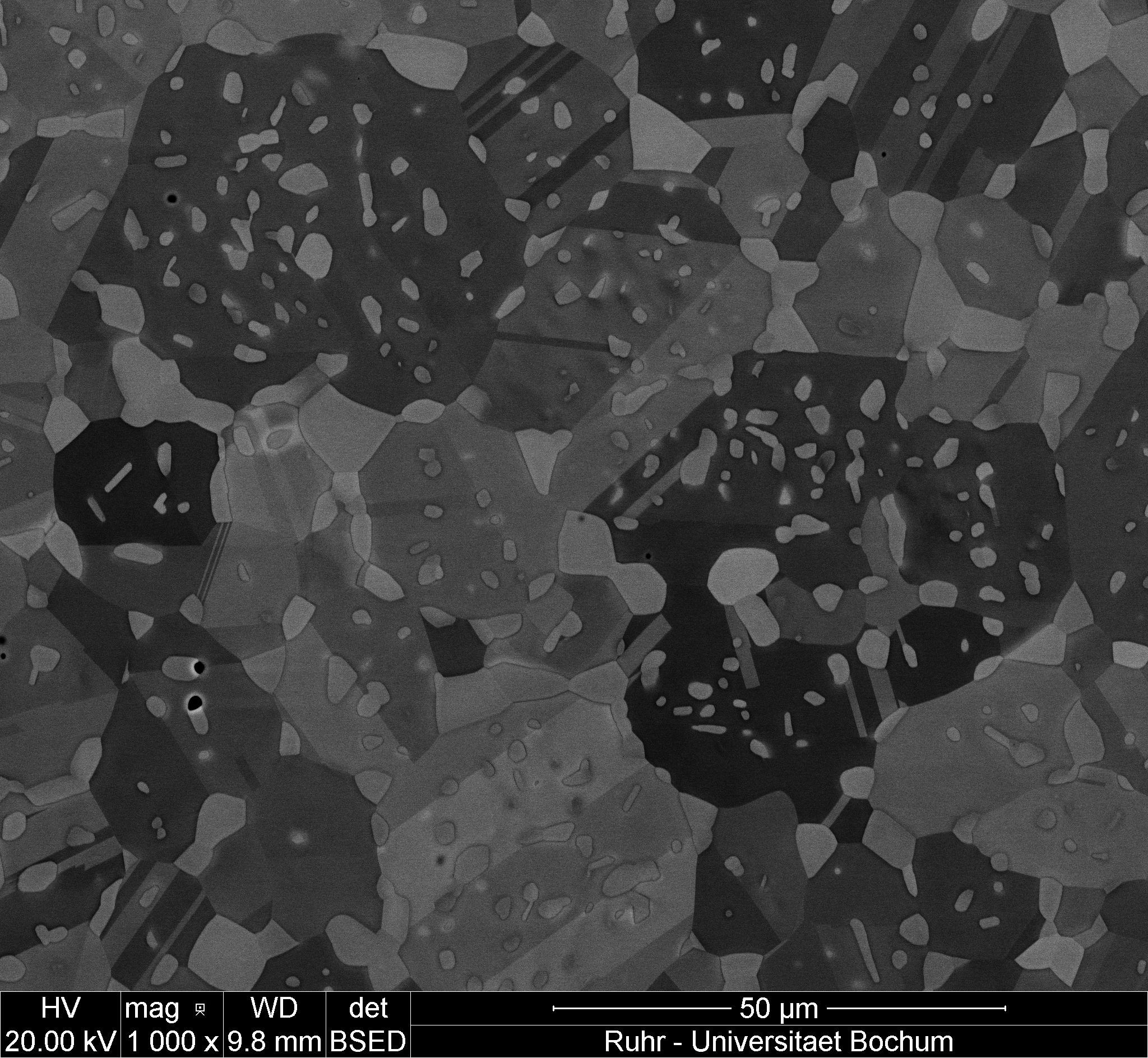

Supplement: Supplementary file 1 [file mmc1.zip › Upload_Data_in_Brief/BSE_microstructures/0900C_1000h/0900C_1000h_area3.tif]

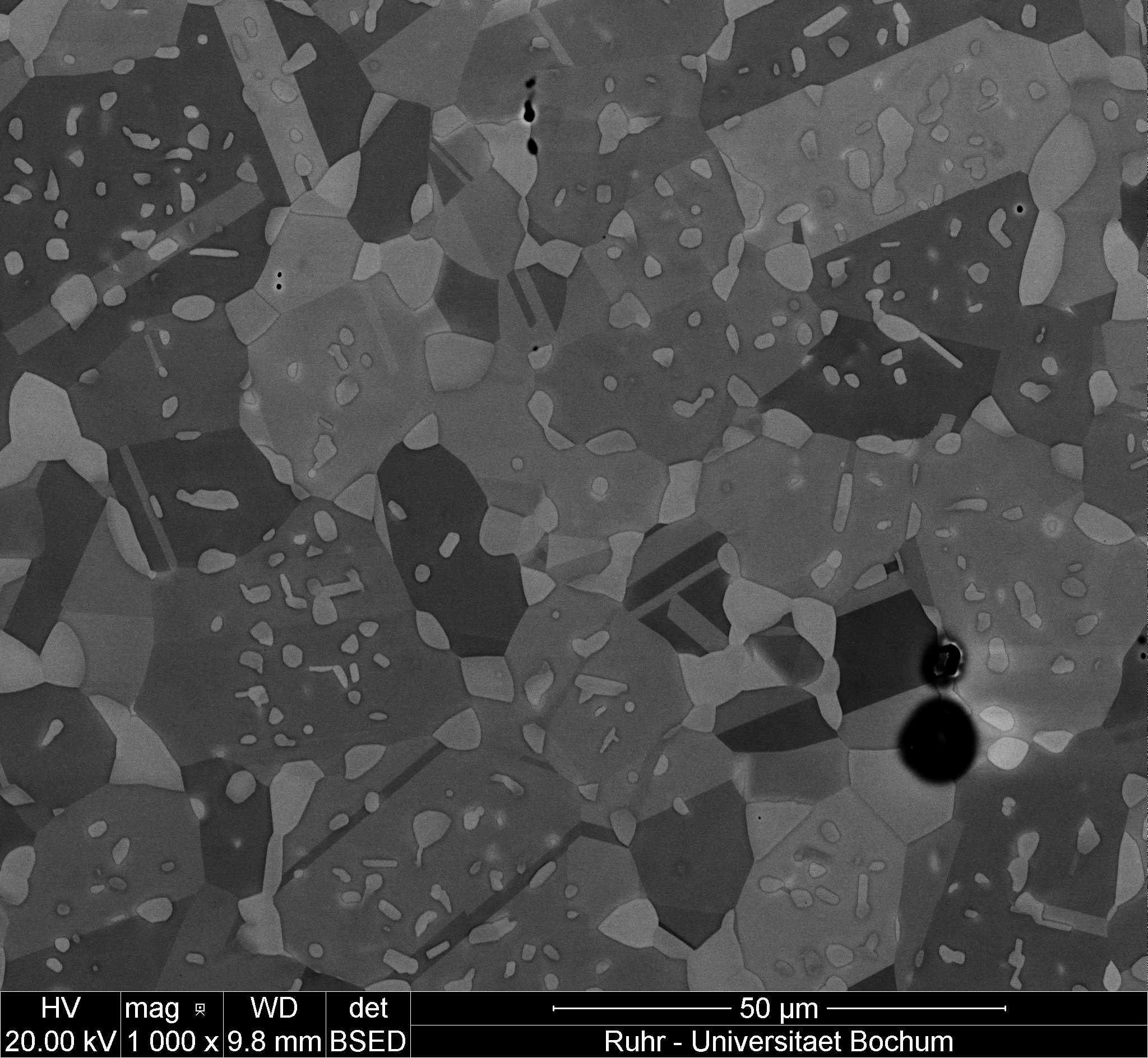

Supplement: Supplementary file 1 [file mmc1.zip › Upload_Data_in_Brief/BSE_microstructures/0900C_1000h/0900C_1000h_area4.tif]

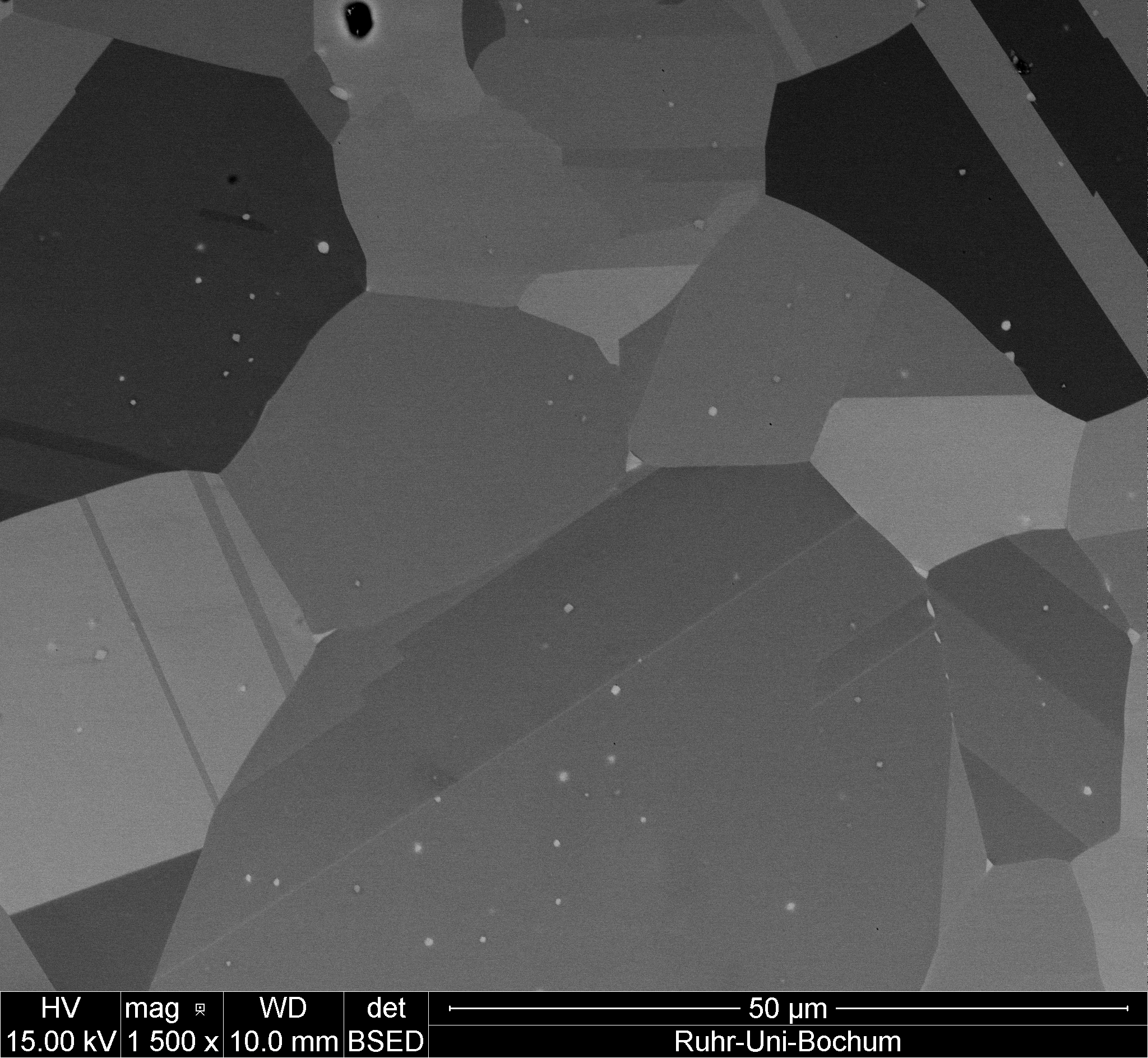

Supplement: Supplementary file 1 [file mmc1.zip › Upload_Data_in_Brief/BSE_microstructures/1000C_0.05h/1000C_0.05h_area1.tif]

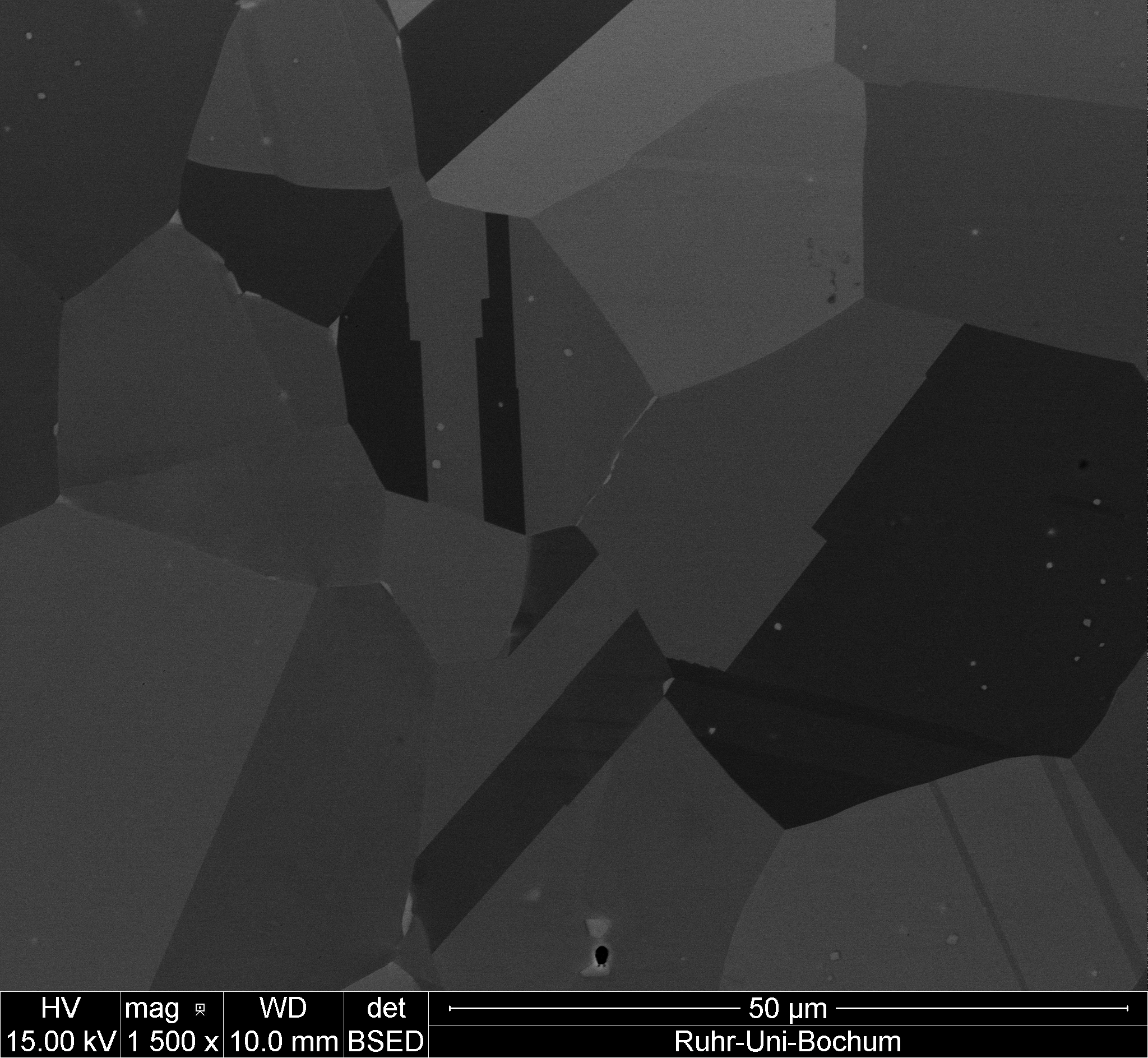

Supplement: Supplementary file 1 [file mmc1.zip › Upload_Data_in_Brief/BSE_microstructures/1000C_0.05h/1000C_0.05h_area2.tif]

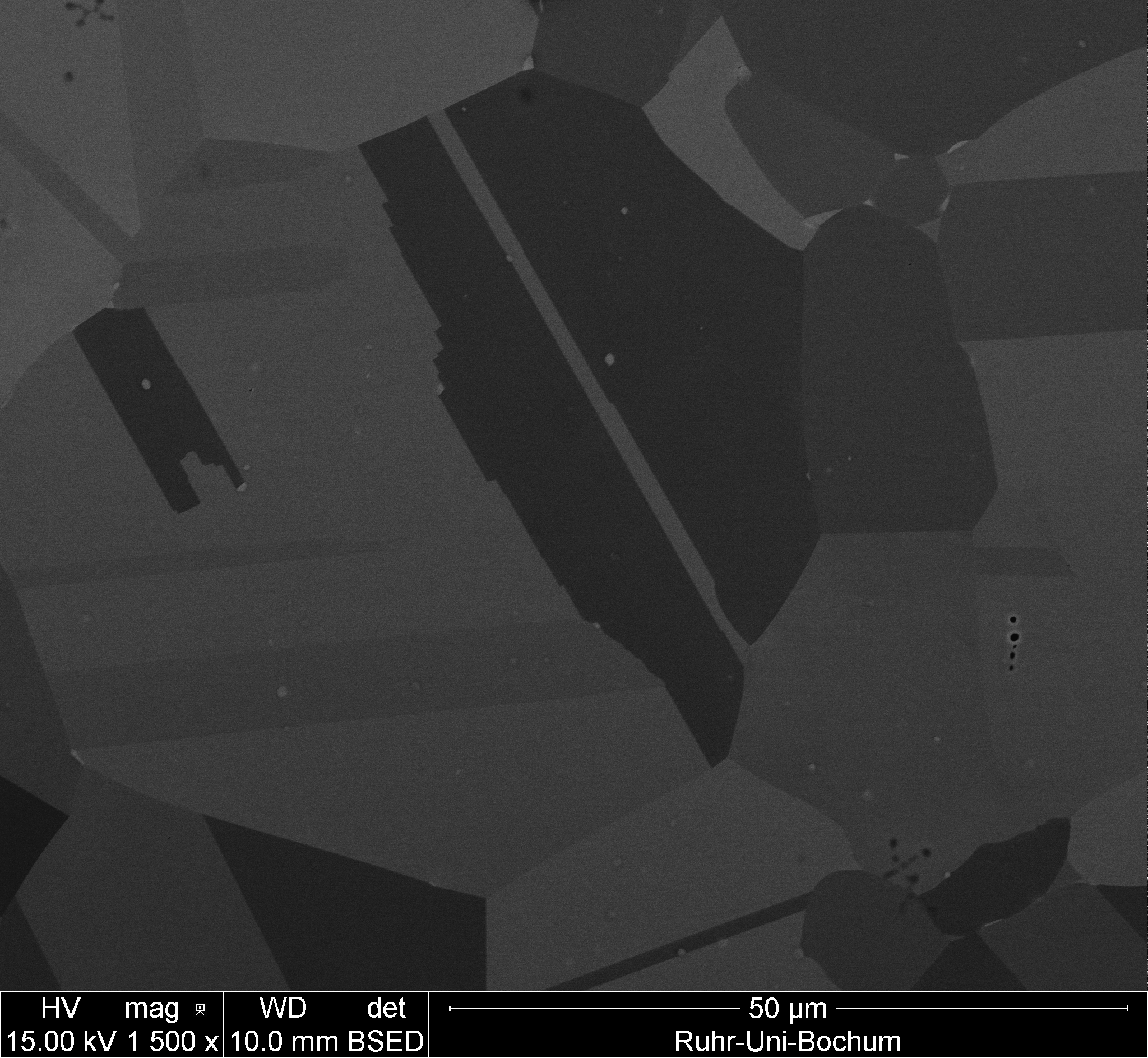

Supplement: Supplementary file 1 [file mmc1.zip › Upload_Data_in_Brief/BSE_microstructures/1000C_0.05h/1000C_0.05h_area3.tif]

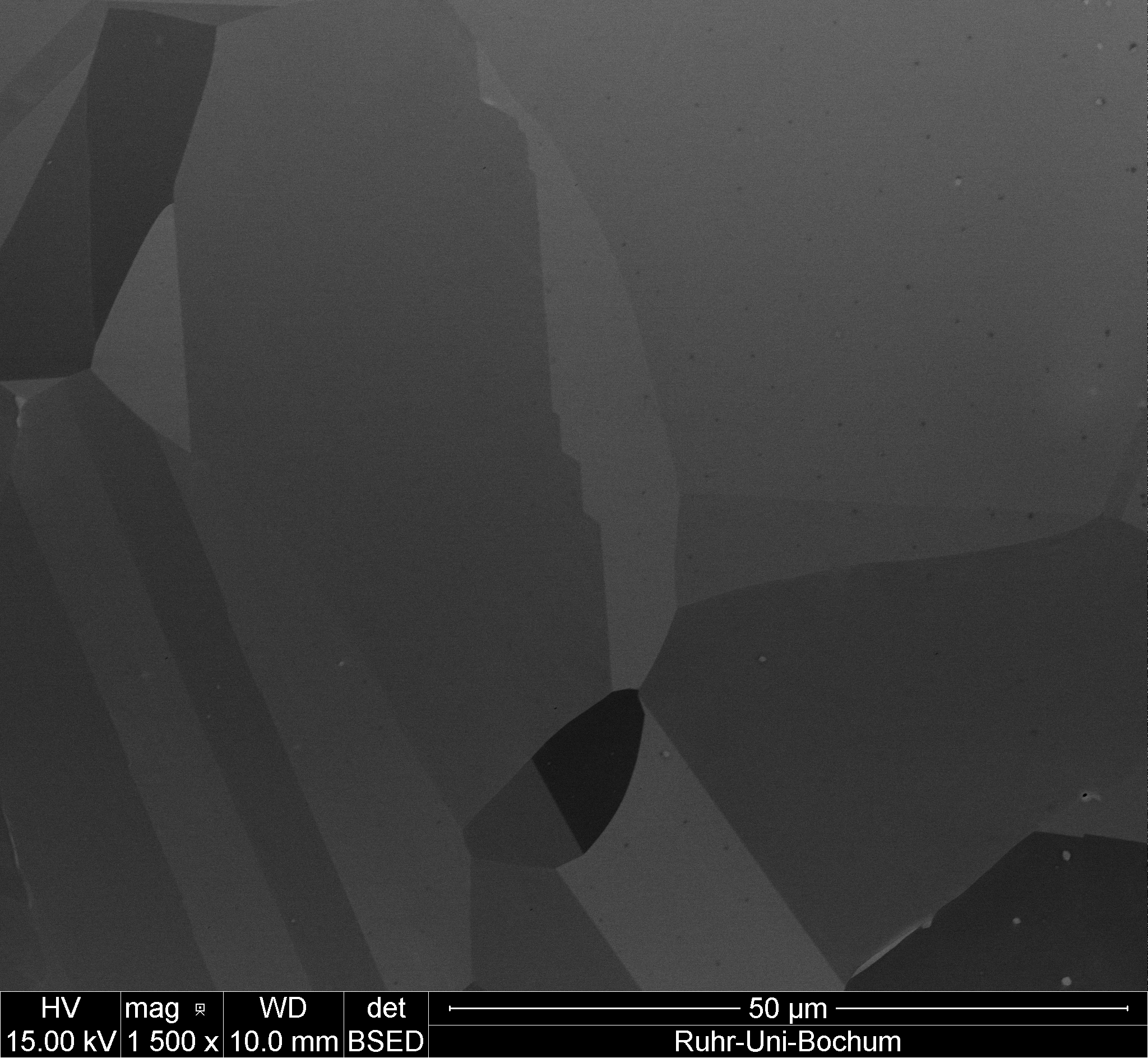

Supplement: Supplementary file 1 [file mmc1.zip › Upload_Data_in_Brief/BSE_microstructures/1000C_0.05h/1000C_0.05h_area4.tif]

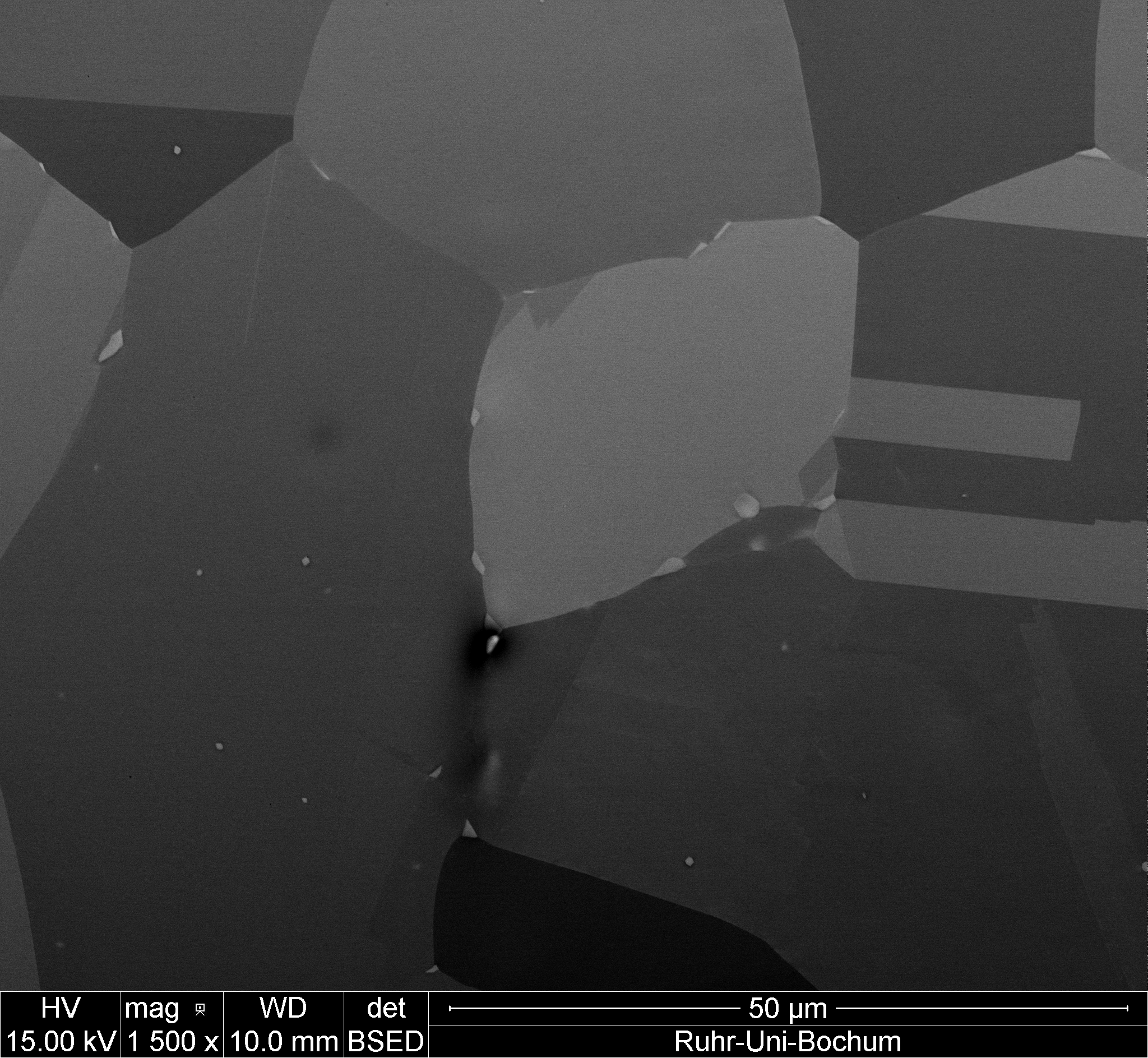

Supplement: Supplementary file 1 [file mmc1.zip › Upload_Data_in_Brief/BSE_microstructures/1000C_0.10h/1000C_0.10h_area1.tif]

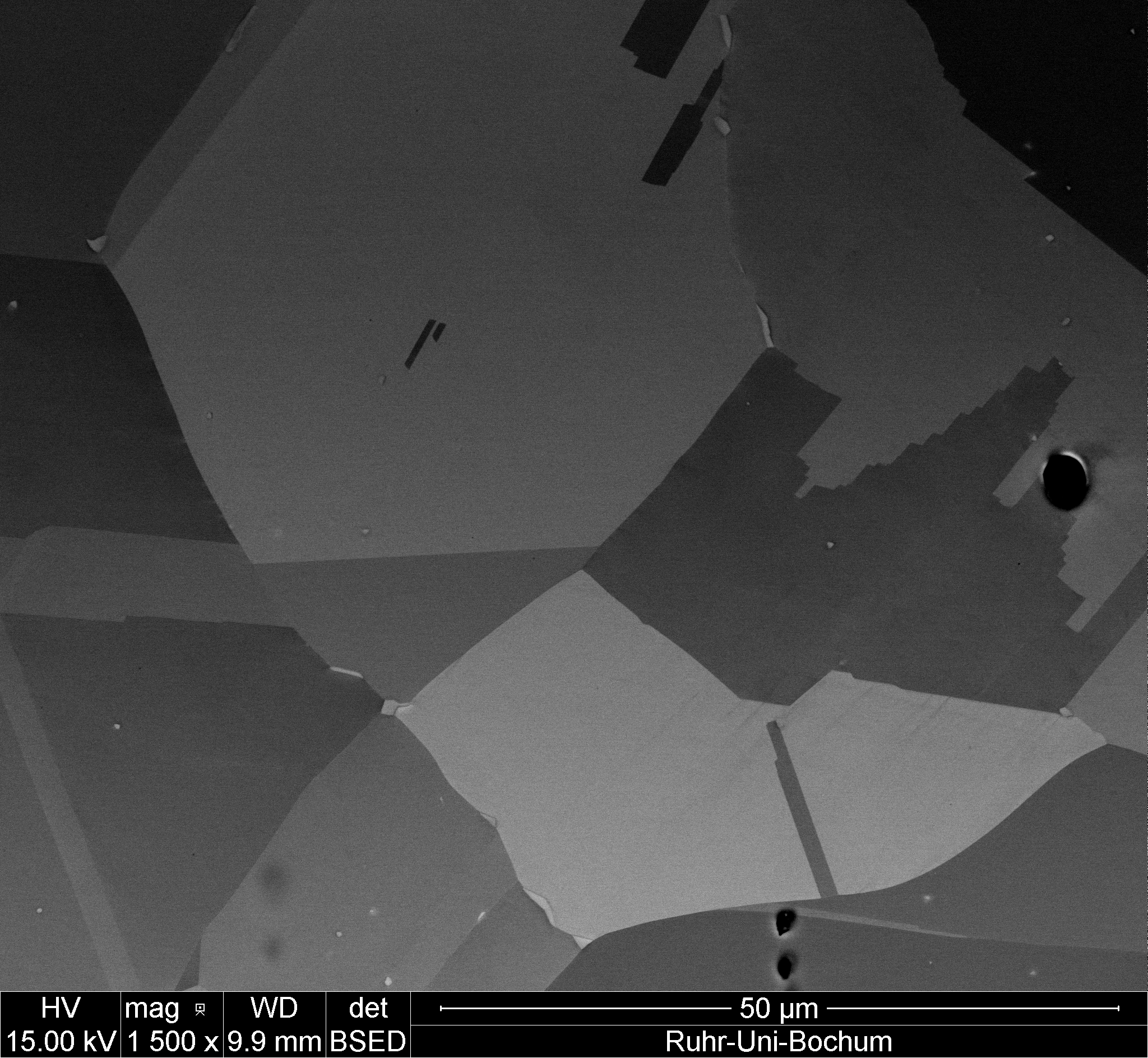

Supplement: Supplementary file 1 [file mmc1.zip › Upload_Data_in_Brief/BSE_microstructures/1000C_0.10h/1000C_0.10h_area2.tif]

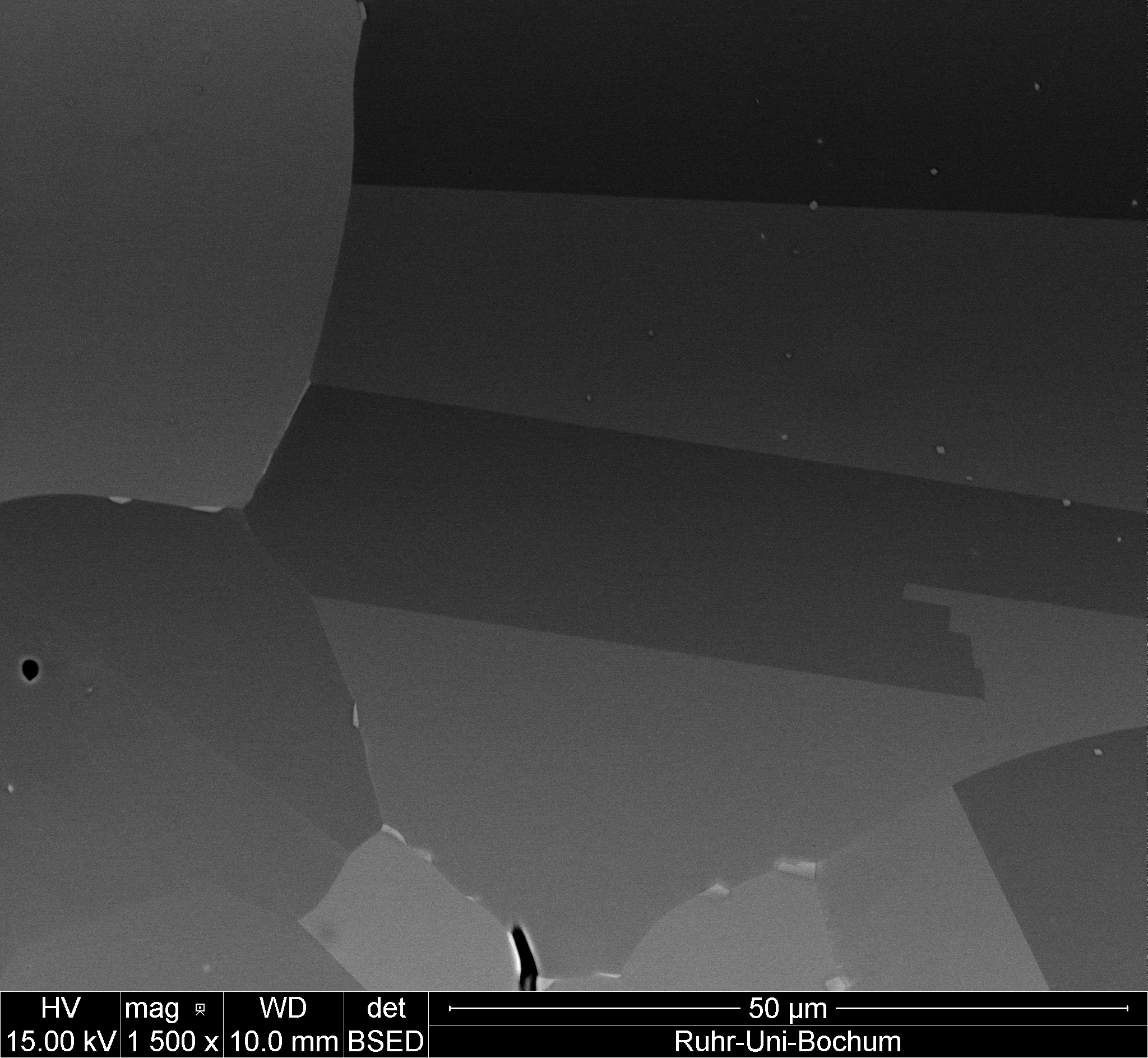

Supplement: Supplementary file 1 [file mmc1.zip › Upload_Data_in_Brief/BSE_microstructures/1000C_0.10h/1000C_0.10h_area3.tif]

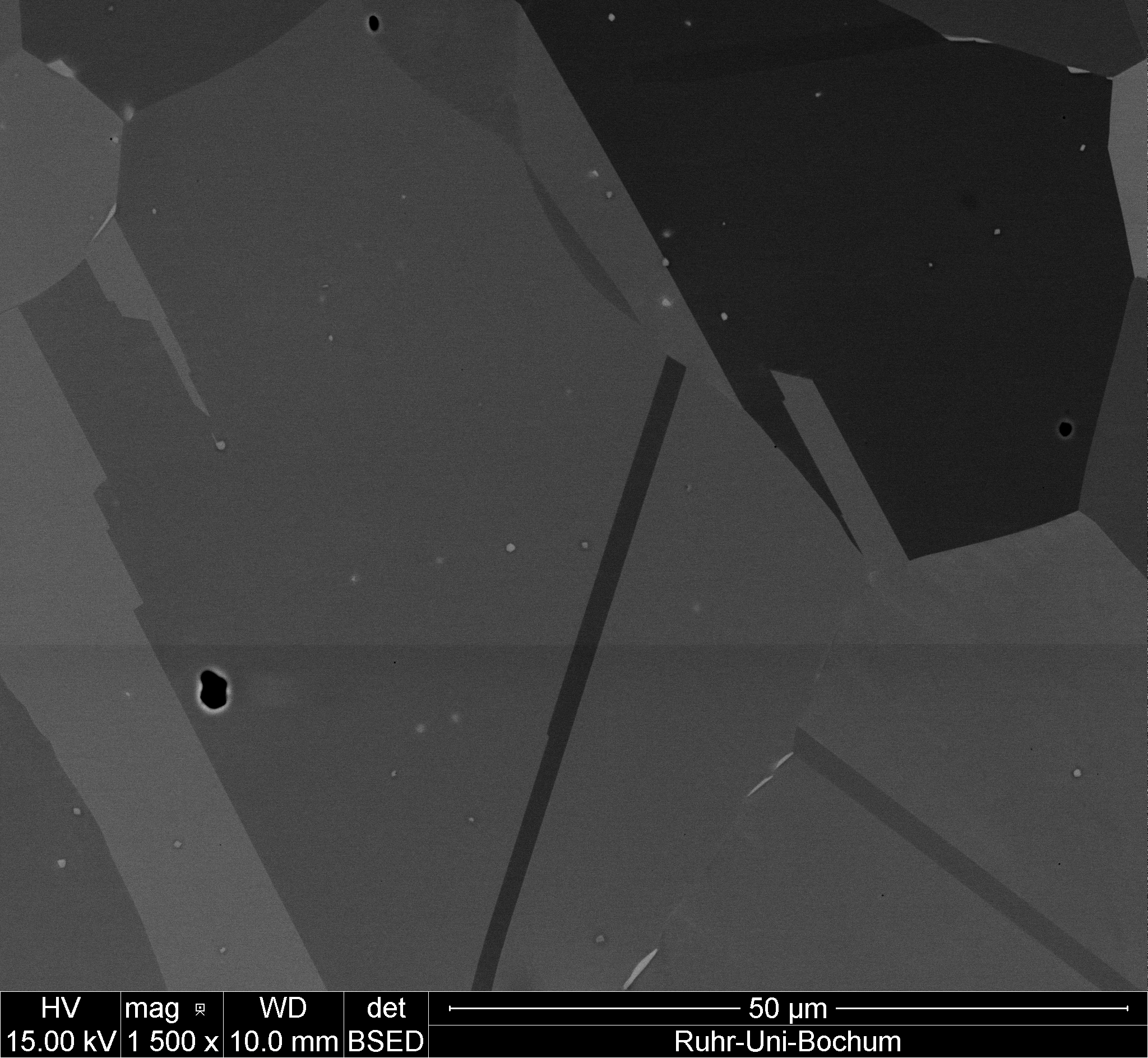

Supplement: Supplementary file 1 [file mmc1.zip › Upload_Data_in_Brief/BSE_microstructures/1000C_0.10h/1000C_0.10h_area4.tif]

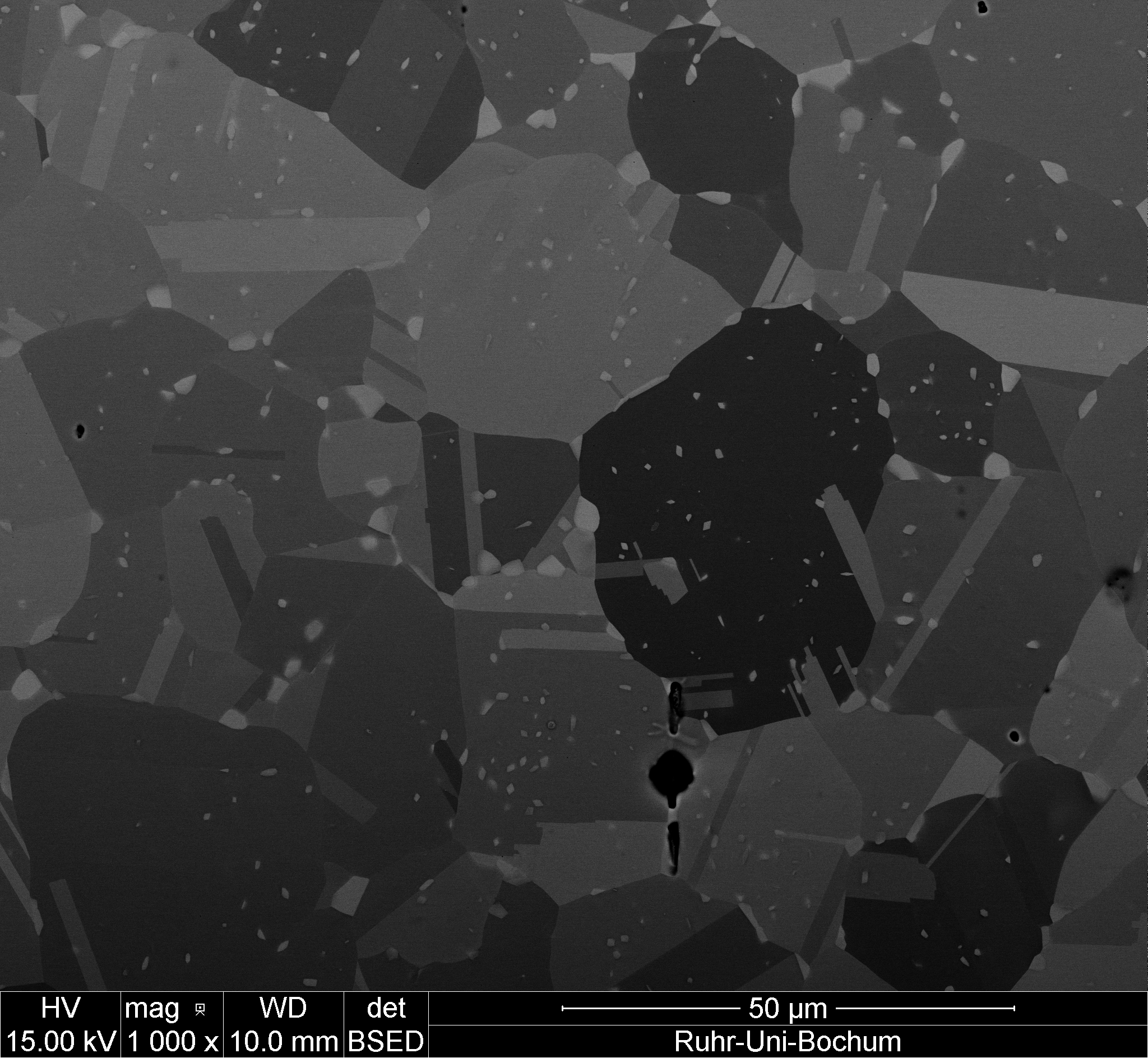

Supplement: Supplementary file 1 [file mmc1.zip › Upload_Data_in_Brief/BSE_microstructures/1000C_0001h/1000C_0001h_area1.tif]

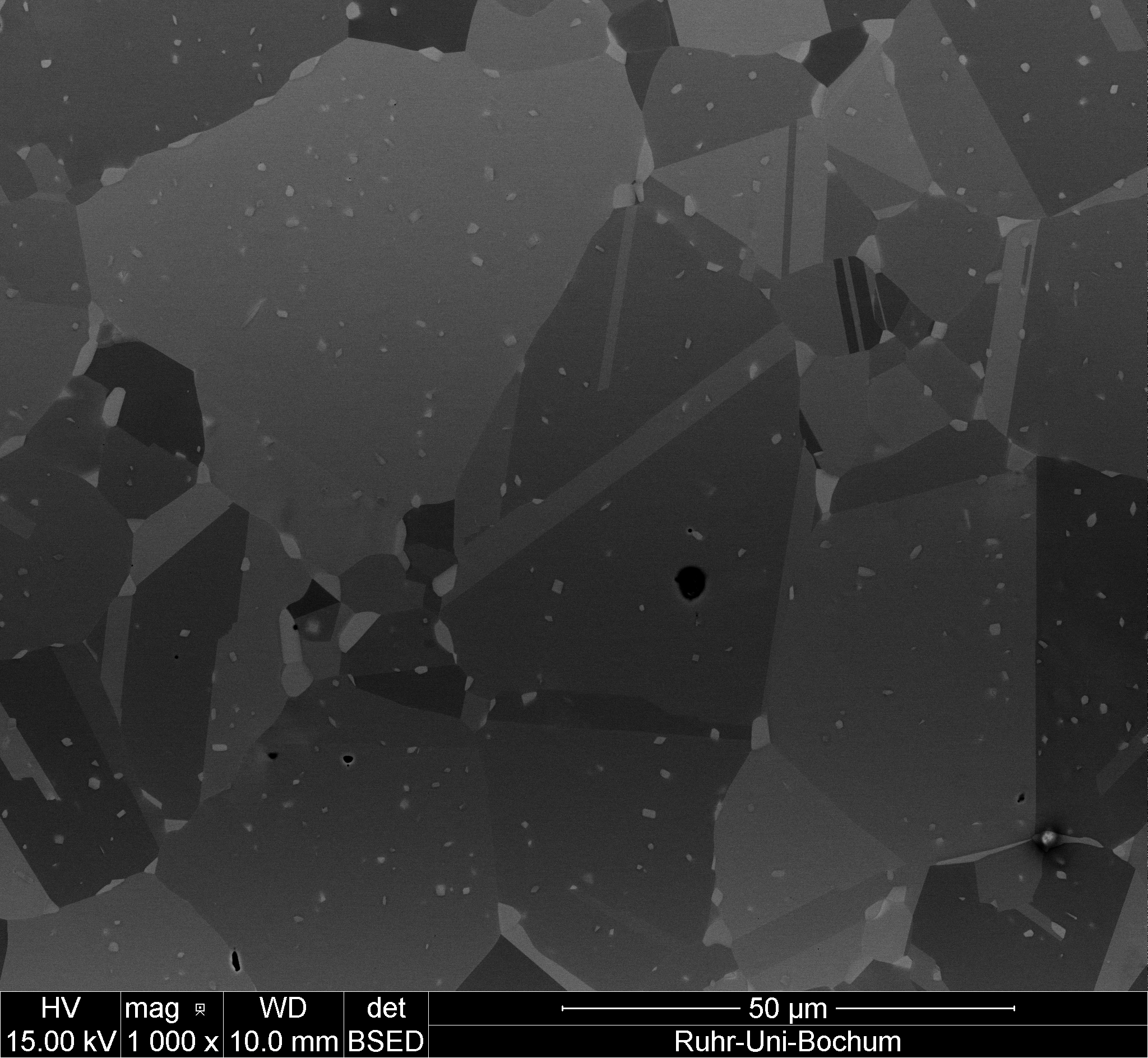

Supplement: Supplementary file 1 [file mmc1.zip › Upload_Data_in_Brief/BSE_microstructures/1000C_0001h/1000C_0001h_area2.tif]

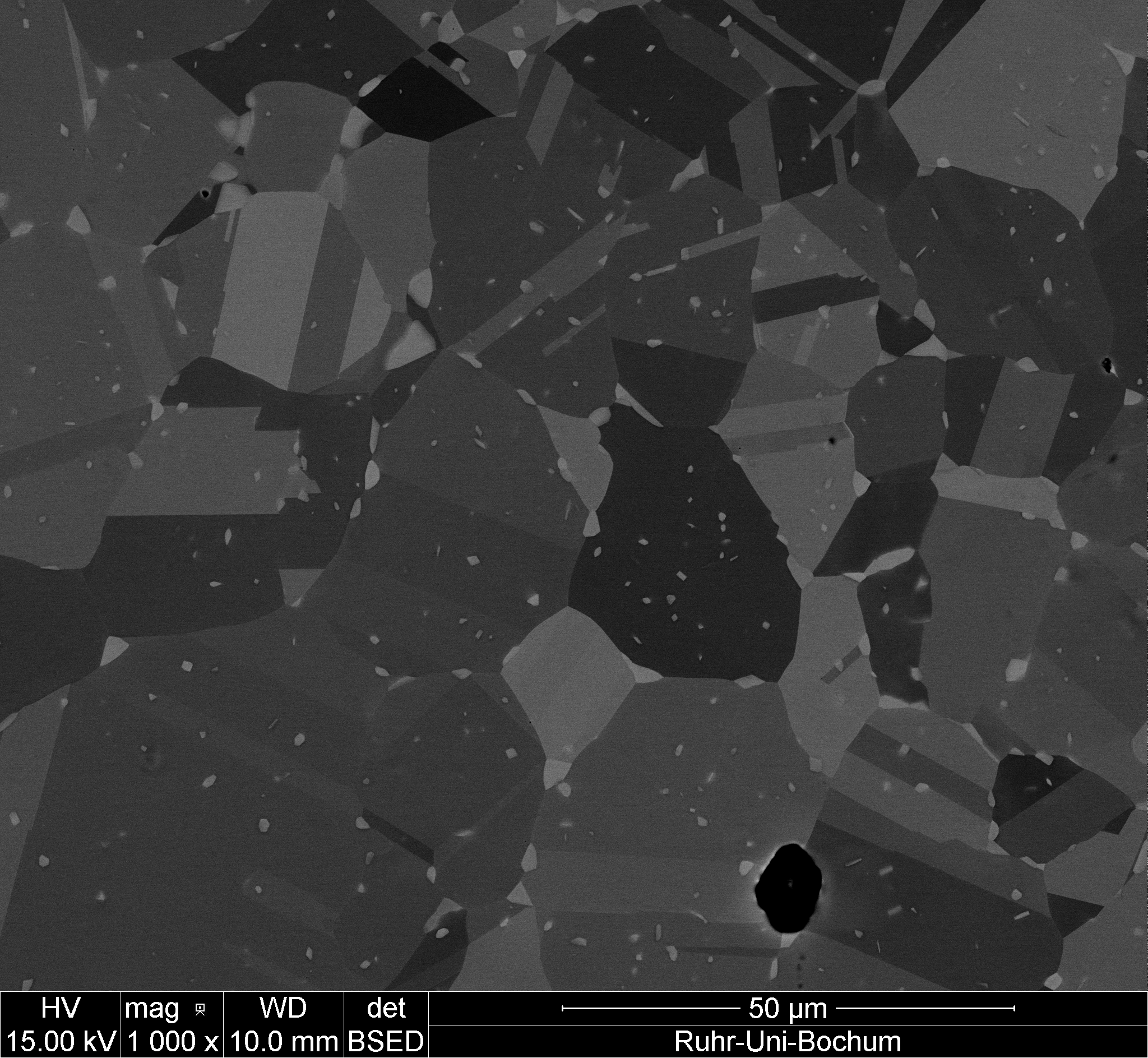

Supplement: Supplementary file 1 [file mmc1.zip › Upload_Data_in_Brief/BSE_microstructures/1000C_0001h/1000C_0001h_area3.tif]

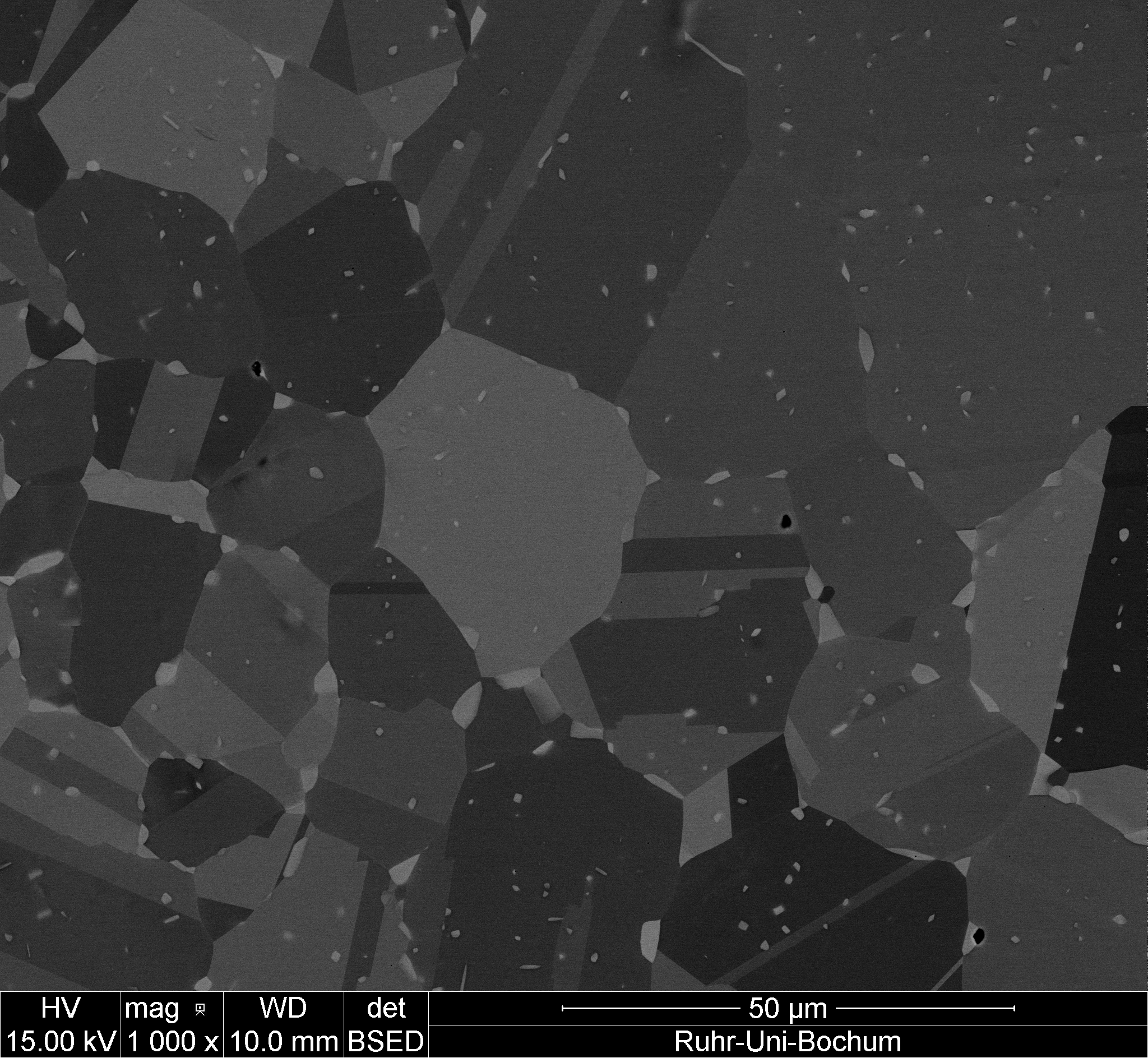

Supplement: Supplementary file 1 [file mmc1.zip › Upload_Data_in_Brief/BSE_microstructures/1000C_0001h/1000C_0001h_area4.tif]

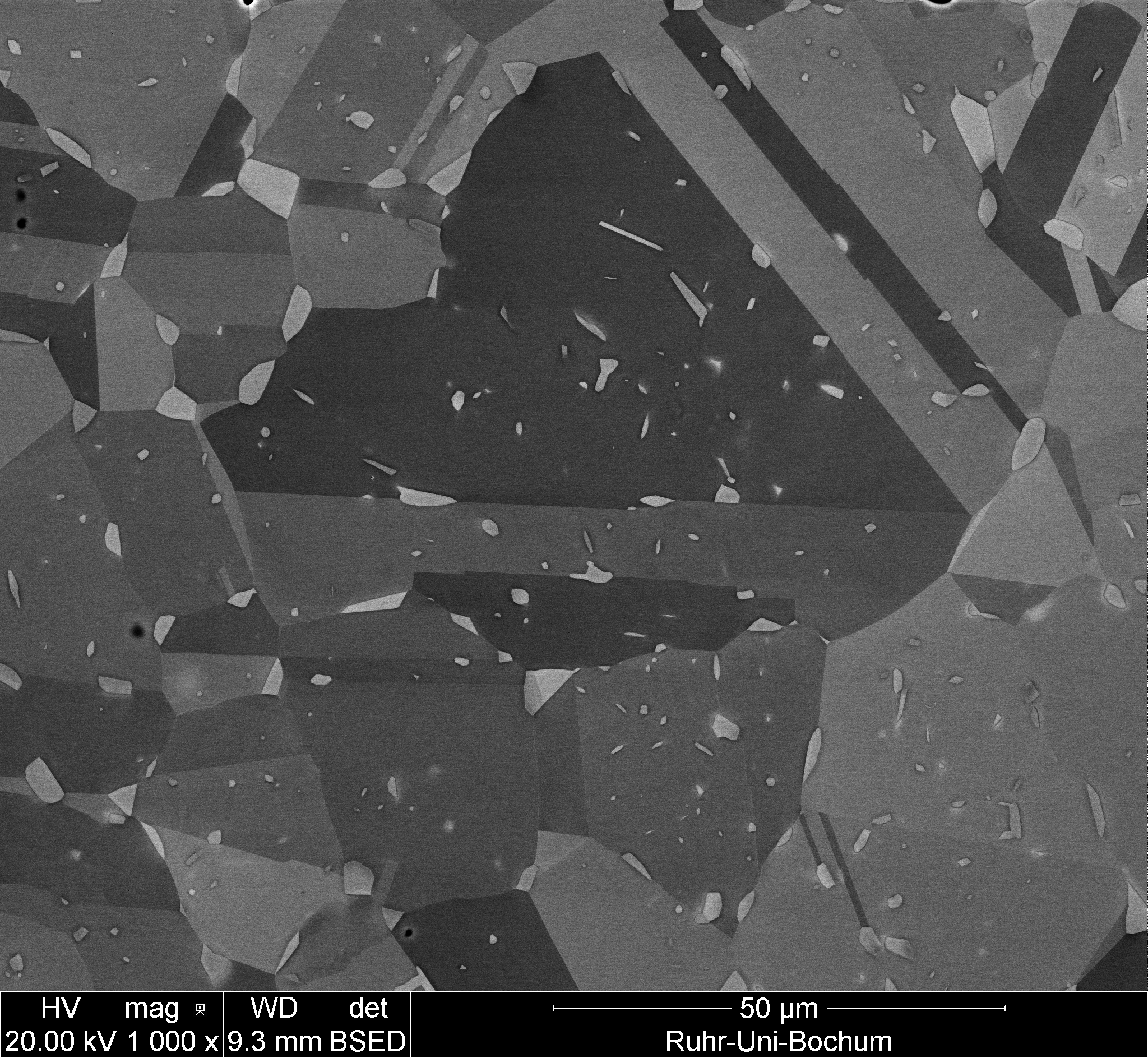

Supplement: Supplementary file 1 [file mmc1.zip › Upload_Data_in_Brief/BSE_microstructures/1000C_0010h/1000C_0010h_area1.tif]

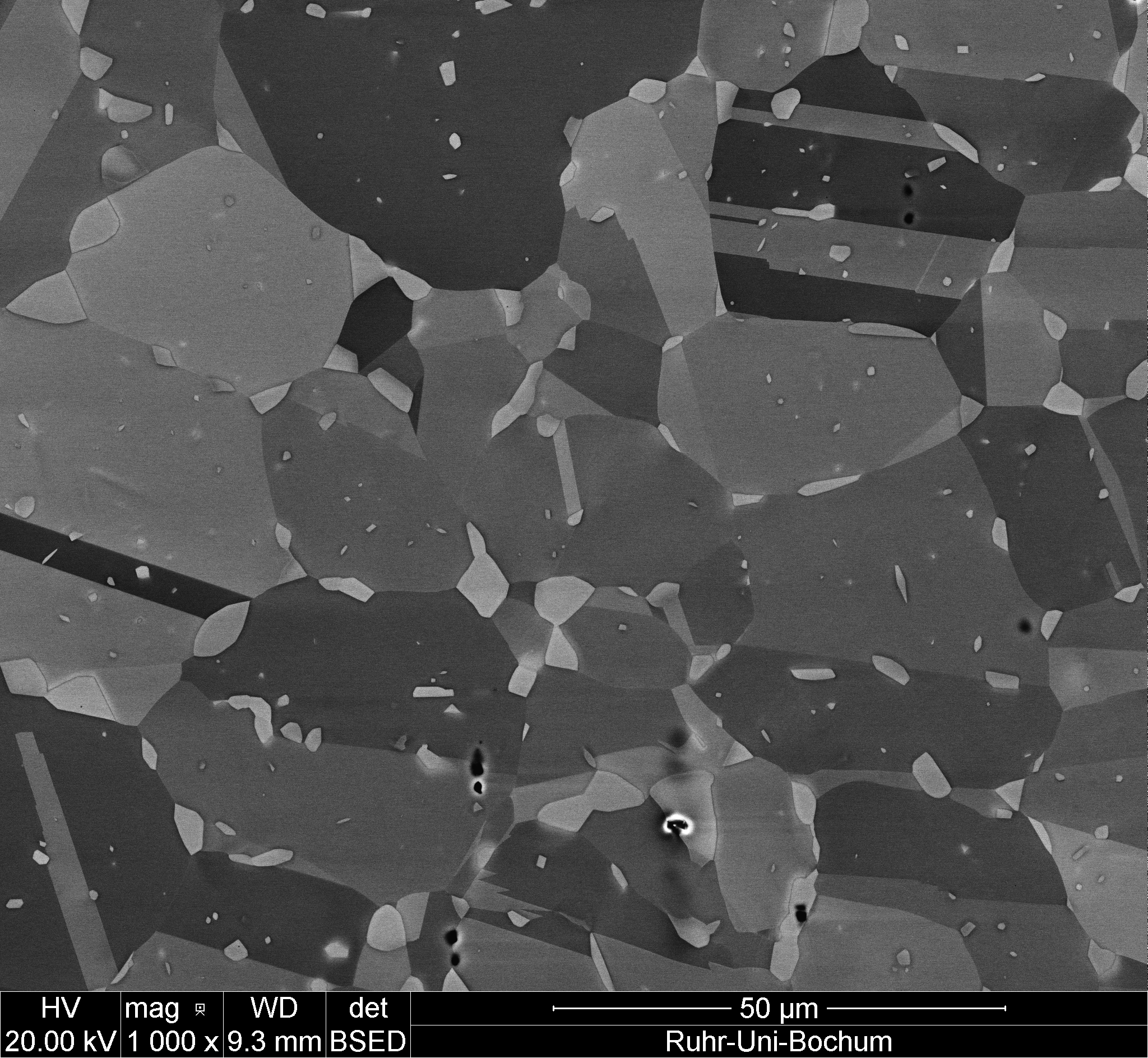

Supplement: Supplementary file 1 [file mmc1.zip › Upload_Data_in_Brief/BSE_microstructures/1000C_0010h/1000C_0010h_area2.tif]

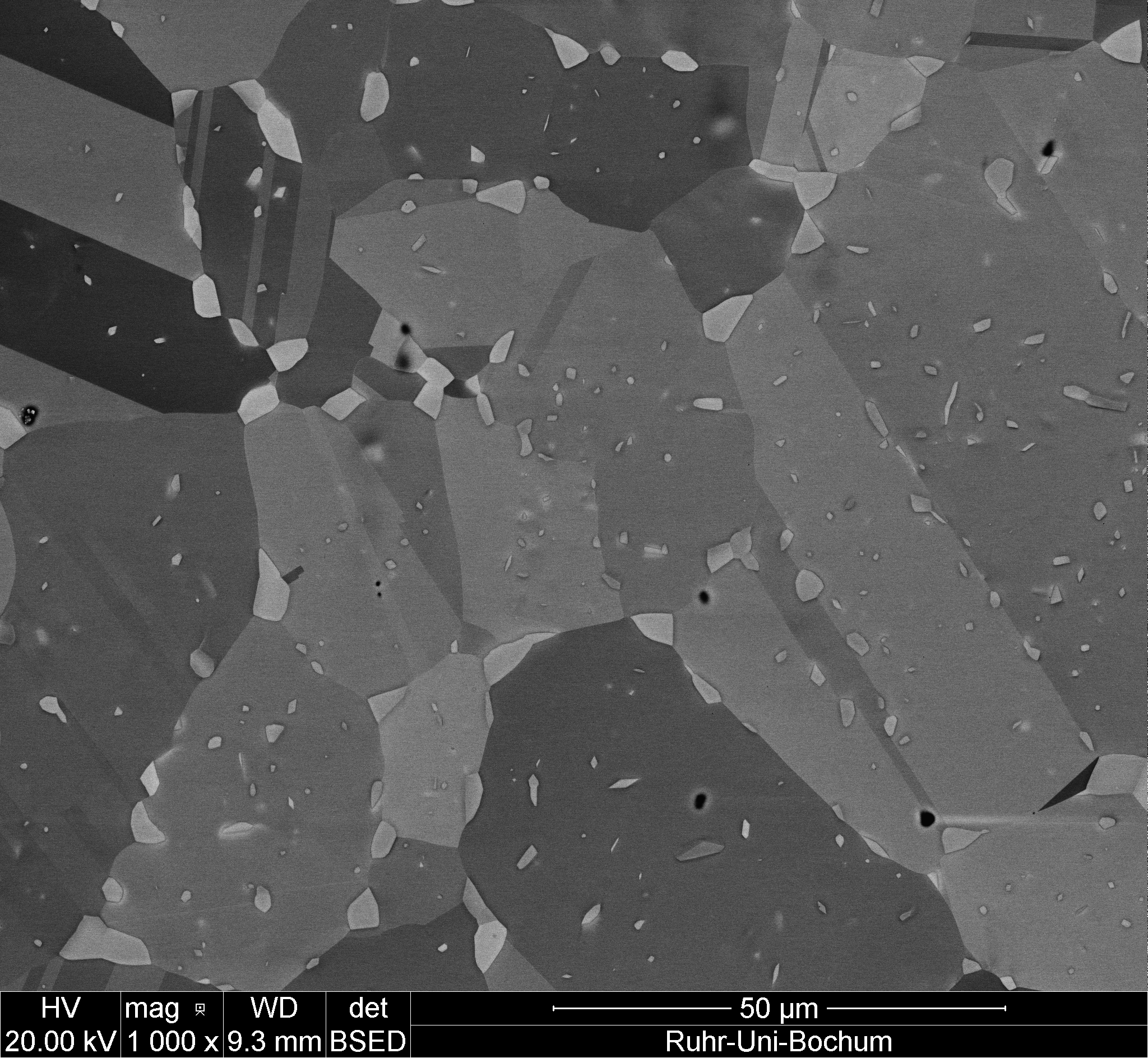

Supplement: Supplementary file 1 [file mmc1.zip › Upload_Data_in_Brief/BSE_microstructures/1000C_0010h/1000C_0010h_area3.tif]

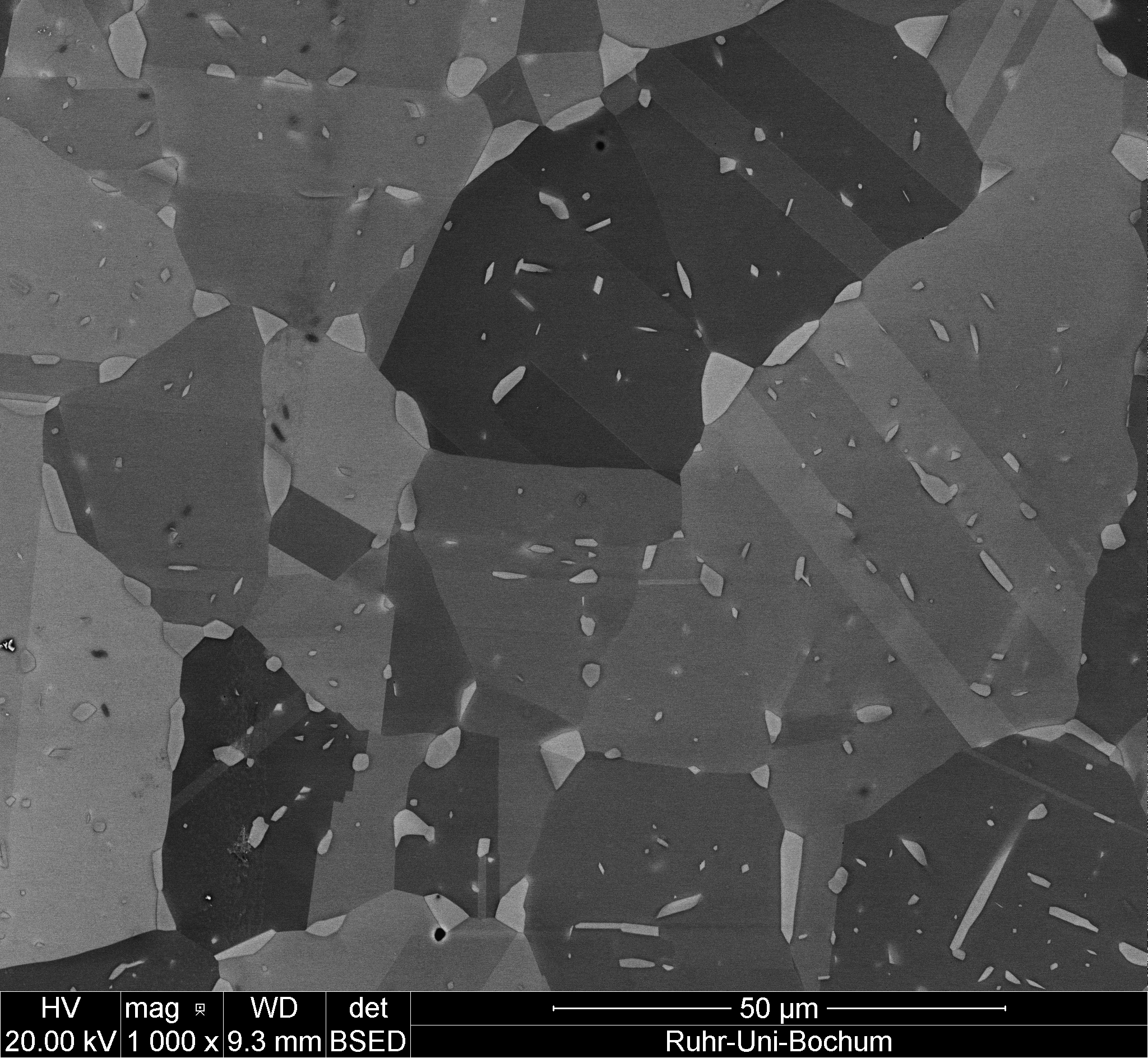

Supplement: Supplementary file 1 [file mmc1.zip › Upload_Data_in_Brief/BSE_microstructures/1000C_0010h/1000C_0010h_area4.tif]

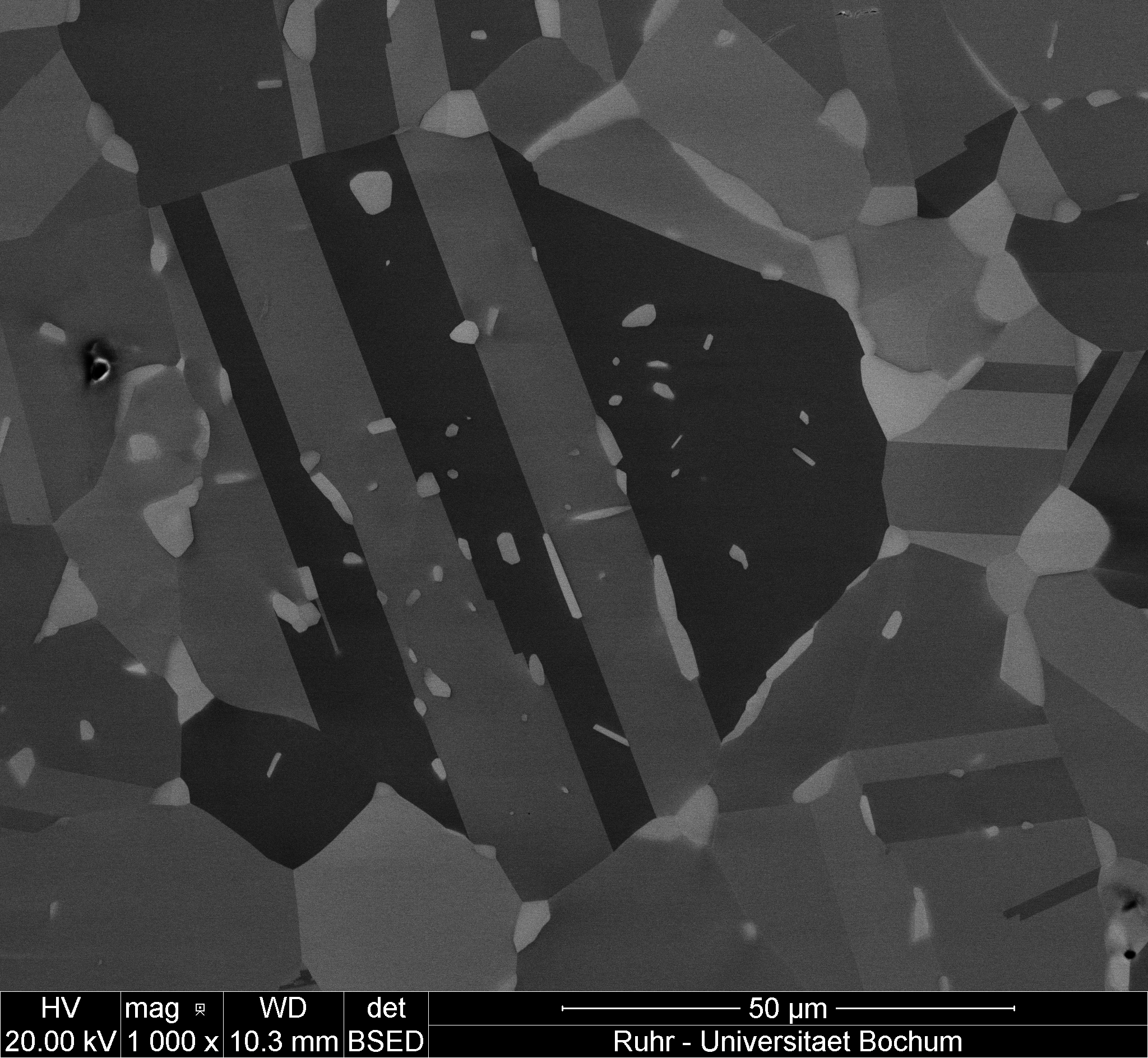

Supplement: Supplementary file 1 [file mmc1.zip › Upload_Data_in_Brief/BSE_microstructures/1000C_0100h/1000C_ 0100h_area1.tif]

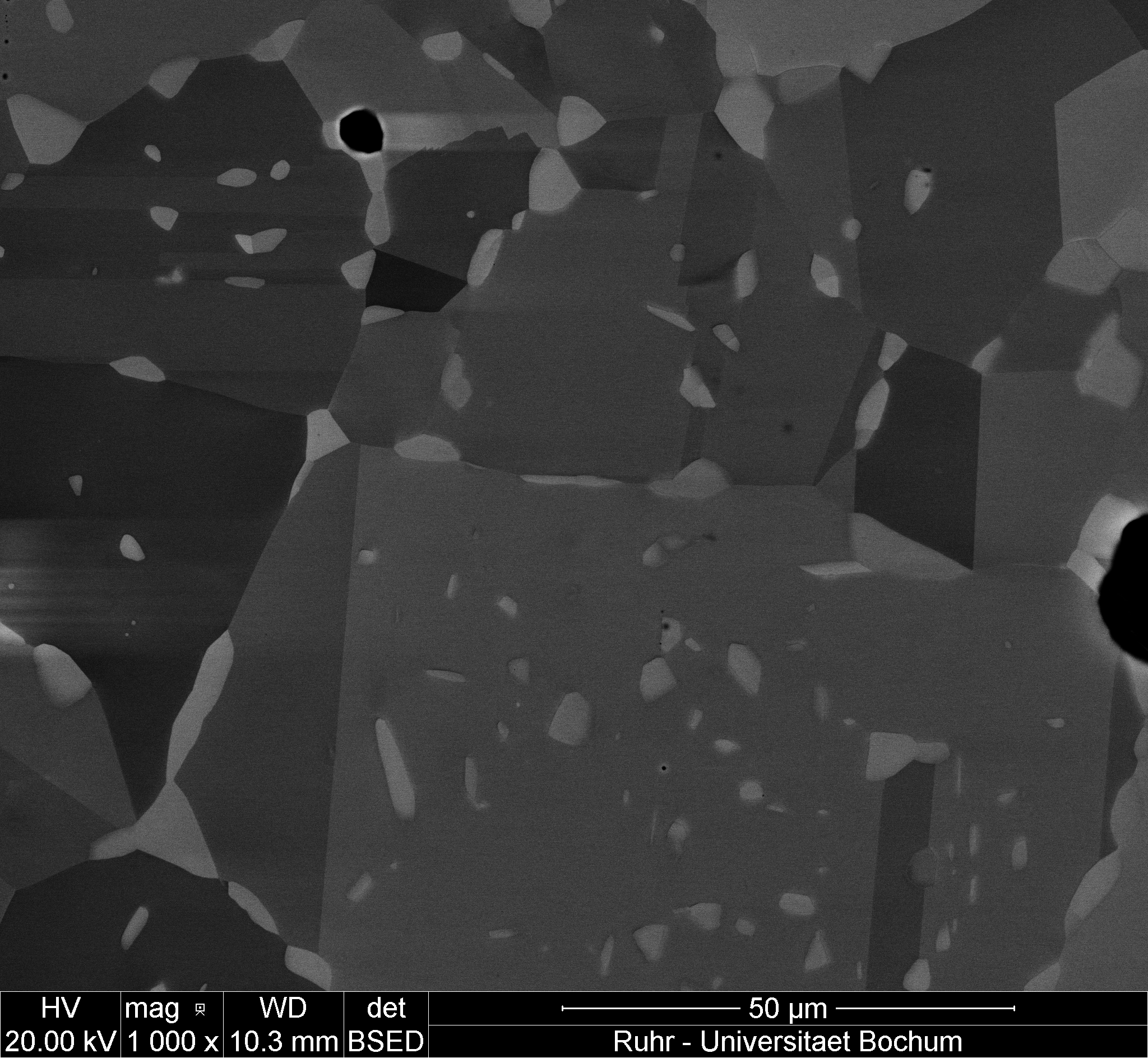

Supplement: Supplementary file 1 [file mmc1.zip › Upload_Data_in_Brief/BSE_microstructures/1000C_0100h/1000C_ 0100h_area2.tif]

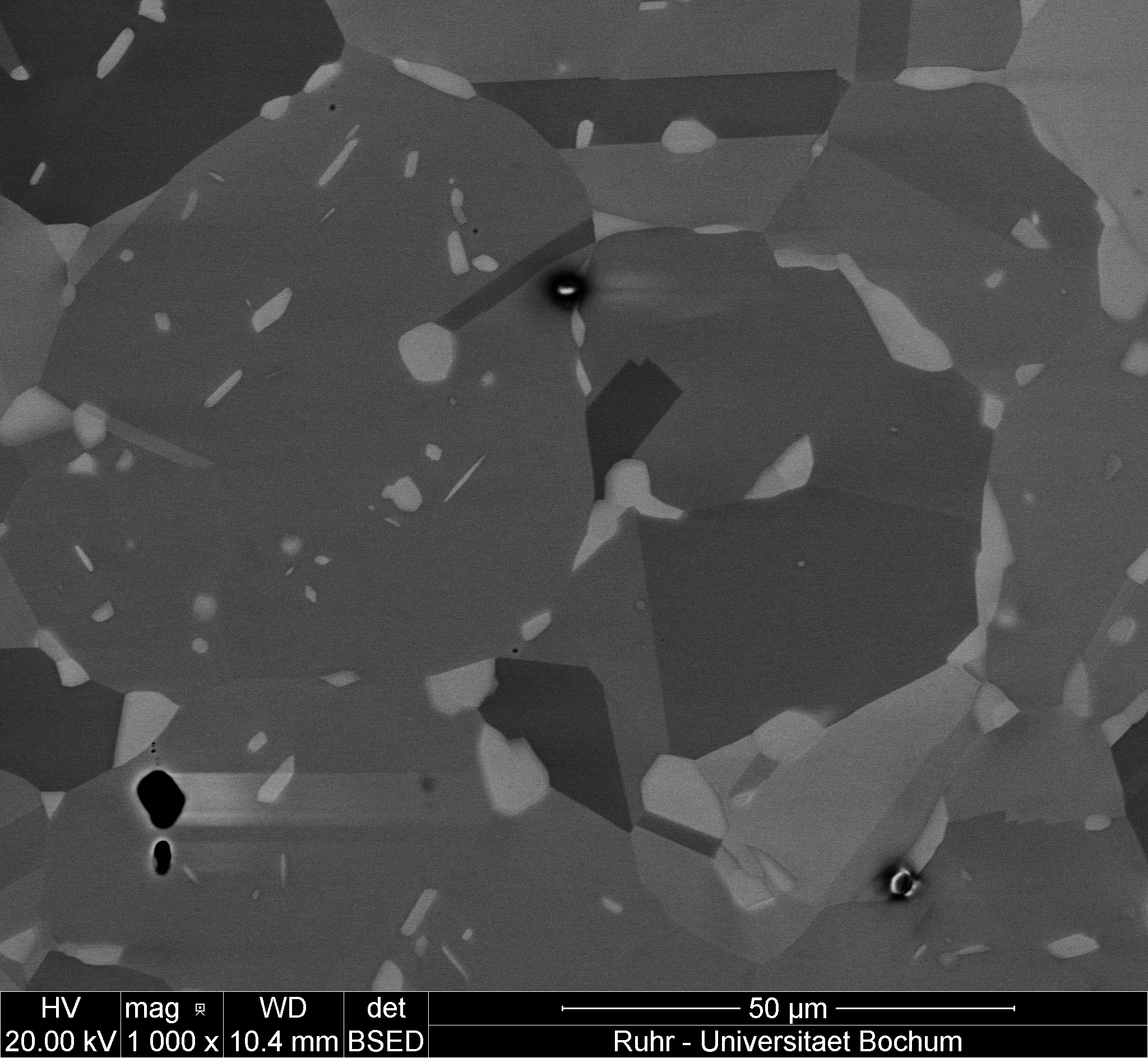

Supplement: Supplementary file 1 [file mmc1.zip › Upload_Data_in_Brief/BSE_microstructures/1000C_0100h/1000C_ 0100h_area3.tif]

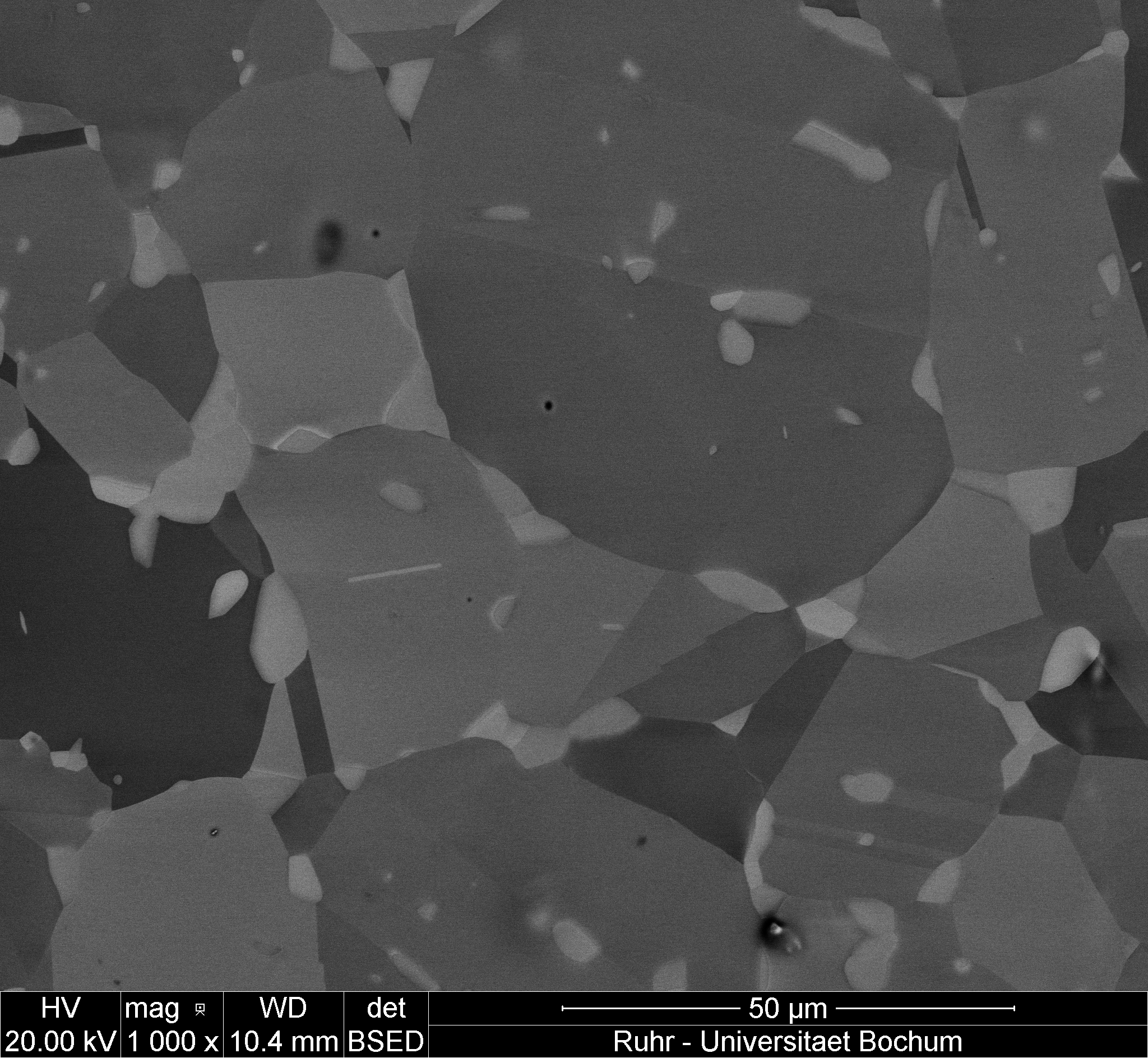

Supplement: Supplementary file 1 [file mmc1.zip › Upload_Data_in_Brief/BSE_microstructures/1000C_0100h/1000C_ 0100h_area4.tif]

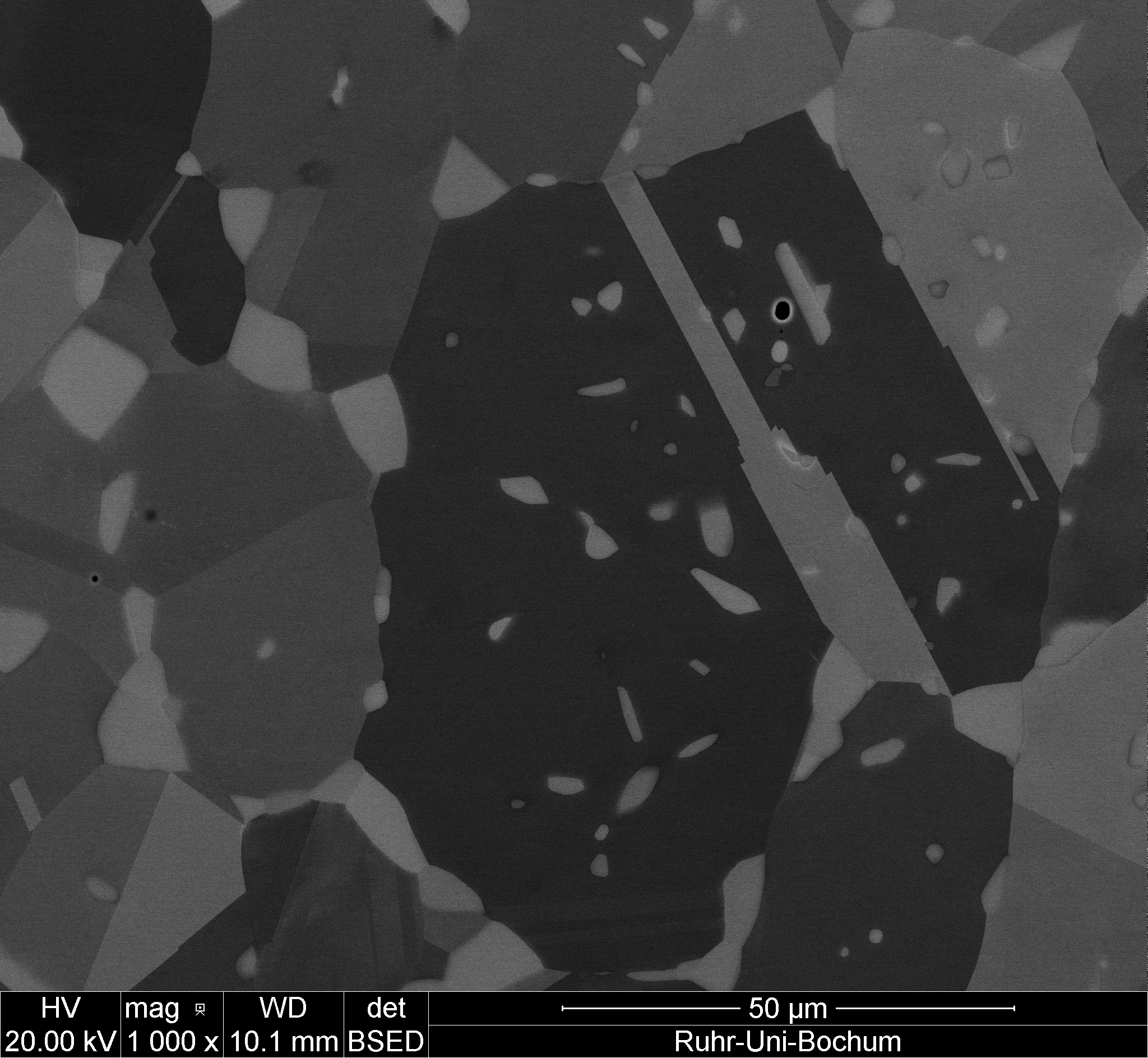

Supplement: Supplementary file 1 [file mmc1.zip › Upload_Data_in_Brief/BSE_microstructures/1000C_0500h/1000C_0500h_area1.tif]

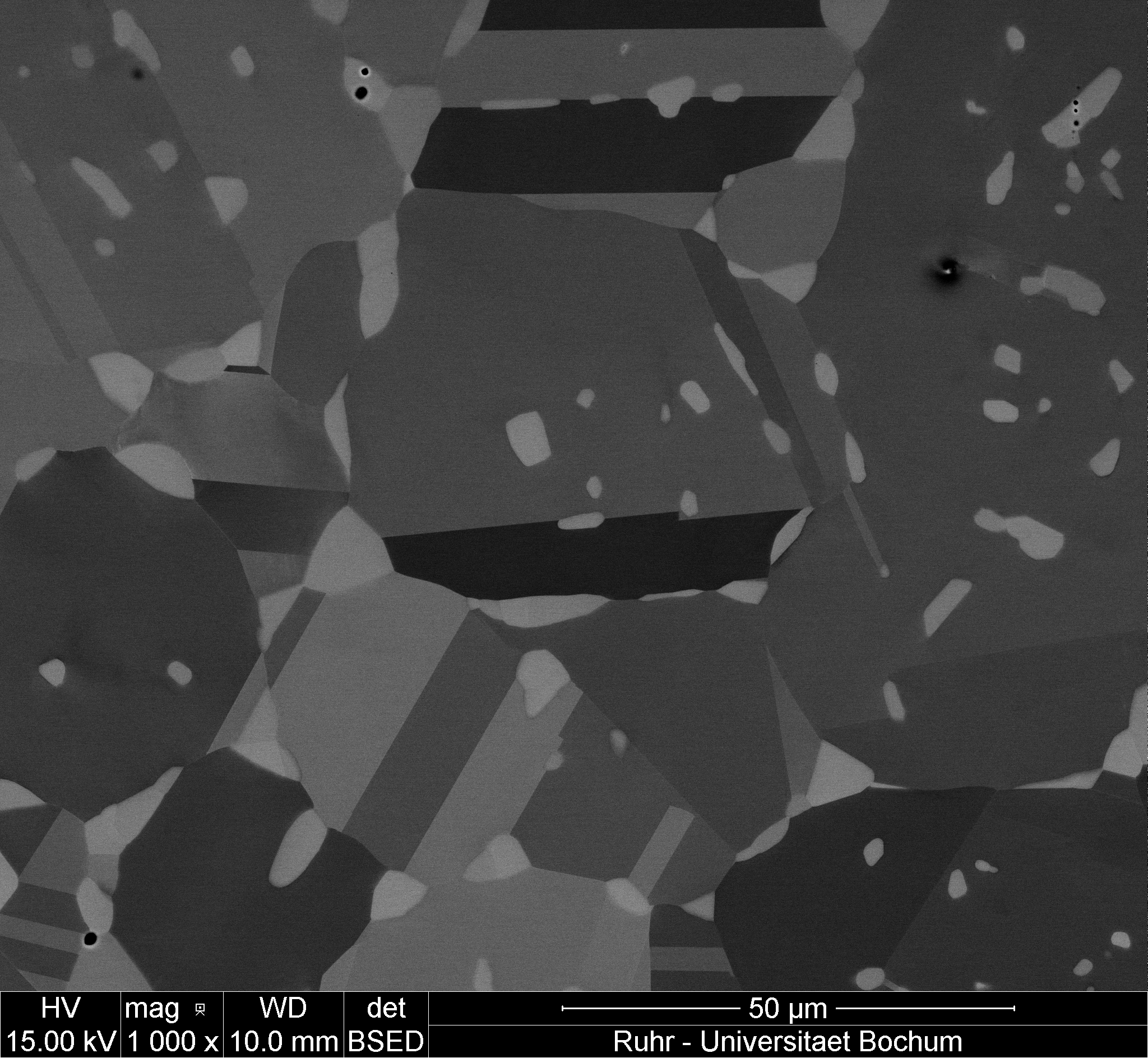

Supplement: Supplementary file 1 [file mmc1.zip › Upload_Data_in_Brief/BSE_microstructures/1000C_0500h/1000C_0500h_area2.tif]

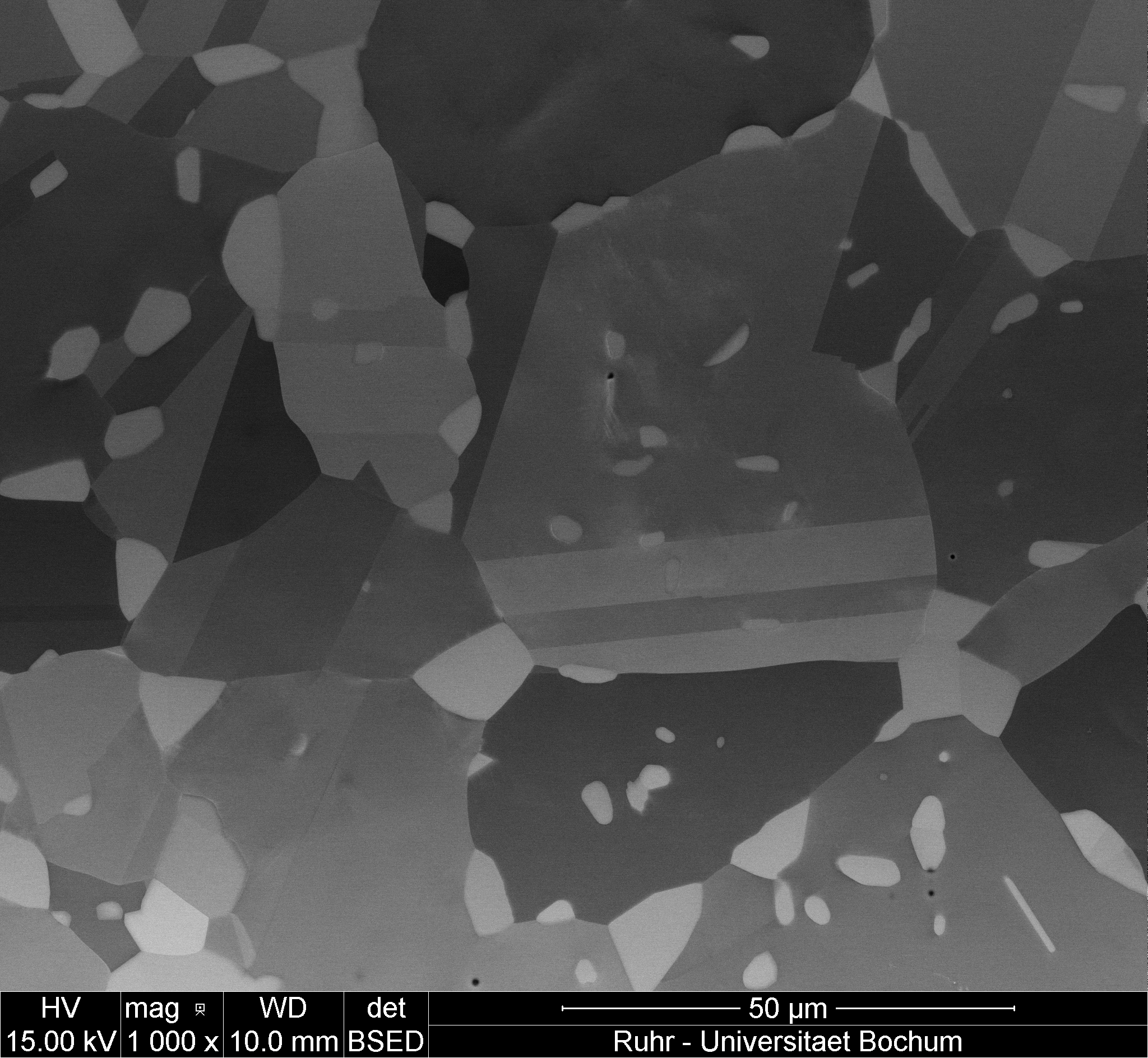

Supplement: Supplementary file 1 [file mmc1.zip › Upload_Data_in_Brief/BSE_microstructures/1000C_0500h/1000C_0500h_area3.tif]

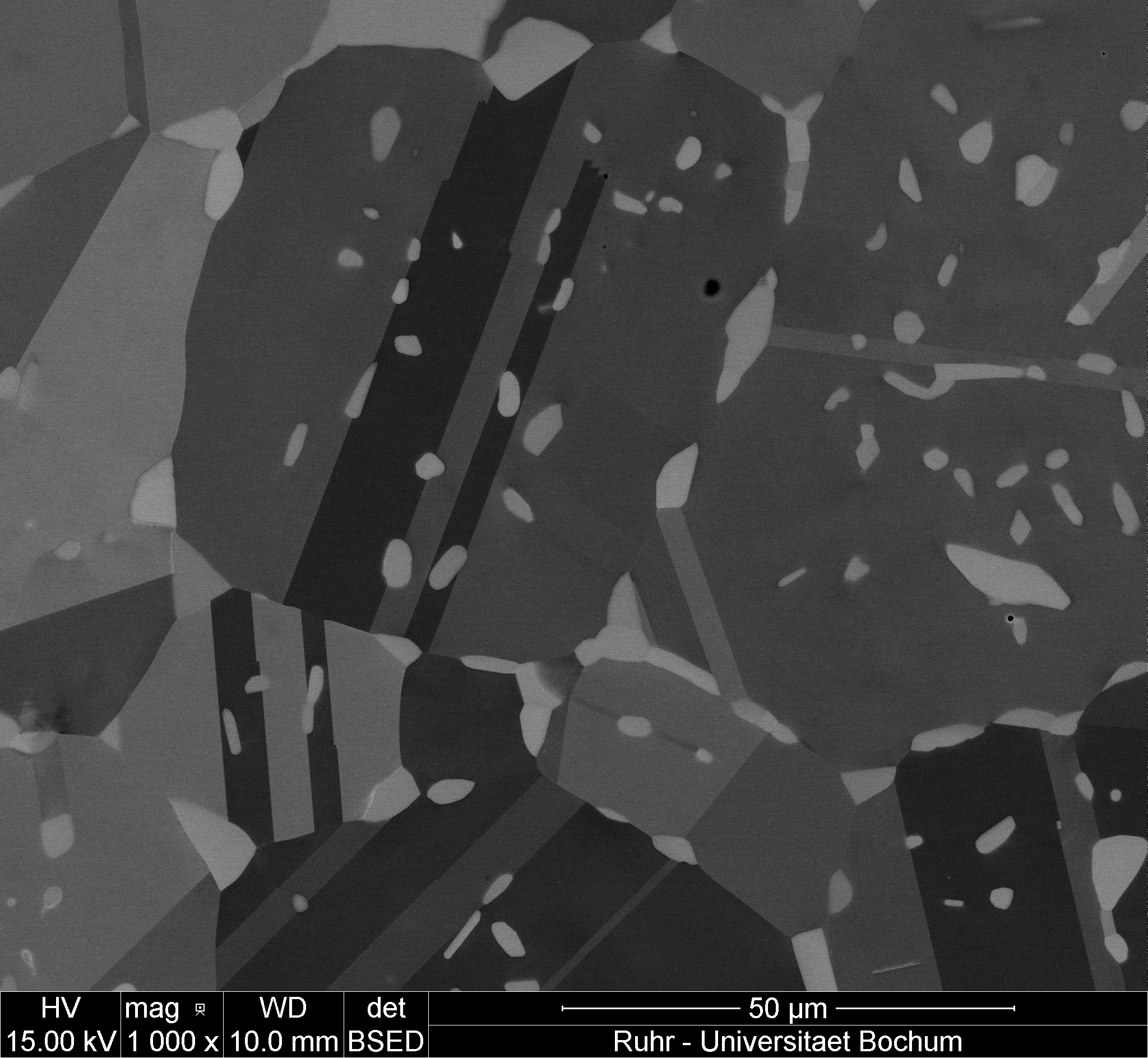

Supplement: Supplementary file 1 [file mmc1.zip › Upload_Data_in_Brief/BSE_microstructures/1000C_0500h/1000C_0500h_area4.tif]

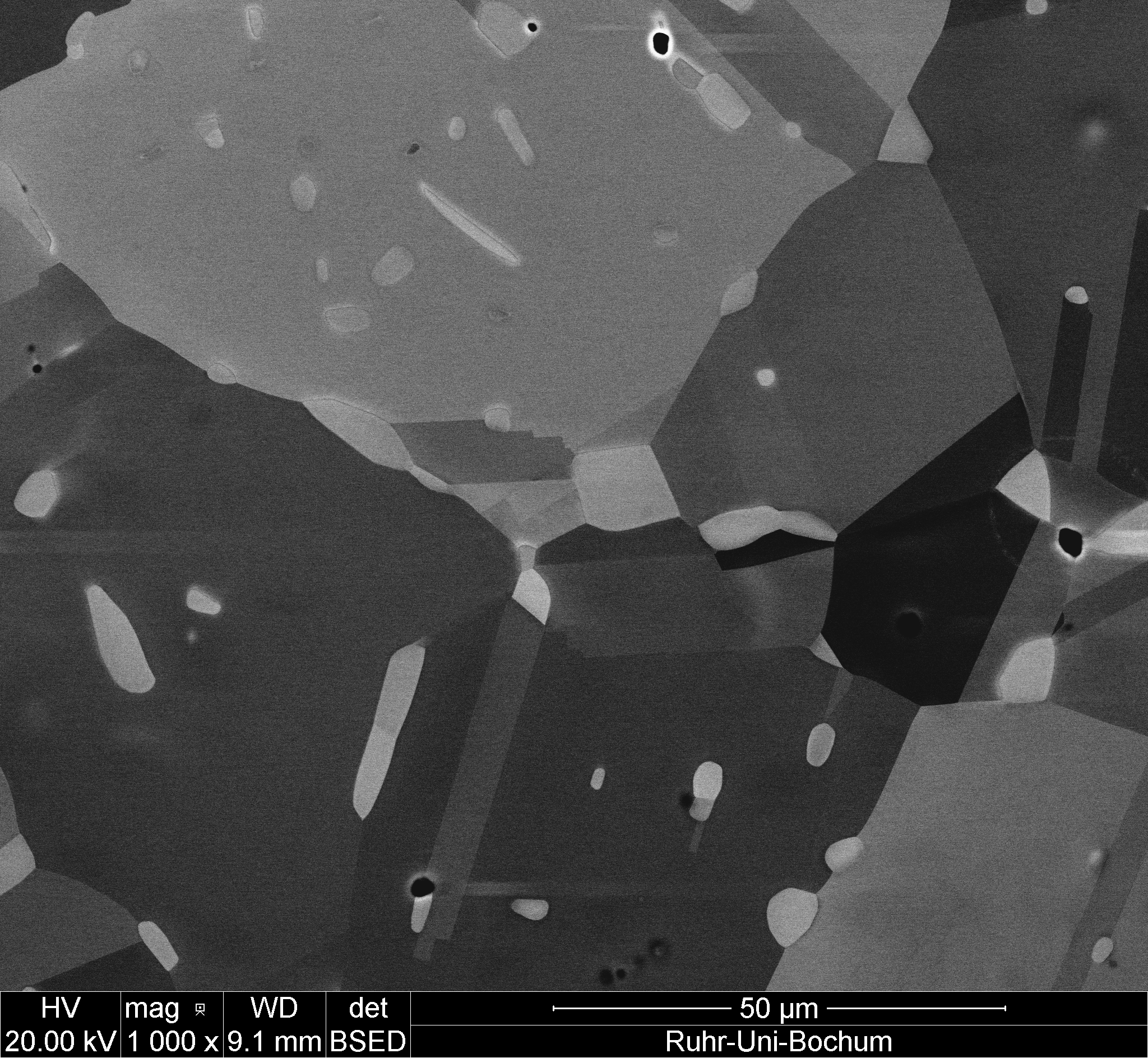

Supplement: Supplementary file 1 [file mmc1.zip › Upload_Data_in_Brief/BSE_microstructures/1000C_1000h/1000C_1000h_area1.tif]

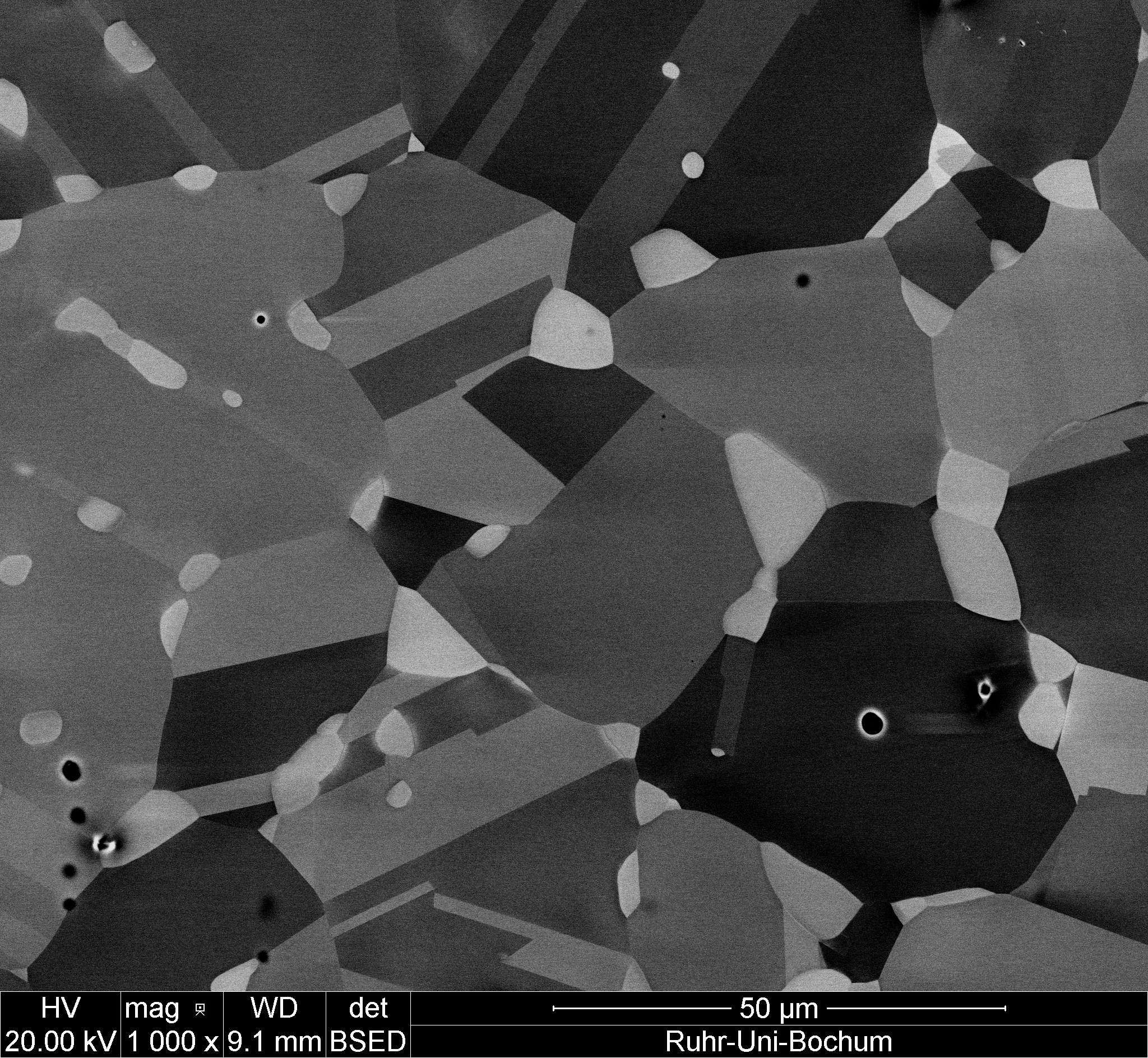

Supplement: Supplementary file 1 [file mmc1.zip › Upload_Data_in_Brief/BSE_microstructures/1000C_1000h/1000C_1000h_area2.tif]

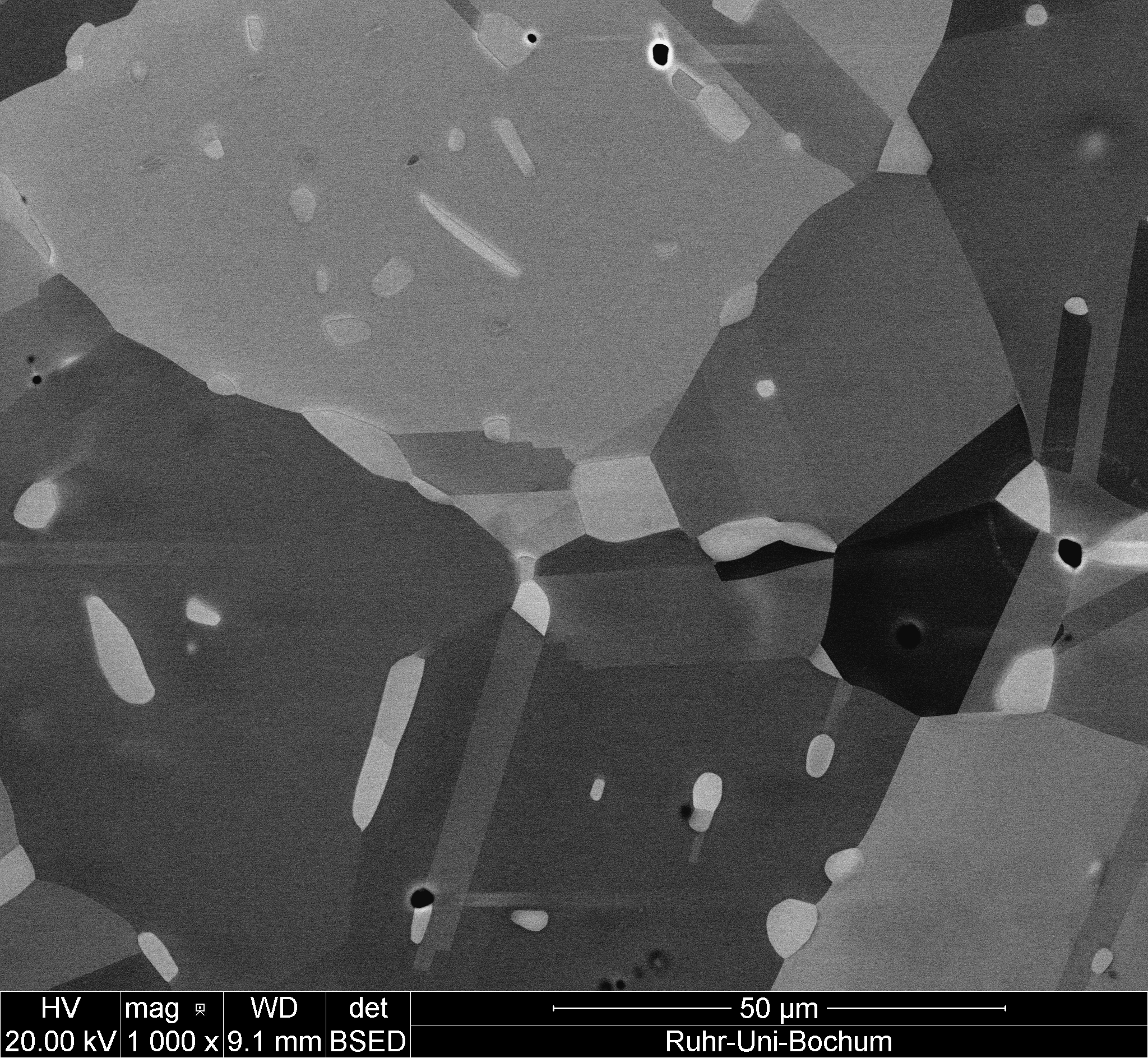

Supplement: Supplementary file 1 [file mmc1.zip › Upload_Data_in_Brief/BSE_microstructures/1000C_1000h/1000C_1000h_area3.tif]

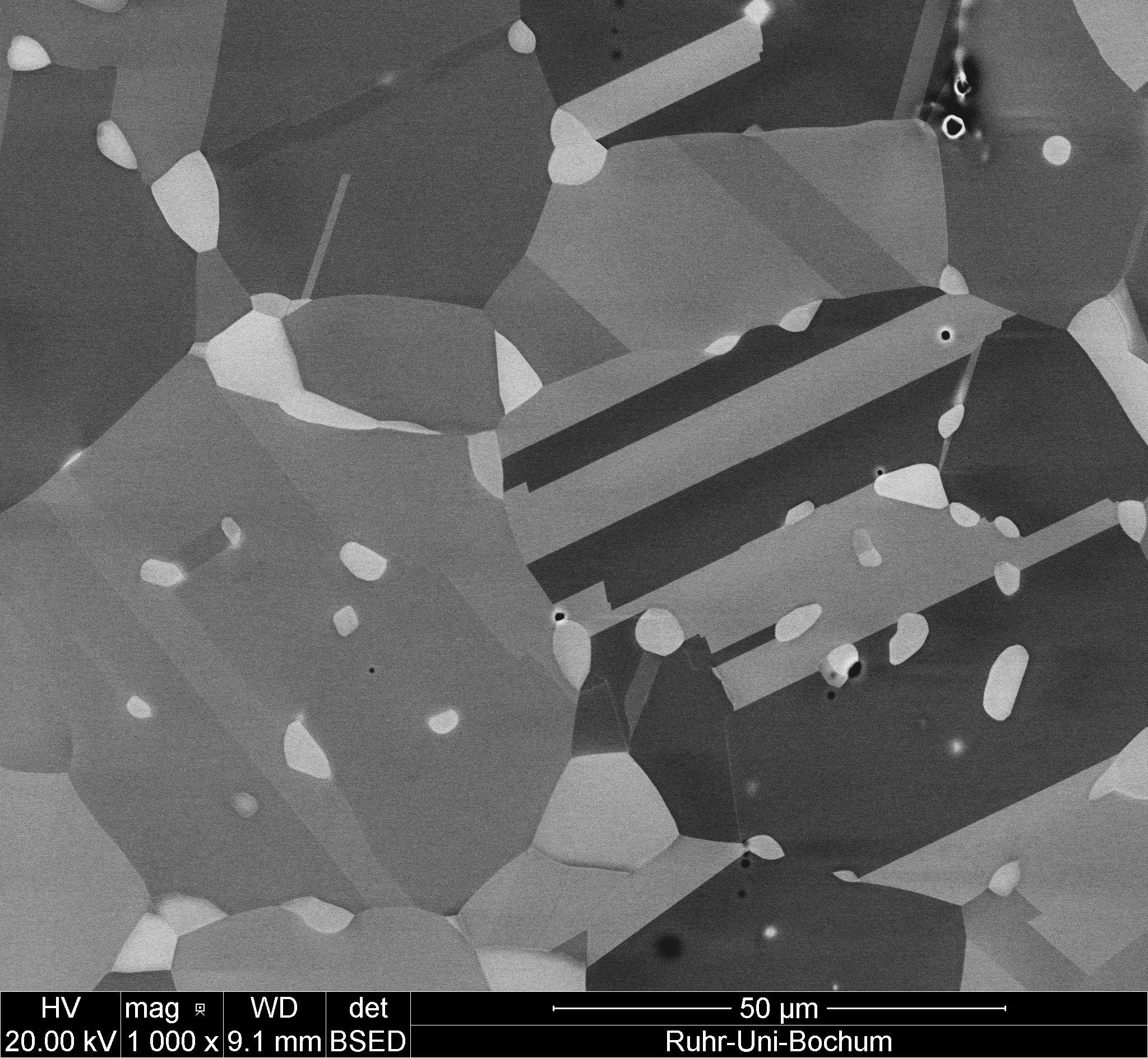

Supplement: Supplementary file 1 [file mmc1.zip › Upload_Data_in_Brief/BSE_microstructures/1000C_1000h/1000C_1000h_area4.tif]
